# The Nature of the Chemical Bonds of High-Valent Transition–Metal Oxo (M=O) and Peroxo (MOO) Compounds: A Historical Perspective of the Metal Oxyl–Radical Character by the Classical to Quantum Computations

**DOI:** 10.3390/molecules28207119

**Published:** 2023-10-16

**Authors:** Kizashi Yamaguchi, Hiroshi Isobe, Mitsuo Shoji, Takashi Kawakami, Koichi Miyagawa

**Affiliations:** 1 SANKEN, Osaka University, Ibaraki 567-0047, Osaka, Japan; 2 Center for Quantum Information and Quantum Biology (QIQB), Osaka University, Toyonaka 560-0043, Osaka, Japan; 3 Research Institute for Interdisciplinary Science, Okayama University, Okayama 700-8530, Okayama, Japan; h-isobe@cc.okayama-u.ac.jp; 4 Center for Computational Sciences, University of Tsukuba, Tsukuba 305-8577, Ibaraki, Japan; mshoji@ccs.tsukuba.ac.jp (M.S.); miyak@ccs.tsukuba.ac.jp (K.M.); 5 Department of Chemistry, Graduate School of Science, Osaka University, Toyonaka 560-0043, Osaka, Japan; kawakami@chem.sci.osaka-u.ac.jp

**Keywords:** M=O, MOO, iron oxide, iron peroxide, molecular oxygen, atomic oxygen, isolobal and isospin, oxyl-radical, BS and beyond BS methods, compound I and II, UNO X (X=CI; CC)

## Abstract

This review article describes a historical perspective of elucidation of the nature of the chemical bonds of the high-valent transition metal oxo (M=O) and peroxo (M-O-O) compounds in chemistry and biology. The basic concepts and theoretical backgrounds of the broken-symmetry (BS) method are revisited to explain orbital symmetry conservation and orbital symmetry breaking for the theoretical characterization of four different mechanisms of chemical reactions. Beyond BS methods using the natural orbitals (UNO) of the BS solutions, such as UNO CI (CC), are also revisited for the elucidation of the scope and applicability of the BS methods. Several chemical indices have been derived as the conceptual bridges between the BS and beyond BS methods. The BS molecular orbital models have been employed to explain the metal oxyl-radical character of the M=O and M-O-O bonds, which respond to their radical reactivity. The isolobal and isospin analogy between carbonyl oxide R_2_C-O-O and metal peroxide LFe-O-O has been applied to understand and explain the chameleonic chemical reactivity of these compounds. The isolobal and isospin analogy among Fe=O, O=O, and O have also provided the triplet atomic oxygen (^3^O) model for non-heme Fe(IV)=O species with strong radical reactivity. The chameleonic reactivity of the compounds I (Cpd I) and II (Cpd II) is also explained by this analogy. The early proposals obtained by these theoretical models have been examined based on recent computational results by hybrid DFT (UHDFT), DLPNO CCSD(T_0_), CASPT2, and UNO CI (CC) methods and quantum computing (QC).

## 1. Introduction

This review article describes a historical perspective of the elucidation of the nature of the chemical bonds of the high-valent transition metal oxo (M=O) and peroxo (MOO) compounds in chemistry and biology [1,2,3,4,5,6,7,8,9,10,11,12,13,14,15,16,17,18,19,20,21,22,23,24]. The iron-oxo species are assumed to be the active site of P450 enzymes and nonheme iron enzymes. Over past decades, both experimental and theoretical investigations have been performed to aid understanding, explanation and prediction of structure, bonding, and the reactivity of these complex compounds with high-valence states, such as Fe(V) and Fe(IV). From a theoretical viewpoint, both the static and dynamical electron correlation effects [25] play important roles for the 3d transition metal (M = Cr, Mn, Fe, Cu, etc.) oxo and peroxo compounds with quasi-degenerated electronic and spin states [26,27,28,29,30]. This means that the theoretical models explicitly involving electron–electron repulsion terms are indispensable for investigations into the nature of the chemical bonds of these systems. Historically, quantum-mechanical (QM) methods employed for them have been the unrestricted (U) semi-empirical and ab initio Hartree–Fock (HF) molecular orbital (MO) models, the Kohn–Sham (KS) density functional theory (DFT), and the hybrid HF-KS DFT (UHDFT) models based on a single Slater determinant approximation. These MO-based theoretical descriptions of the chemical bonds are often referred to as “*broken-symmetry (BS)*” methods, which have been employed as the first theoretical steps toward complex systems. 

Theoretically, the BS methods are mainly responding to the static electron correlations of M=O and MOO compounds. Therefore, the single reference (SR)- and multi-reference (MR)-coupled cluster (CC) single (S) and double (D) methods have been performed for remaining dynamical correlation corrections [25]. On the other hand, complete active space (CAS) configuration interaction (CI), CAS self-consistent field (SCF), and CASSCF second-order perturbation (PT2) have been also employed for extended computations of the M=O and MOO compounds [31,32]. The CAS CCS is formally equivalent to CASSCF. The natural orbitals (UNO) and occupation numbers obtained by the natural orbital analysis of BS solutions have been used for the construction of CAS [32]. Recently, CAS CI, CASSCF, CASPT2, MR CI, MR CC, etc., by use of UNO [25] have been performed beyond DFT computations of electronic and spin structures of metalloenzymes involving 3d transition metal complexes. Very recently, quantum computation (QC) has been proposed for accurate computations [33,34,35,36] of M=O and MOO compounds.

Transition metal enzymes play important roles in biological processes and reactions [37,38,39,40,41,42,43,44,45,46,47,48,49,50,51,52,53,54,55,56,57,58,59,60,61,62,63,64,65,66,67,68,69,70,71,72,73,74,75,76,77]; (1) oxygen carriers and storages in myoglobin (M = Fe), hemoglobin (M = Fe), hemocyanin (M = Cu), hemerythrin (M = Fe), (2) dioxygenations of phenol derivative, indole derivatives and others by dioxygenases (M = Fe, Cu), (3) mono-oxygenations of alkanes, alkenes, etc., by P450 (M = Fe, Mn) and non-heme iron (M = Fe, Mn) enzymes, (4) methane mono-oxygenation by methane monooxygenase (M = Fe, Cu), (5) water oxidation in photosystem II (PSII) (M = Mn), (6) oxygen reduction by cytochrome *c* oxidase (C*c*O), etc. These metalloenzymes have 3d transition metal oxo (M=O), peroxo (MOO), dinuclear metal oxides cores (M-(μ-O)-M) and (M-(μ-O)_2_-M), tetra-nuclear Mn clusters (Mn_4_O_x_), etc., which are embedded in the protein matrix. The protein matrix is often treated with the molecular mechanics (MM) model for computational economy; therefore, QM/MM methods have been used to elucidate the structure, function, and catalytic reactions of the metalloenzymes involving 3d M=O and MOO core complexes coupled strongly with the protein matrix of metalloenzymes. 

In the early 1980s, we initiated QM-theoretical investigations into the electronic and spin structures of 3d M=O and MOO model complexes, which are responsible for the above-mentioned biological functions and reactions [25,26,27,28,29,30]. The spin-polarized (SP) unrestricted Hubbard (UHB) and HF (UHF) models were applied to elucidate the electronic and spin structures of these model complexes with oxyl-radical reactivity. The UHF CCSD and UHDFT computations of the M=O species were performed to elucidate the binding energies between M and O, indicating the practical and handy applicability of UB3LYP (one of UHDFT) in the investigation of the transition-metal oxo compounds [25,78,79]. In a recent review [80], we summarized our computational results on Mn=O, Mn-O-Mn, MnO_2_Mn, Mn_4_O_4_, and CaMn_4_O_x_ (X = 5, 6) systems in relation to water oxidation by water oxidation complex in photosystem II (PSII). 

In this review, the historical development and perspective of theoretical elucidation of the nature of chemical bonds of high-valent M(X)=O (M = Mn, Fe; X = IV, V) and MOO species are mainly described for understanding and explanation of mono-oxygenations of cytochrome P450 enzymes and related heme iron-oxo systems [1,2,3,4,5,6,7,8,9,10,11,12,13,14,15,16,17,18,48,49,50,51,52,53,54,55,56,57,58,59,60,61,62,63,64,65,66,67,68,69,70]. To this end, the basic concepts and principles of the BS and beyond BS methods are briefly reviewed in relation to the theoretical modeling of electrophilic, homolytic radical, electron-transfer radical, and nucleophilic reactivity of these species, depending on the types of substrates and environmental conditions [25,26,27,28,29,30,81,82]. However, several excellent review articles [83,84,85,86,87,88,89,90] have already been published on the structure, bonding, and reactivity of P450 and related enzymes. Particularly, X-ray structures [88] and biological functions of P450 have been summarized in the book [21]. Therefore, our BS theoretical and computational results for M=O and MOO species in both heme- and non-heme systems are mainly reviewed in this article. Future perspectives are also touched on in relation to quantum computations (QC) [33,34,35,36] by using UNO obtained by the BS computations of metalloenzymes (UNO-QC) [25,26,36]. 

## 2. Historical Backgrounds for Mono-Oxygenation Reactions

### 2.1. Discoveries of Cytochrome P450 Enzymes and Related Metalloenzymes

First of all, the history of discovery of P450 is briefly reviewed as an introduction of P450 enzymes [1,2,3,4,5,6,7,8,9,10,11,12,13,14,15,16,17,18,19,20,21,37,38,39,40,41,42,43,44,45,46,47,48,49,50,51,52,53,54,55,56,57,58,59,60,61,62,63,64,65,66,67,68,69,70]. Cytochrome P450 (CYP) is an important enzyme for several biological reactions. The CYP is classified into several families, such as CYP*_m_*X*_n_* (*m*, X denote family and subfamily, respectively, and *n* denotes the name of each enzyme of the same group), depending on the structures of amino acid primary sequences. The CYP enzyme catalyzes the incorporation of only one oxygen atom of molecular oxygen into substrates, such as alkanes (RH), while reducing the other oxygen atom into a water molecule (H_2_O) with the following stoichiometry [1,2,3,4,5,6,7,8,9,10,11,12,13,14,15,16,17,18,19,20,21]; therefore, these reactions are often referred to as mono-oxygenations.

R–H + O_2_ + NAD(P)H + H^+^ → R–OH + H_2_O + NAD(P)^+^ (1)

The mono-oxygenation reactions in Equation (1) are different from the dioxygenase reactions with the following stoichiometry [22,23].

R + O_2_ → R–O_2_ (2)

where R is a carbon substrate and RO_2_ is an di-oxidized product. The first reports of cytochrome P450 by Omura and Sato were published in 1962~1964 [2,5,6,7,9]. Before their publications, Klingenberg discovered the carbon monoxide-binding pigment [1]. Hayaishi and Mason established the concepts of dioxygenases [22,23,24] and mono-oxygenase, namely, mixed-function oxidases [24] and/or external monooxygenase [22,23]. Cooper et al. [3,4,8] later elucidated the connection between cytochrome P450 and mixed-function oxidation using photochemical action spectra. 

**Figure 1:**
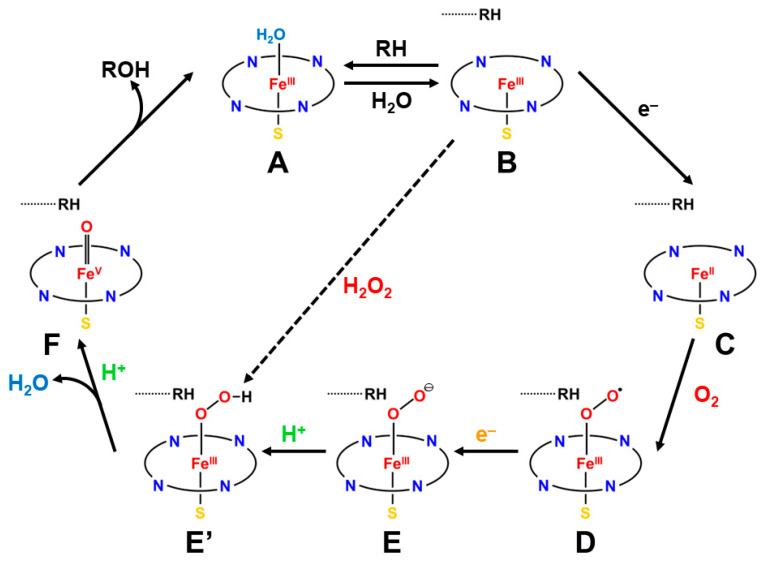
The reaction cycle (**A**–**F**) for the mono-oxygenation reactions of alkanes by cytochrome P450 [1,2,3,4,5,6,7,8,9,10,11,12,13,14,15,16,17,18,19,20,21]. The two electrons and one proton are provided by NAD(P)H and one proton by others (see details in the text). The shunt pathway from (**B**) to (**E’**) is feasible using peracids ROOH, and H_2_O_2_ instead of molecular oxygen (O_2_) [15,16,83,84,85,86,87,88,89,90].

Each cycle of mono-oxygenation illustrated in Figure 1 requires two electrons that originate from the pyridine nucleotides, NADH or NADPH [21,85], which is formed by photosynthesis [82]. The function of the electron transport protein of cytochrome P450 enzyme is to accept two electrons from NAD(P)H and to transfer them one at time to the cytochrome P450 during the mono-oxygenation reactions. Two classes of cytochrome P450 enzymes have been identified based on the electron transfer pathways. In one class, an N-terminal P450 heme domain is fused to a C-terminal NADPH, namely, cytochrome P450 reductase (CPR), which contains a flavin mononucleotide (FMN)-flavodoxin (FAD) and a FAD/NADPH binding domain. The electron transport chain of this class is expressed as follows [1,2,3,4,5,6,7,8,9,10,11,12,13,14,15,16,17,18,19,20,21]:

NADPH → FAD → FMN → Heme (P450)(3a)

where NADPH and FMN are two electron (2e^−^) and one electron (e^−^) donors, respectively. Therefore, FMN transfers two electrons by NADPH one at time at the necessary steps, as illustrated in Figure 1. 

The one electron mediator FMN is replaced with an adrenodoxin, 2Fe-2S ferredoxin-type iron-sulfur protein, in other class of P450 enzymes. The electron transfer process of the class is expressed as follows [1,2,3,4,5,6,7,8,9,10,11,12,13,14,15,16,17,18,19,20,21].

NADPH → FAD → 2Fe-2S → Heme (P450)(3b)

The NAD(P)H or NADH provides one proton (H^+^) and therefore one more necessary H^+^ is transferred in the cytochrome P450 enzyme systems, as shown in Figure 1. Iron-sulfur (Fe-S) clusters, such as ferredoxin, play important roles for electron transfer reactions in biology. Therefore, the theoretical investigations of 2Fe-2S, 3Fe-4S, and 4Fe-4S clusters are also very important for the elucidations of redox reactions in biology [33,34,35,36,91,92].

The reaction cycle of the mono-oxygenation with cytochrome P450 is now elucidated chemically [21,83,84,85,86,87,88,89,90]. Figure 1 illustrates the well-established molecule-based reaction cycle of mono-oxygenation reactions by cytochrome P450 [1,2,3,4,5,6,7,8,9,10,11,12,13,14,15,16,17,18,19,20,21,48,49,50,51,52,53,54,55,56,57,58,59,60,61,62,63,64,65,66,67,68,69,70,71,72,73,74,75,76,77,78,79,80,81,82,83,84,85,86,87,88,89,90]. The resting state (**A**) is expressed by the porphyrin (Por) LFe(III) (L = axial thiolate anion) state with a distal coordinated water molecule, namely, the sixth ligand to iron. The low-spin (*S* = 1/2) state of iron is favored in the **A** state because of the coordination of water molecule. The coordinated water molecule is replaced with substrate alkane (RH) at the next step (**B**). The high-spin (*S* = 5/2) ground state of the PorLFe(III) species is formed at this step (**B**), changing the redox potential for electron capture. In fact, the one-electron transfer (OET) from cytochrome P450 reductase (CPR) becomes feasible, providing the one electron trapped intermediate (**C**) with a PorLFe(II) electronic configuration. 

The molecular oxygen is inserted at the Fe(II) site, affording the Fe-peroxide intermediate (**D**) with PorLFe(III)OO. The further one-electron transfer from CPR provides the reduced intermediate (**E**) with PorLFe(III)OO^−^. The proton transfer occurs to afford the protonated intermediate (**E’**) PorLFe(III) with the hydroperoxide anion, which is often referred to as Compound 0. The heterolysis of the OOH bond of **E’** is induced by the protonation, providing the formal PorLFe(V)=O intermediate in step **F**, which is transformed into π cation radical Por(^+^•)LFe(IV)=O intermediate when the axial ligand (L) is appropriate ligands [1,2,3,4,5,6,7,8,9,10,11,12,13,14,15,16,17,18,19,20,21]. The **F** is referred to as cytochrome P450 compound I (P450-CPI) [56,57,58,59,60,61,62]. The formal iron-oxo intermediate Fe(V)=O^3+^ formed at the step **F** undergoes the mono-oxygenation, as illustrated in Figure 1 [24]. The hydrogen radical abstraction (HRA) mechanism by **F** was proposed based on the large intermolecular kinetic isotope effect (k_H_/k_D_ > 11), indicating a radical reactivity [21]. However, other intermediates [65,66,67,68,69,70,71] were also proposed for mono-oxygenations by **F**.

Extensive investigations have been performed into PorLFeOO compounds with several kinds of axial ligand (L) [37,38,39,40,41,42,43,44,45]. The axial ligand is also the thiolate anion of chloride peroxidase that undergoes a catalytic chlorination of alkane, as shown in Equation (4).

2R−H + 2Cl^−^ + H_2_O_2_ → 2R–Cl + 2H_2_O (4)

On the other hand, the axial ligand (L) is histidine (His) in the case of peroxidase (M = Fe), which catalyzes the decomposition reaction of hydroperoxide into molecular oxygen and water as follows:

2H_2_O_2_ → O_2_ + 2H_2_O (5)

The axial ligand (L) is also histidine (His) in the case of myoglobin (Mb) and hemoglobin (Hb) [83]; however, the H-N bond of histidine in peroxidase is linked with the proton-accepting amino acid to form its anion in a sharp contrast to Mb and Hb. The same reaction in Equation (5) is also catalyzed by catalase (M = Fe) with L = phenolate anion arising from tyrosine. The shunt pathway from B to E’ using per-acids ROOH and H_2_O_2_ has been investigated to elucidate chemically the reaction cycle in Figure 1 [15,16,83,84,85,86,87,88,89,90].

In the 1970s~1980s, magnetic susceptibility and EPR experiments [37,38,39,40,41,43,44] elucidated open-shell electronic structures of PorLFeOO. The magnetic property of oxygen carriers such as hemoglobin was investigated in Prof. Kotani’s Lab. at that time [39]. The biological functions of P450 enzymes were extensively investigated in Prof. Sato’s lab at the protein institute at Osaka University. In the early 1980s, we initiated theoretical investigations of the nature of the chemical bonds of the high-valent transition metal oxo and peroxo compounds in the metalloenzymes [25,26,27,28,29,30]. The SP BS UHF models [93,94], followed by small UNO CAS CI [95], were employed as a first step to investigate metal oxides because multi-reference theoretical models [31,32] were hardly applicable to them at that time. Other groups [96,97] employed the BS *Xα* model for theoretical investigations of transition metal complexes, such as iron-sulfur (Fe-S) complexes. As shown later, early BS computations performed [25,26,27,28,29,30] elucidated fundamental concepts such as oxyl-radical character and several chemical indices to elucidate and understand the nature of chemical bonds of high-valent M=O and MOO species. The chemical indices, such as effective bond order and oxyl-radical character, are indeed useful and effective for current theoretical investigations of oxygenation reactions by P450 and related enzymes. The chemical indices [25] are also obtained with the multi-configuration (MC) models [25,98,99,100,101,102,103,104], such as CASSCF, CASPT2, and RASPT2, providing effective bridges between BS and MC models for metalloenzymes [25,82]. Both BS and MC QM methods have been used for the QM/MM methods, for which MM models are employed for the inclusion of protein fields of metalloenzymes [82,105,106,107,108,109]. 

### 2.2. Isolobal and Isospin Analogy between Organic and Inorganic Peroxides

Fifty years have passed since our proposal of BS approaches [110,111] to organic radical reactions [112]. In the early 1970, we investigated chemical reactions by singlet molecular oxygen [81] and the related organic peroxides, such as carbonyl oxides (CH_2_OO) [82,95,98] with the eight diradical states in the narrow energy region via the BS and UNO CI methods [25,99]; therefore, these molecules are regarded as quasi-degenerated electronic systems [32]. One of the fundamental problems in the theoretical chemistry at that time was *“How to understand and explain the structure and reactivity of iron oxides compounds in* Figure 1?” [42,45,49,50,51,52,53,54,55,56,57,58,59,60,61,62,63,64]. 

In the early 1980s, the reaction cycle for the mono-oxygenation reaction in Figure 1 was not established yet [1,2,3,4,5,6,7,8,9,10,11,12,13,14,15,16,17,18,19,20,21]. Spectroscopic studies for peroxidase have been performed to elucidate possible intermediates, elucidating the Fe(IV)=O π-cation radical, the compound I [13,14,37,38,39,40,41,43,44]. On the other hand, Groves and collaborators [42,45,48,49,50,51,52,53,54] have performed pioneering works for the elucidation of the structure and reactivity of heme-type iron-oxo (Fe=O) model compounds, proposing the rebound mechanism for mono-oxygenations of alkanes by P450. Their early proposal for the active catalysts was the Fe(V)=O species [45,48]. The observed radical reactivity of iron-oxo and iron-peroxide indicated a similarity to the complex oxygenation reaction of the organic peroxide compounds investigated by the BS computations [81,82]. Therefore, the first step of our theoretical approach to the high-valent transition metal oxides was to consider the isolobal and isospin analogy between organic peroxides (CH_2_OO, etc.) and inorganic active peroxides (MOO) on the basis of the BS models [25,26,29,81,82].

**Figure 2:**
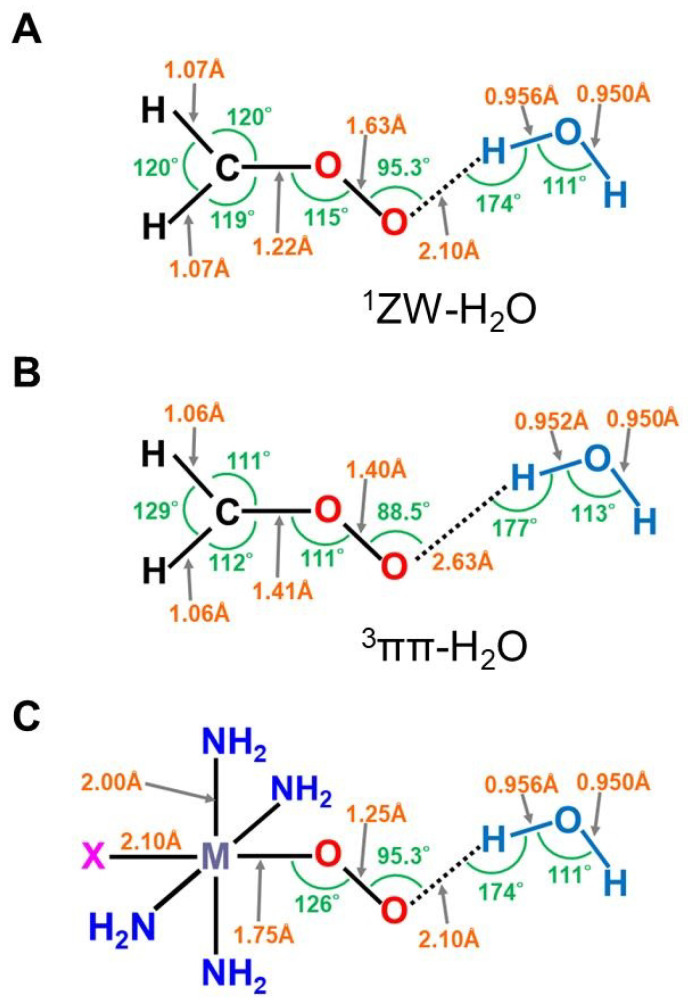
The optimized geometrical structures of the H_2_C-O-O…H_2_O cluster in the singlet (**A**) and triplet (**B**) states and (**C**) computational model for transition metal peroxides (M-O-O) with axial (X) (X = SH^−^, NH_3_) and distal (H_2_O) ligands. The optimized C-O and O-O distances were 1.215 (1.406) and 1.626 (1.402) Å, respectively, for the (**A,B**) structures. The O…H hydrogen bonding distances (O…H-O-H) were 2.098 (2.629) Å, respectively. The (NH_2_)_4_ cluster was employed as a model of porphyrin (Por) in early ab initio computations [25,26,29,82]. The hydrogen-bonding structure for the singlet (**A**) state was assumed for X-(NH_2_)_4_M-O-O---H-O-H (**C**).

The iron-peroxide intermediate **D** in Figure 1 [1,2,3,4,5,6,7,8,9,10,11,12,13,14,15,16,17,18,19,20,21] is an important precursor for the generation of the iron-oxo intermediate **F** for the mono-oxidations. Therefore, theoretical investigations of the nature of the Fe-O-O bonds have been crucial over the past decades. As mentioned above, we have considered the isolobal and isospin analogy between carbonyl oxides (R_1_R_2_C-O-O) [29,113,114] and iron-peroxides (LFe-O-O (L = R_1_R_2_)) [25,26,29,115,116], where R_1_ and R_2_ are substituents for carbonyl oxide and L denotes ligands (heme for heme iron systems) [1,2,3,4,5,6,7,8,9,10,11,12,13,14,15,16,17,18,19,20,21,37,38,39,40,41,45,87]. In fact, we investigated electronic structures of oxygenated dipoles such as carbonyl oxide R_1_R_2_COO that exhibited a chameleonic behavior between the following two extreme structures: 1,3-dipolar structure, R_1_R_2_^+^C=O-O^−^ and/or 1,3-diradical structure, R_1_R_2_•C-O-O•, depending on types of substituents R_1_ and R_2_ and environmental conditions [29,82]. Figure 2 illustrates the optimized structures of the singlet (A) and triplet (B) states of the H_2_C-O-O…H_2_O cluster. The electronic structures (A) and (B) are qualitatively responding to ionic and radical states, respectively. Similar complex behaviors were also expected for LFe-O-O with different axial ligands (L), as illustrated in Figure 2 [26,29,82].

The dissociation of 1,3-dipole; R_1_R_2_^+^C=O-O^−^ was considered to be feasible into the R_1_R_2_C^+^•↑ (doublet fragment *S* = 1/2) and superoxide anion [↓•O-O^−^…H-O-H] (doublet fragment *S* = −1/2) in a solution phase if substituents R_1_ and R_2_ were electron-donating groups [26,29,82]. On the other hand, 1,3-diradical: R_1_R_2_↑•C-O-O•↓ was considered to dissociate into R_1_R_2_C: ↑↑ (triplet fragment *S* = 2/2) and molecular oxygen ↓•O-O•↓ (triplet fragment *S* = −2/2) if substituents R_1_ and R_2_ were not electron-donating groups [26,29,82]. Therefore, BS models have provided the isolobal and isospin analogy between R_1_R_2_^+^C=O-O^−^ and L_1_L_2_Fe(III)=O-O^–^ [26,29,82] and the same analogy between for R_1_R_2_↑•C-O-O•↓ and L_1_L_2_↑•Fe(II)-O–O•↓. The L_1_L_2_↑•Fe(II)-O-O•↓ exchange-coupled structure was considered to be responding to the radical reactivity [16,82], as shown in Figure 2. Recently, this iron peroxide radical is often assumed for oxygenation reactions by P450 [21] and dioxygenase [116]. 

Hydrogen bonding interactions, together with solvation, also play important roles for the conversion from a 1,3-singlet diradical structure to a 1,3-dipole structure, as shown in A and B of Figure 2 [26,29]:

R_1_R_2_↑•C–O–O•↓ + H–O–R → R_1_R_2_^+^C=O–O^−^…H–O–R (6)

The mechanisms of oxygenation reactions by the carbonyl oxides R_1_R_2_COO and the related oxygenated 1,3-dipoles were not simple because of the sensitivity of their electronic and spin structures to environmental effects as elucidated experimentally [113,114], suggesting a similar chameleonic reactivity of transition metal peroxides PorLFe(III)OO [26,29,82,115]. Distal histidine also plays an important role for the hydrogen bonding stabilization of iron peroxides [61].

PorLFeOO + H–N (His) → PorLFe(III)-O–O^−^…H–N (His) (7)

The isolobal and isospin analogy between [R_1_R_2_C-O-O…H-O-H] and [LFe-O-O…H-N(His)] indeed provided a prediction that carbonyl oxide models were applicable to transition metal peroxides species in Figure 2 [26,29,82,113,114,115]. Quantum mechanically, the electronic structures of these species are expressed by the superposition of the zwitterionic (ZW) and diradical (DR) extreme structures and their weights are variable with the types of ligands and environments. For example, the common moderate 1,3-diradical character (about 40~50%, *Y* = 0.4~0.5) (see the next section) was concluded for both carbonyl oxide H_2_COO and iron-peroxide FeOO [26,29,82]. Therefore, FeOO with coordinated ligand (L) was regarded as a coupling structure between LFe(III)^+^↑ (*S* = 1/2) and superoxide anion (^−^O–O•↓) (*S* = −1/2) if L was a strong electron-donating ligand such as cysteine anion ^–^SR in cytochrome P450 (L = ^−^SR) in **D** of Figure 1 [1,2,3,4,5,6,7,8,9,10,11,12,13,14,15,16,17,18,19,20,21,37,38,39,40,41]. The same picture is feasible for chloroperoxidase with L = thiolate anion, peroxidase with L = imidazole anion, and catalase with L = phenolate anion. On the other hand, LFeOO was regarded as an exchange coupling state between LFe(II)↑↑•• (*S* = 2/2) and triplet molecular oxygen (↓•O–O•↓) (*S* = −2/2) under the condition of the weak electron-donating ability of L [26,37,38,39,40,41]. Therefore, the latter picture was applicable to molecular oxygen transfer enzymes such as Mb and Hb with L = histidine [83].

**Figure 3:**
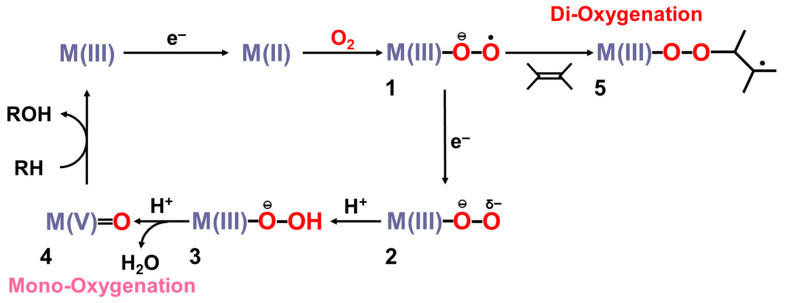
Formations of transition metal superperoxide (**1**), oxygen dianion (**2**), metal hydroperoxide (**3**) and metal-oxo (**4**) intermediates, and radical addition intermediate (**5**) for oxygenation reactions [26,27,29,81,82]. **1** and **4** were assumed for active transition metal (M) peroxo and oxo catalysts for dioxygenase [22,23,24] and monooxygenase [21,83,84,85,86,87,88,89,90], respectively.

The FeOO species undergoes O–O bond fission in the cytochrome P450 enzyme and peroxidase, as shown in Figure 1; Figure 3 [1,2,3,4,5,6,7,8,9,10,11,12,13,14,15,16,17,18,19,20,21,37,38,39,40,41]. The FeOO species formed by one electron capture in **E** is nucleophilic because of the O_2_ dianion character. Therefore, the formation of LFe(III)-hydroperoxide is feasible by the proton addition to PorLFeOO, as shown in the steps **E** and **E’** [26,82].

L–Fe(III)^+^–^−^O–O• + e^−^ → L–Fe(III)^+^−^−^O–O^−^ + H^+^ → L–Fe(III)–^−^OOH (8)

The heterolysis of hydroperoxide bond is further feasible with the addition of one more protons in the next step from **E’** to **F** as follows [1,2,3,4,5,6,7,8,9,10,11,12,13,14,15,16,17,18,19,20,21,37,38,39,40,41].

L–Fe(III)–OOH + H^+^ → L–Fe(III)–O^+^…^−^OH...H^+^ → L–Fe(V)=O + H_2_O (9)

The formal Fe(V)=O bond in **4** of Figure 3 has been extensively investigated as the active site of the mono-oxygenation reaction by cytochrome P450 enzyme [1,2,3,4,5,6,7,8,9,10,11,12,13,14,15,16,17,18,19,20,21,37,38,39,40,41,42,43,44,45]. However, the high-valent porphyrin (Por)LFe(V)=O complex was often converted into π cation radical Por(^+^•)LFe(IV)=O intermediate if L was taken as a strong electron donor, such as histidine anion of peroxidase, cysteine anion (^−^SR) of cytochrome P450 and chloroperoxidase (CPO), and phenolate anion (Phe-O^−^) of catalase. The Por(^+^•)LFe(IV)=O is often referred to as the cytochrome P450 compound I (Cpd I) [49,50,51,52,53,54,55,56,57,58,59,60,61,62,63,64]. 

The homolysis of the hydroperoxide [21,26,82] was proposed in some cases (L = Cl^−^) as follows:

L–Fe(III)–OOH → L–Fe(III)–O•…•OH...H^+^ → L–Fe(IV)=O + •OH (10)

The hydroxyl radical was often toxic in biological systems [21,82]. It was generated in the shunt pathway using hydroperoxide (H_2_O_2_) [47,55], as shown in Figure 1. Therefore, constructions of appropriate ligand fields (L) are required to suppress the hemolysis. Por-L-Fe(IV)=O was referred to as the compound II (Cpd II). Cpd II with the heme-ligand was often a sluggish reagent like triplet molecular oxygen (^3^O-O) in sharp contrast to atomic oxygen (^3^O) [26,27,81]. Therefore, the ^3^O model was proposed for reactive Cpd I [81]. The atomic oxygen model has been applied to non-heme Fe(IV)=O species, as discussed later. Thus, the isolobal and isospin analogy between R_1_R_2_C-O-O and LFe-O-O provided qualitative pictures of the iron-peroxide intermediates [26,29,81,82]. The chemical reactivity of the LFe-O-O in **1** of Figure 3 was hardly elucidated in relation to the di-oxygenation reactions [22,23,24] at that time. A recent XRD study has elucidated the structure of the reaction site of the 2,3-dioxygenase catalyzing the cleavage of the pyrrole ring of tryptophan, suggesting the decomposition pathway through dioxetane (see the Section 4.2) [116]. 

Molecular oxygen (O=O) with the orthogonal π* orbitals is a magnetic molecule with the triplet ground state. Atomic oxygen (^3^O) in the ground triplet state exhibits radical reactivity. On the other hand, singlet atomic oxygen (^1^O) undergoes the insertion reaction. The high-valent Fe(V)=O species generated in the **F** step is a doublet species with the electrophilic LUMO like ^1^O [81]. On the other hand, the Fe(IV)=O species in Cpd I is a ground triplet species with radical reactivity. Therefore, we have proposed the ^1^O and ^3^O models for stereospecific and non-stereospecific epoxidation of the C=C double bonds [81]. The isolobal and isospin analogy among Fe(IV)=O, O=O, and O [26,27,81,82] was indeed feasible for the theoretical understanding of the mono-oxygenation reactions by P450 [1,2,3,4,5,6,7,8,9,10,11,12,13,14,15,16,17,18,19,20,21] and related non-heme transition metal oxo compounds [37,38,39,40,41,42,43,44,45,46,47,83,84,85,86,87,88,89], as shown later. 

Newcomb and his collaborators [65,66,67,68,69,70] have investigated the stereochemistry of mono-oxygenations of heme-Fe(IV)=O species using hypersensitive radical probes, indicating that the radical rebound mechanism is not complete for the P450-catalyzed hydroxylations. They have proposed an ionic mechanism (+OH insertion model) instead of the radical mechanism. Thus, multiple oxidant models have been proposed for mono-oxygenation reactions by P450 model complexes [65,66,67,68,69,70]. To this end, mutation experiments of the native P450 have been also performed [21]. The isolobal and isospin analogy among M=O (M = Cr, Mn, Fe), O=O, and O revealed by the BS computations [26,27,29,30] have provided a fundamental concept for the understanding of chameleonic mono-oxygenation reactions by P450 and related metal-oxo compounds [42,43,44,45,46,47,48,49,50,51,52,53,54,55,56,57,58,59,60,61,62,63,64,65,66,67,68,69,70]. The multiple oxidant models have been also applicable for the high-valent Mn-oxo compounds [71,72,73,74,75,76,77], indicating the necessity of theoretical investigations of the nature of 3d transition metal oxo compounds [27,81]. The BS models with both charge and spin degrees of freedom have been handy and practical for theoretical investigations of complex ionic and radical intermediates in the multiple intermediates model for P450 and related systems in Figure 1, Figure 2 and Figure 3. 

## 3. Ab Initio Calculations of Metal Oxo and Peroxide Complexes

### 3.1. Ab Initio Calculations of Metal Peroxide Complexes

In this section, early ab initio computations for the elucidation of the nature of the metal-peroxide (MOO) bonds have been revisited [26,29,82]. In the 1980s, we performed ab initio BS(UHF) computations of several model complexes R_1_R_2_LMOO complexes for Por-L-MO_1_O_2_ complexes (M = Cr, Fe, Ni; R_1_R_2_ = (NH_2_)_4_ for Por; L = NH_3_, ^−^SR) in order to confirm the guiding principles based on the above-mentioned isolobal and isospin analogy [26,29,82]. Figure 2 illustrates the computational model for the transition metal peroxides with a distal water molecule used as a model of distal histidine. The (NH_2_)_4_ model was employed for a computational economy although the porphyrin ligand was employed for the EHMO and *Xα* calculations [96,97]. The (NH_2_)_4_ model is now regarded as a model of tetradentate ligands of non-heme Fe(IV)=O species (see later). The simple theoretical model enabled us to construct many spin configurations for these complexes, even at that time. Some of the early computational results are summarized in Table 1. 

From Table 1, the net charge populations on the O_1_ and O_2_ sites were −0.3~−0.4 and 0.5~0.6, respectively, for naked M(III)OO (M = Cr, Fe), indicating electrophilic property of the naked systems. On the other hand, the corresponding values were −0.33~−0.35 and −0.01~0.10, respectively, for the model complexes (NH_2_)_4_Fe(III)OO, (NH_2_)_4_NH_3_Fe(III)OO, and (NH_2_)_4_NH_3_Fe(III)OO…H-O-H (see Figure 2). The total negative electron density of the OO part was only −0.3~−0.4 in accordance with weak superoxide character. Therefore, these model complexes are regarded as models for oxygen carrier complexes such as Mb and Hb [20,83]. On the other hand, the corresponding charges were −0.53~−0.65 and −0.44~−0.63, respectively, for the model complexes (NH_2_)_4_(SH^−^)Fe(III)OO and (NH_2_)_4_(SH^−^)Fe(III)OO…H-O-H. The total negative charges of the OO part were about −1.0 in the case of axial thiolate anion ligand, indicating the strong superoxide character [21]. In the late 1980s, a superoxide radical model was proposed for dioxygenase based on these computational results [29,82], as shown in Figure 3. Recently, L-FeOO intermediates have often been assumed for the explanation of the oxygenation reactions [21,116].

**Figure 4:**
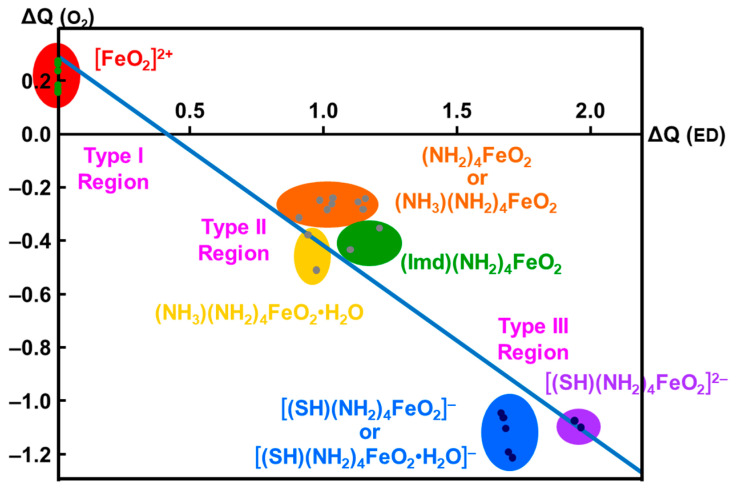
Variations of the superoxide (SOD) character (ΔQ(O_2_) for transition metal peroxide complexes against reduction of net negative charges (ΔQ(ED)) on the electron-donating (ED) ligand [26,29,82]. The electronic properties of the iron-peroxide bonds are classified into three type I-III on the basis of the BS(UHF) computations.

The net charges ΔQ(O_2_) on the OO part for the transition metal (Fe) peroxides in Table 1 were found to be parallel to the electron-donating ability of ΔQ(ED) of ligands (NH_2_)_4_L, which was defined as the net electron transfer from ligands to the native core Fe(II)O_2_ [29,82]. Figure 4 clearly indicated the linear relationship between ΔQ(O_2_) and ΔQ(ED) as follows:

ΔQ(O_2_) = −0.73 ΔQ(ED) + 0.29 (11)

From Figure 4, the BS computational results have elucidated that the transition metal peroxides are classified into the following three types: (1) Type I with the weak superoxide (SOD) character (0.0 < ΔQ(ED) < 0.5), (2) Type II with the intermediate SOD character (0.5 < ΔQ(ED) < 1.5), and Type III with the strong SOD character (1.5 < ΔQ(ED)) [26,29]. The molecular oxygen (O_2_) is a weak electron donor for the naked Fe(II) ion, which is classified into Type I. On the other hand, the O_2_ site is a one electron acceptor for iron-peroxide complexes Fe(III)-(OO^−^•) with strong electron-donating ligands, particularly the thiolate anion group in accordance with Type III. The ab initio computational results have been wholly compatible with the characteristic properties of PorLFeOO cores of metalloenzymes examined in the preceding section II. The superoxide anion in the intermediate **E** of Figure 1 accepts one electron to afford the oxygen dianion followed by protonation to afford hydroperoxide, as shown in **E**’ of Figure 1. Thus, early BS computations [26,29,82] provided important information for the theoretical understanding of the nature of the chemical bonds of the metal peroxides (MOO).

### 3.2. Ab Initio Calculations of Metal Oxo Complexes

In this section, early ab initio computations for the elucidation of the nature of the metal-oxo (M=O) bonds have been revisited [27]. The ab initio UHF computations of transition metal oxo (M=O) species were performed to elucidate the electronic and spin structures [27,81]. Some of the computational results for the naked cores M=O are summarized in Table 2 [27]. From Table 2, the electronic configuration of the neutral [Fe(II)=O]^0^ core (3d^6^-2p^4^ model) is given by Equation (12). The one σ and twoπ bonds were doubly occupied for the naked core [Fe(II)=O]^0^,

(*dδ_xy_*)^X^ (*dδ_x_*_2−*y*2_)^Y^ (*dσ-pσ*)^2^ (dσ-pσ*)^0^ (*dπ_xz_-pπ_xz_*)^2^ (dπ_xz_-pπ_xz_*)^1^ (*dπ_yz_-pπ_yz_*)^2^ (dπ_yz_-pπ_yz_*)^1^ (12a)

whereas the antibonding σ* orbital was vacant. The two antibonding π* orbitals and two δ orbitals (X = Y = 1) were singly occupied, providing the quintet ground state (*S* = 4/2). Theoretically, the σ-σ* spin-flip excitation was feasible to afford the septet excited state (*S* = 6/2). The σ-σ* excitation energy was calculated to be about 13 kcal/mol, indicating an important contribution to the ferromagnetic exchange interactions among 3d electrons. The one-electron oxidation in the *dδ*_*x*2−*y*2_ orbital (X = 1 and Y = 0 in Equation (12)) of Fe(II)=O provided [Fe(III)=O]^1+^ with the quartet (*S* = 3/2) ground and sextet (*S* = 5/2) excited states. The σ-σ* excitation energy was calculated to be about 18 kcal/mol. The two-electron oxidation of the δ-orbital provided the [Fe(IV)=O]^2+^ with the triplet iron-oxo (*S* = 2/2) core. The energy gap between the ground triplet and excited singlet states was calculated to be about 27 kcal/mol. 

The above three Fe=O core models have the common triplet iron-oxo core with different occupation numbers (X and Y) of δ-orbitals. Therefore, the net charges on the oxygen site were calculated to be −0.72, −0.55, and 0.35 for the following three cases: X = Y = 1, X = 1 and Y = 0, and X = Y = 0, indicating variations from the nucleophilic to the electrophilic property, depending on the occupation numbers of δ-orbitals [27,81]. This means that the [Fe(IV)=O]^2+^ core embedded in porphyrin dianion becomes nucleophilic since the occupation numbers are X = 2 and Y = 0 (low spin) for [PorFe(IV)=O]^0^. Therefore, the one-electron oxidation is necessary for the generation of the electrophilic heme iron compounds; [PorFe(V)=O]^1+^ ↔ [Por(+)Fe(IV)=O]^1+^. On the other hand, the non-heme complexes, [L_1_L_2_Fe(IV)=O]^Z+^ are often total quintet state (*S* = 4/2) because X = Y = 1 in Equation (12) (see details in Figure 1 and Table 2). 

**Figure 5:**
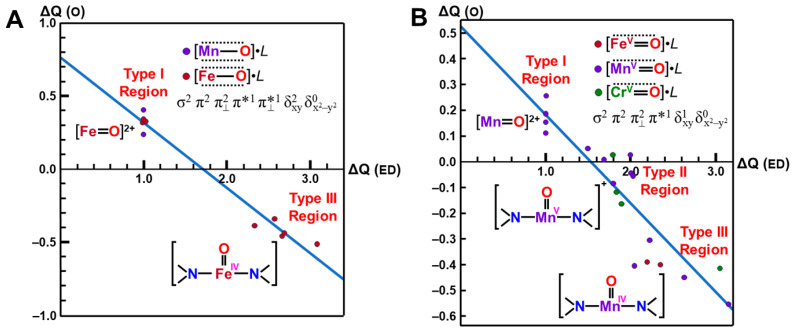
Variations of the electrophilic character (ΔQ(O) for transition metal oxo complexes against reduction of net negative charges (ΔQ(ED)) on the electron-donating (ED) ligand in (**A**) Fe(IV)=O and (**B**) Mn(X)=O (X = IV, V) with (NH_2_)_2_ porphyrin model [27,81]. The transition metal oxo bonds are also classified into Types I, II, and III on the basis of the BS(UHF) computations.

In order to elucidate the ligand coordination effects, we examined the {[Fe(IV)O]^2+^(NH_2_)_2_}^0^ complex, as shown in Figure 5A [27,81]. The net charges ΔQ(O) on the O part for the transition metal (M) oxo compounds in Table 2 were found to be parallel to the electron-donating ability of ΔQ(ED) of ligands, which is defined as the net electron transfer from ligands to the native cores [M(IV)O]^2+^ (3d^6^-2p^6^ model). Figure 5A clearly indicates the linear relationship between ΔQ(O_2_) and ΔQ(ED) for {[Fe(IV)O]^2+^(NH_2_)_2_}^0^ under the low-spin condition (X = 2 and Y = 0) as follows:

ΔQ(O) = −0.44 ΔQ(ED) + 0.76(13a)

From Figure 5A, the net charges on the oxygen site were calculated to be 0.76 and 0.32 for ΔQ(ED)=0 and 1, respectively. The corresponding values for the naked core Fe(III)O]^+^ by ab initio methods were 0.48~0.67, depending on the orbital and spin configurations in Equation (12), as shown in Table 2, supporting the estimation formula in Equation (13a). The net charge of the O-site was estimated to be 1.20 for ΔQ(ED) = −1 in Equation (13a). The oxygen site was an electron donor for the Fe(X) (X = IV, V) ion, indicating the electrophilic reactivity of the oxygen site towards electron-donating substrates, such as PPh_3_ [27]. 

On the other hand, the negative charge on the O-site was about −0.5 for the total neutral complex model [Fe(IV)O(NH_2_)_2_]^0^ in the strong electron-donating ligands (2.5 < ΔQ(ED)), as shown in Figure 5A. Therefore, the oxygen site of iron-oxo compounds is nucleophilic in this region [27], indicating that one more oxidation of the Fe ion is necessary for the conversion from nucleophilic to electrophilic in nature. Indeed, the net charge for the oxygen site of [Fe(V)O(NH_2_)_2_]^1+^ is estimated to be about zero in the region (1.5 < ΔQ(ED) <2.0), indicating the electrophilic or radical (see later) reactivity. Thus, the computational results elucidated variations of the reaction modes of iron-oxo species, depending on electron-donating properties of ligands and valence state of Fe ion.

For comparison with the high-valent iron-oxo species, ab initio computations [27] were also performed for [Mn(IV)O(NH_2_)_2_]^0^ complex, as shown in Figure 5B. Figure 5B also indicated the linear relationship between ΔQ(O_2_) and ΔQ(ED) for {[Mn(IV)O]^2+^(NH_2_)_2_}^0^ under the low-spin δ structure (X = 1 and Y = 0) as follows:

ΔQ(O) = −0.34 ΔQ(ED) + 0.53(13b)

From Figure 5B, the net charges on the oxygen site were calculated to be 0.87, 0.53, and 0.19 for ΔQ(ED) = −1, 0, and 1, respectively. The electron-accepting ability of Mn(IV) is slightly lower than that of the Fe(IV) ion in accordance with the 3d-orbital level by the extended Hubbard model [25]. From Table 2, the net charges of the O-site by the ab initio calculations were 0.18 and 1.08 for the naked [Mn(IV)O]^2+^ and [Mn(V)O]^+^. The oxygen site was an electron donor for the naked Mn(IV) ion, indicating the electrophilic reactivity towards electron-donating substrates. 

On the other hand, the negative charge on the O-site was about −0.5 for the total neutral complex model [Mn(IV)O(NH_2_)_2_]^0^ in the strong electron-donating ligands (2.5 < ΔQ(ED)), as shown in Figure 5B. Therefore, the oxygen site of manganese-oxo compounds was nucleophilic in this region. The net charge for the oxygen site of [Mn(V)O(NH_2_)_2_]^1+^ was also estimated to be about zero in the region (1.5 < ΔQ(ED) < 2.0). Thus, ab initio computations elucidated that the nature of high-valent Fe(X)=O and Mn(X)=O (X = IV, V) bonds exhibit chameleonic behavior, depending on the electron-donating ability of the coordinated ligands and valence states of M (=Fe, Mn) ions, indicating the necessity of careful QM/MM modeling of the catalytic sites for mono-oxygenations. The BS extended Hubbard model (UEHB) [25], UHF [27], *Xα* [96,97], and Hartree–Fock–Slater (UHFS) models [25] were useful for qualitative understanding and explanation of electronic and spin states of the high-valent metal-oxo compounds. The UEHB model [25] was used to reduce the large on-site repulsion integral (*U*) by UHF since the UHF coupled cluster (CC) SD(T) model for the reduction of *U* was too heavy at that time. Nowadays, the reduction of U was alternately accomplished with hybrid DFT (HDFT) [117,118,119] models for M=O and MOO after the calibrations based on the UCCSD(T) results.

### 3.3. Comparisons among UHF, UCCSD(T), and Hybrid DFT Computational Results

Here, geometric structures and binding energies of the metal oxo bonds by several computational methods have been revisited. BS UHF model was a first step toward theoretical elucidation of the nature of chemical bonds of the transition metal oxo compounds [25,26,27]. As a next step, we examined the unrestricted CC (UCC) SD(T) model, including dynamical correlation corrections [25,32,78,79]. The hybrid UHF plus UDFT (HUDFT) [117,118,119] was also examined as an alternative tool of UCCSD(T) for handy and practical investigations of large metal-oxo compounds. Therefore, UHF, UCCSD(T), and HDFT (UB3LYP and UBLYP) computations were performed to elucidate the optimized M-O lengths of the naked neutral metal oxo species [78,79], for which the observed bond lengths were available, as shown in Table 3. Two different basis sets, (1) double zeta (DZ) basis (BS I) and (2) triple zeta (TZ) basis (BS II), were used for elucidation of the basis set dependency, as shown in Table 3.

From Table 3, the calculated M-O bond lengths were not so different between the basis sets (1) and (2) [78,79]. The M-O bond lengths by UHF were longer by about 0.2 Å than the corresponding experimental values. On the other hand, the UCCSD(T) computations exhibited remarkable improvements of the calculated M-O lengths in accordance with the experimental results, as shown in Table 3. Both hybrid UDFT (UB3LYP and UBLYP) also provided reasonable M-O bond lengths as compared with the experiments. Thus, HUDFT was reliable enough for geometry optimizations of the M-O bond lengths [78,79].

The UHF, UCCSD(T), UB3YP, and UBLYP computations were performed to elucidate the binding energies of the naked neutral metal oxo species, for which the observed bond lengths were available, as shown in Table 4 [78,79]. From Table 4, the binding energies of the M=O bonds were highly dependent on the basis sets, indicating that the larger TZ basis set (BS II) is more favorable than the DZ basis set (BS I). The calculated binding energies for M=O by UCCSD(T)/BS II were smaller than the observed values. This implies that more larger basis sets than the BS II are necessary for quantitative UCCSD(T) computations of the binding energies of the M=O species; moreover, more expensive CC methods, such as UCCSDT, may be necessary for quantitative purpose. On the other hand, UBLYP methods/BS II overestimated the binding energies of M=O because of underestimation of the electron–electron repulsion (*U*) effects. Thus, UB3LYP/BS II model was found to be practical for the theoretical investigation of M=O species at that time [78,79].

UB3LYP/BS II methods indeed provided reasonable binding energies of M=O as compared with the experimental values, as shown in Table 3 [78,79]. The calculated binding energies by the methods were 4.34 (4.57), 4.09 (3.83), 4.38 (4.17), 4.07 (3.94), 4.13 (3.91), and 2.69 (2.75) eV for CrO(^5^Π), MnO(^6^Σ^+^), FeO(^5^Δ), CoO(^4^Δ), NiO(^3^Σ^−^), and CuO(^2^Π) in the ground state, respectively, where the experimental values are given in parentheses. The relative energies among several spin states of each MO species were obtained by changing the occupation numbers of eight molecular orbitals in Equation (12). For example, the binding energies for FeO were 4.38, 3.36, 3.92, and 3.40 eV for the ^5^Δ, ^3^Φ, ^5^Σ^+^, and ^7^Σ^+^ states, respectively, indicating four states within the narrow energy region (about 1 eV). The physical terminology *“quasi-degenerated energy states”* [32] is often used for 3d metal-oxo species in Table 3. Therefore, theoretical investigations by UHDFT, such as UB3LYP, were found to be contributable to the elucidation of the complex reaction mechanisms of the iron-oxo bonds [78,79]. Indeed, UB3LYP have been employed for many theoretical investigations of chemical reactions by P450 [83,84,85,86,87,88,89,90] (see later).

## 4. Orbital Bifurcations for Radical Reactions and Derivations of Chemical Indices

### 4.1. HOMO-LUMO Mixing for Homolytic and Electron-Transfer Diradicals by BS Models

**Figure 6:**
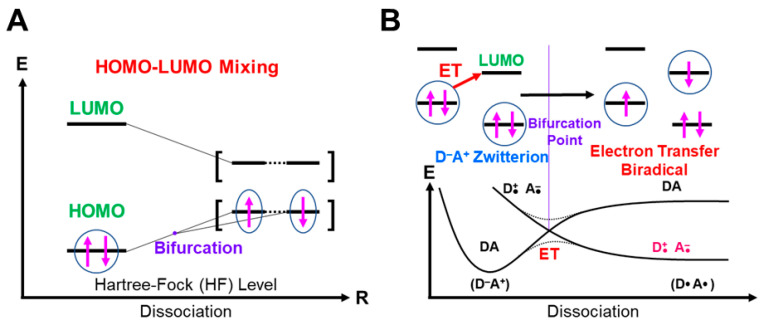
HOMO-LUMO mixing for homolytic diradical [93] (**A**) and one-electron transfer (OET) (**B**) diradicals [94] under the BS theoretical approximation. The singlet closed-shell pair bifurcates into the open-shell singlet diradical pair through the bifurcation point. The up and down arrows are denoted the up and down spins of electron, respectively.

**Figure 7:**
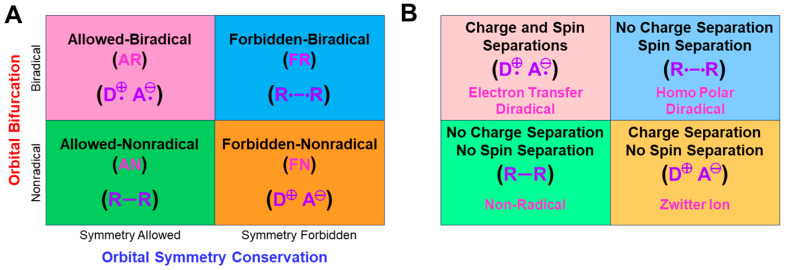
(**A**) Classifications of reaction mechanisms on the basis of the orbital symmetry conservation and orbital bifurcation into four groups and (**B**) characterization of them by populations of the charge and spin densities obtained by the BS computations [25,27,112].

In this section, the intra- and inter-HOMO-LUMO mixings are revisited for MO-theoretical elucidation of homolytic and electron-transfer diradicals [25,26,27,28,29,30]. In the 1980s, the high-valent transition metal oxo M(X)=O (X = IV, V) compounds were found to exhibit electrophilic and/or radical reactivity for alkanes, etc. [42,43,44,45,46,47,48,49,50,51,52,53,54,55,56,57,58,59,60,61,62,63,64,65,66,67,68,69,70,71,72,73,74,75,76,77] in contradiction to the nucleophilic reactivity of low-valent M(X)=O (X = II) species. As mentioned above, it was a challenge for theoretical chemists to explain the unusual properties of these species. Here, fundamental concepts and basic theories of the broken-symmetry (BS) approach are re-visited for theoretical elucidations of structure, bonding, and reactivity of organic and inorganic peroxides with non-negligible radical character because of narrow HOMO-LUMO energy gaps, as shown in Figure 6 and Figure 7. To this end, diradical intermediates are formally classified into homolytic [93] and electron-transfer [94] diradicals, as illustrated in Figure 6. 

Anti-aromatic molecule [120] and 1,3-diradicals [121] were typical homolytic diradical species [93,95]. Recently, many diradical compounds have been synthesized and characterized by several spectroscopic methods [122]. The orbital bifurcation of a doubly occupied covalent bond into spin-polarized (SP) radical orbitals occurs under the mathematical condition where the HOMO-LUMO energy gap given by Δ*ε* = *ε*_LUMO_ − *ε*_HOMO_ is smaller than the on-site electron repulsion integral (*U*) in the BS approximation [25,26,27,28,29,30,78,79,81,82]

(14) $$\Delta \varepsilon =\varepsilon_{\mathrm{LUMO}}-\varepsilon_{\mathrm{HOMO}}/U<1$$

where the *ε_X_* (*X* = HOMO, LUMO) denotes the orbital energy of *X*. For example, such situations occur in the dissociation reactions of hydroperoxide and related peroxides (for example metal-hydroperoxide [82]), as shown in Figure 6A [25,26].

H-O-O-H → [H-Oδ•…•δO-H] → HO• + •OH(15a)

M-O-O-H → [M-Oδ•…•δO-H] → M-O• + •OH(15b)

Under the BS model, the HOMO-LUMO mixing procedure is introduced for construction of the UHF [93] and UHDFT solutions [117,118,119]. The resulting MOs at the UHF and UHDFT levels of theory are given by the HOMO-LUMO mixing by the restricted (R) Hartree–Fock (RHF) and R-HDFT (RHDFT) solutions [25,93,119], as shown in Equation (16). Here, the HOMO-LUMO mixing scheme [25,93,119] is given by

(16a) $$\Psi_{i}^{+}=\cos\theta \varphi_{i}+\sin\theta \varphi_{i}^{*}$$

(16b) $$\Psi_{i}^{-}=\cos\theta \varphi_{i}-\sin\theta \varphi_{i}^{*}$$

where *θ* denotes the orbital mixing-parameter determined by BS computations. Since HOMO, $\varphi_{i}$ and LUMO, $\varphi_{i}^{*}$, by RHF (RHDFT) are symmetry-adapted and usually belong to different spatial symmetries (*P*_n_), the resulting BS MOs,$\;\Psi_{i}^{+}$ and $\Psi_{i}^{-}$, obtained by the HOMO-LUMO mixing [93] are often spatially symmetry-broken in accordance with the naming of the BS method in general. 

In fact, the dπ-pπ bonds of the high-valent M=O species were rather covalent instead of the ionic bonds. Therefore, the HOMO (dπ-pπ)-LUMO (dπ-pπ)* mixing on Equation (16) provided the BS orbitals, which were mainly localized on the M- and O-site, ↑•M-O•↓, respectively. The up and down spins are into BS orbitals, namely, “*different orbitals and different spins (DODS)*” [25,26,27,28,29,30], as illustrated in Figure 6A. Chemically, the unstable intermediate in Equation (15) is often referred to as a homolytic diradical in the homolysis of the O-O bond.

Very strong charge-transfer (CT) complexes often provided an electron transfer diradical as in the case of aromatic radical substitution reaction [123,124,125]. The HOMO-LUMO energy gap becomes small for donor (D) and acceptor (A) pairs, indicating the HOMO (donor; D)-LUMO (acceptor; A) mixing responsible for one-electron transfer (OET) process in Equation (17) [94].

D + A → [Dδ•^+^…^−^δ•A] → [D•^+^ + ^−^•A] (17)

The OET process was formally regarded as a symmetry-allowed radical process since HOMO(D) and LUMO(A) have the same spatial symmetry [94]. Both charge and spin are separated in the OET diradical, as shown in Equation (17). The OET diradical in Equation (18a) is often assumed for proton-transfer reactions for mono-oxygenation reactions, followed by the radical rebound mechanism in Equation (18b). The redox potentials of the reactants play an important for the formation of the OET radical in Equation (18a) [94,123,125]. The hydroxylation via Equation (18a,b) is often referred to as the electron transfer-coupled proton transfer (ET-PT) process [83,84,85,86,87,88,89].

M(X)=O + H-R → [M(X–1)-O^−^…^+^H…•R] → [M(X–1)-OH…•R] (18a)

[M(X–1)-OH…•R] → [M(X–2)-•OH…•R] → [M(X–2)-HOR](18b)

On the other hand, homolytic diradicals formed by the intramolecular HOMO-LUMO mixing undergo hydrogen radical abstractions as follows:

M(X)=O + H-R → [M(X–1)-O•↓…δ↑•H…δ↓•R] → [M(X–1)-OH…↓•R](19)

The radical rebound process in Equation (18b) is also applicable for the mono-oxygenation by the hydrogen radical abstraction (HRA) mechanism [83,84,85,86,87,88,89]. However, second hydrogen abstraction by M(X–2)-•OH in Equation (18b) is feasible to afford olefins, M(X–2)-HOH + R(C=C), in some case. From the reaction scheme in Equation (19), the binding energy of the H-C bond is directly related to activation barriers of the HRA reaction by P450 and related iron-oxo compounds [83,84,85,86,87,88,89,90].

### 4.2. Four Different Mechanisms of Chemical Reactions by BS Models

In this section, the concepts of orbital symmetry and orbital bifurcation have been revisited in relation to electronic mechanisms of chemical reactions [25]. In the 1970s, the concept of orbital symmetry conservation was a guiding principle for understanding, explanation, and prediction of symmetry-allowed concerted reactions [126,127,128]. The Hückel MO (HMO) and extended Hückel molecular orbital (EHMO) models were employed for the elucidation of energy levels and spatial symmetries of Mos for reacting molecules under consideration [126]. The gap of the HOMO-LUMO (frontier orbitals [127]) was usually large for nonradical concerted reactions, as shown in Figure 6A. On the other hand, the HOMO-LUMO energy gaps became small for diradical species, as shown in Equation (14), nevertheless indicating no orbital bifurcation at the EHMO level of theory because the electron repulsion (*U* = 0) was neglected. 

On the other hand, as mentioned above, the orbital bifurcation took place at the Hartree–Fock (HF) MO level of theory, providing DODS Mos for homolytic [93] and electron transfer [94] diradicals, as shown in Figure 6. Therefore, radical reaction mechanisms can be explained with the orbital bifurcations and the resulting *DODO* orbitals. Indeed, the combination of the following two fundamental orbital concepts: “*orbital symmetry conservation and orbital bifurcation*” provided four different mechanisms, as shown in Figure 7A [25,26,27,28,29,30,93,94]. The four mechanisms defined by two criteria have been equally applicable for BS models, such as UHF [25,93] and UHDFT [78,79,119], in general. 

The original orbital symmetry-allowed reaction is characterized as the symmetry-allowed nonradical (AN) reaction [25,112], as shown in Figure 7A. On the other hand, the orbital symmetry-forbidden reaction is regarded as the symmetry-forbidden radical (FR) reaction [25,112]. The formal FR reaction is often converted into the symmetry-forbidden nonradical (FN) reaction under the condition that the HOMO-LUMO gap becomes larger than the on-site repulsion (*U*) (see Equation (10)) because of push-pull effects by substituents introduced. The zwitterionic (ZW) reaction is an example of such FN reactions [113,114]. On the other hand, the HOMO-LUMO gap becomes very small for a donor-acceptor pair, providing a formally symmetry-allowed electron-transfer radical (AR) process [123,124,125] in Figure 7A. Thus, the concepts of the orbital symmetry and orbital bifurcation are useful for the theoretical understanding of chemical reaction mechanisms in general.

The charge and spin density populations at the BS level of theory are also related to four different mechanisms in Figure 7B [25,112]. The AN reactions are characterized by no charge separation and no spin separation, as shown in Figure 7B. On the other hand, the FR reactions are usually characterized by no charge separation, but remarkable separation between up- and down-spins. The spin density disappears in the case of the FN reactions, whereas the separation between the plus and minus charges becomes remarkable, as shown in the ZW intermediate [113,114]. The AR reactions are characterized by both charge and spin separations, as in the case of electron-transfer diradicals. Thus, the populations of both the charge and spin densities by the BS models are useful for classifications of the four chemical reaction mechanisms, as shown in Figure 7B. 

### 4.3. Derivations of Effective Bond Orders for Fe(IV)=O and Fe(V)=O Bonds 

In this section, derivations of several chemical indices have been revisited for the characterization of the nature of the chemical bonds of the metal-oxo bonds [25,27,29]. Theoretical computations are often regarded as convenient and practical tools to calculate potential curves and activation barriers for chemical reactions. The computational results further provide important information to obtain deep insights into the quantum mechanisms of chemical reactions. Populations of charge and spin densities by BS models were practical and convenient indices for characterizations of reaction mechanisms, as shown in Figure 7. On the other hand, the concept of the symmetry and/or orbital phase of molecular orbitals plays important roles in the Woodward–Hoffmann–Fukui rule for concerted reactions [126,127]. However, the HOMO-LUMO mixing occurs in the BS approach for radical reactions [93], indicating the orbital bifurcation. Therefore, we performed the natural orbital analysis of the BS solutions to elucidate the natural orbitals (UNO) and their symmetries and occupation numbers [25,32], which were crucial for MO theoretical understanding and explanations of electronic and spin structures of diradical and polyradical species [25,93,94]. To this end, several chemical indices were also derived to investigate the nature of chemical bonds of open-shell systems [25,128]. The localized natural orbitals of UNO (ULO) were also used to obtain the VB-like models [25] of open-shell molecules (see Appendix A). 

The orbital overlap *T_i_* between BS MOs obtained by the HOMO-LUMO mixing in Equation (16) was defined as an order parameter to elucidate the magnitude of the orbital bifurcation [25,93].

(20) $$T_{i}=\langle \Psi_{i}^{+}|\Psi_{i}^{-}\rangle =\cos2\theta$$

Therefore, the *T_i_* index becomes 1.0 in the case of the closed-shell (restricted) case; $\Psi_{i}^{+}$ = $\Psi_{i}^{-}$ = $\varphi_{i}$, whereas *T_i_* is 0.0 for the complete mixing case (*θ* = π/4): complete diradical pair with 100% diradical character (*Y* = 1.0, see Equation (27a)). The effective bond order b was defined as an extension of the Coulson’s bond order based on the MO model by [25,93,129] to express the decrease in chemical bonding via the intramolecular HOMO-LUMO mixing,

(21a) $$b_{i}=\frac{n_{i}-n_{i}^{*}}{2}=\frac{\left(1+\cos2\theta \right)-\left(1-\cos2\theta \right)}{2}$$

(21b) $$=\cos2\theta =T_{i}$$

where *n*_i_ (= 1 + *T_i_*) and *n*_i_* (= 1 − *T_i_*) denote the occupation numbers of the bonding (HOMO) and antibonding (LUMO) orbitals, respectively. The effective bond order (*b*) is nothing but the orbital overlap (*T_i_*) between BS MOs under the BS approximations [25,93]. 

**Figure 8:**
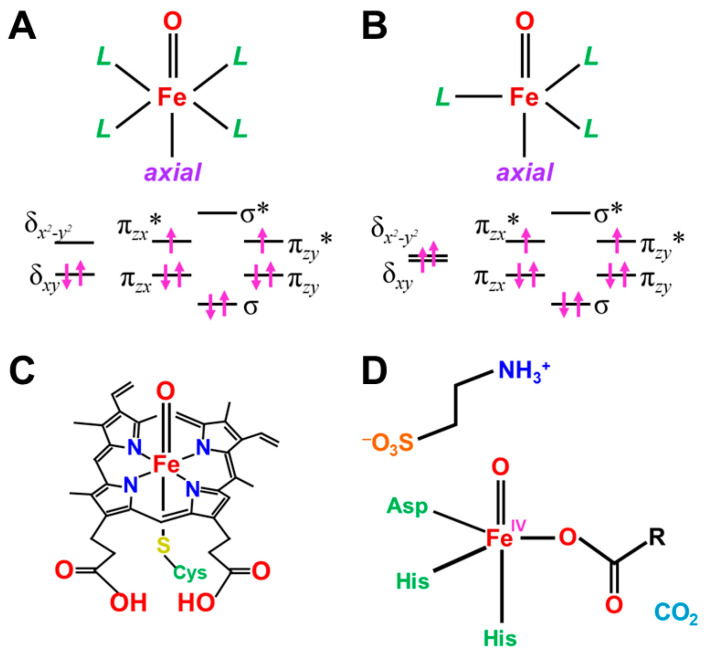
The molecular orbital energy levels for the Fe=O species with (**A**) octahedral (heme-type), (**B**) trigonal bipyramidal (TBP) (non-heme type) ligand fields, (**C**) Porphyrin with the (**A**)-type electronic structure and (**D**) TauD with the (**B**)-type electronic structure [83,84,85,86,87,88,89,90]. L denotes the ligand employed. The vertical arrow notations ↑ and ↓ have been used for schematic illustrations of the electrons with the up- and down-spins, respectively. The energy levels of the d-orbitals are expressed by the horizontal bars.

Here, the effective bond orders of the transition metal-oxo species with the octahedral (O*_h_*) ligand fields are briefly investigated. The eight different orbitals in Equation (12) are obtained for the L(O*_h_*)M=O bonds with the octahedral ligand (O*_h_*) field, as shown in Figure 8A. For example, the orbital energy levels and occupation numbers of the eight orbitals for Fe(IV)=O species are illustrated in Figure 8A [83,84,85,86]. The one *dσ-pσ* and two *dπ-pπ* bonding orbitals are doubly occupied, and the corresponding antibonding (*dσ-pσ*)* orbital is zero for Fe(IV)=O species. On the other hand, the antibonding (*dπ_xz_-pπ_xz_*)* and (*dπ_yz_-pπ_yz_*)* orbitals are singly occupied MOs (SOMO), as illustrated in Figure 8A. The orbital energy gap between *dδ_xy_* and *dδ*_*x*2−*y*2_ is usually large for the pseudo O*_h_* ligand fields, such as heme (Por) ligand plus axial ligand L in Figure 8C, providing the doubly occupied *dδ_xy_* orbitals. Therefore, PorLFe(IV)=O species have the ground triplet state (*S* = 2/2) [27,81] because of the orthogonality between (*dπ_xz_-pπ_xz_*)* and (*dπ_yz_-pπ_yz_*)*. Therefore, the bond order for PorLFe(IV)=O species in the ground triplet state is calculated to be 2.0 (= (3 × 2 − 1 × 2 − 0 × 2)/2). 

On the other hand, the orbital energy gap between *dδ_xy_* and *dδ*_*x*2−*y*2_ is very small for the pseudo trigonal bipyramidal (TBP) geometries. such as TauD systems [87], as illustrated in Figure 8B; therefore, *dδ_xy_* and *dδ*_*x*2−*y*2_ are singly occupied, providing the triplet configuration. Interestingly, TBPLFe(IV)=O bonds in TauD of Figure 8D have the total quintet state (*S* = 4/2) because of ferromagnetic effective exchange interactions between the orthogonal 3d orbitals. However, the effective bond orders for the Fe(IV)=O bonds are not changed with the spin transition from the triplet and quintet states because of no essential contribution of dδ orbitals to the Fe-O bonding.

PorLFe(V)=O species in P450 enzymes [1,2,3,4,5,6,7,8,9,10,11,12,13,14,15,16,17,18,19,20,21] is obtained by one-electron oxidation of the Fe(IV)=O bond, for example, by the removal of one electron from the (*dπ_yz_-pπ_yz_*)* or (*dπ_xz_-pπ_xz_*)* singly occupied MO) in Figure 8A. The π*-LUMO of Fe(V)=O plays an important role for mono-oxygenation reactions. The bond order for the PorLFe(V)=O species in the ground doublet state is 2.5 (= (3 × 2 − 1 × 1 − 0 × 2)/2). Many excited configurations are also conceivable for PorLFe(V)=O and PorLFe(IV)=O species, as summarized in Table 5. The occupation numbers of many excited configurations are shown by the occupation numbers of the eight orbitals in Figure 8A,B. The bond orders for these excited states are obtained by considering the occupation numbers of the molecular orbitals in Table 5. 

Interestingly, the one electron transfer from porphyrin to the Fe(V)=O occurs to provide the well-accepted compound I (Cpd I) structure Por(+•) LFe(IV)=O [83,84,85,86,87,88,89,90], entailing the reduction of the effective bond order of the Fe=O bond. The intermolecular one-electron transfer (OET) from electron donor (D) to PorLFe(V)=O is also feasible, providing [PorLFe(IV)-O^−^ + D(+•)], which plays an important role for the ET-PT process [21]. The electron-delocalization between LUMO (Fe(V)=O) and HOMO (H-CR) occurs in the case of the hydroxylation via the ET-PR process, reducing the activation barrier for hydrogen atom transfer (HAT). The cationic intermediate has been proposed as one of the active species for mono-oxygenations [65,66,67,68,69,70].

### 4.4. Reduction of Effective Bond Order and Radical Reactivity of High-Valent Fe=O 

Here, the concept of the effective bond order is examined in relation to the radical reactivity [25,67]. The Fe(V)=O and Mn(IV)=O bonds are formally isolobal and isospin states with the 3d^4^-2p^6^ electron configurations, providing the molecular orbital descriptions, as shown in Equation (12b). Therefore, the ^2(4)^Σ state of them is expressed by the following occupation numbers: X = 2 (1), Y = 0 (1), Z = 1 (1), and W = 0 (0) for heme (non-heme) ligands in Equation (12b). On the other hand, the ^2(4)^Π state is expressed by the following occupation numbers: X = 2 (1), Y = 0 (1), Z = 0 (0), and W = 1 (1) for heme (non-heme) ligands in Equation (12b). The ^2^Δ state with the triple bond is formally obtained by the following occupation numbers: X=2, Y=1, Z=W=0 for the Fe(V)=O species.

(*dδ_xy_*)^X^ (*dδ_x_*_2−*y*2_)^Y^ (*dσ-pσ*)^2^ (dσ-pσ*)^Z^ (*dπ_xz_-pπ_xz_*)^2^ (dπ_xz_-pπ_xz_*)^0^ (*dπ_yz_-pπ_yz_*)^2^ (dπ_yz_-pπ_yz_*)^W^ (12b)

**Figure 9:**
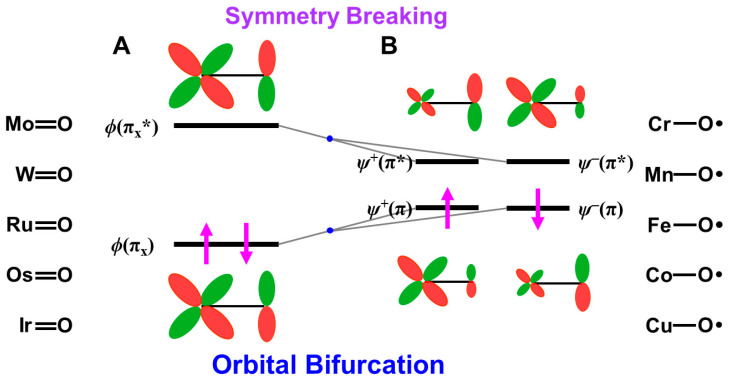
(**A**) The closed-shell (*dπ-pπ*) HOMO and (*dπ-pπ*)* LUMO of M=O compounds, (**B**) BS orbitals obtained by the HOMO-LUMO mixing responding for the oxyl-radical character [25,27,81,82]. The high-valent 3d metal-oxo bonds often exhibit the oxyl-radical character. The up and down arrows are denoted the up and down spins of electron, respectively.

The orbital energy gaps between the doubly occupied bonding (*dπ_qz_-pπ_qz_*) (HOMO) (q = x or y) and vacant antibonding (*dπ_qz_-pπ_qz_*)* (LUMO) (q = x or y) are usually large for LM(X)=O (M(X) = Fe(V), Mn(IV)) species with relatively short M(X)-O distances. The LUMO is responsible for the nucleophilic attack of electron-rich compounds. On the other hand, the energy gap becomes small in the case of the elongated M-O distances, indicating the instability in Equation (14). The mixing between (*dπ_qz_-pπ_qz_*) and (*dπ_qz_-pπ_qz_*)* (q = x or y) in Equation (16) took place, providing BS orbitals that were mainly localized on the M and O-sites, respectively, which is compatible with the M-oxo bond with the strong oxyl-radical character; PorL↑•M(X−1)-O•↓, as shown in Figure 9. The orbital overlap (*T_i_*) between the SP orbitals becomes smaller than 0.6. The occupation numbers of HOMO (*dπ_qz_-pπ_qz_*) and LUMO (*dπ_qz_-pπ_qz_*)* (q = x or y) are given by X = 2, Y = 0, Z = (1 + *T_i_*) and W = (1 *− T_i_*), respectively. Thus, the high-valent LFe(V)=O and Mn(Y)=O (Y = V, IV) exhibit electrophilic and radical reactivity, depending on the oxyl-radical character [62,63,64,65,66,67,68,69,70], which is controlled by types of ligand (L) and environments.

The SP orbitals are also obtained for other HOMO-LUMO pairs with orbital energy gaps. Table 5 summarizes the bond orders (BO) for the high-valent iron-oxo bonds in the octahedral and trigonal bipyramidal ligand fields [25,26,27,28,29,30]. From (No. 1~4) Table 5, the effective bond order (BO) is 2.5 for the ^2(4)^[PorLFe(V)=O] with singly occupied ^1^(*dπ_qz_-pπ_qz_*)* or ^1^(*dσ-pσ*)* since ^2^(*dδ_xy_*) pair in Figure 8A or ^3^[^1^(*dδ_xy_*)^1^(*dδ*_*x*2−*y*2_)] in Figure 8B does not contribute to the effective bond order of the Fe=O bonds. The effective bond orders for Fe(V)=O decrease with the increase in the occupation numbers of the antibonding orbitals, as shown in Table 5. The BO value is 2.0 for ^3(5)^Fe(IV)=O with singly occupied ^1^(*dπ_qz_ − pπ_qz_*)* (*q* = x or y) or ^1^(*dσ-pσ*)*. The BO values for Fe(IV)=O in the excited states also decrease with the increase in occupation numbers of the antibonding orbitals. 

The effective bond order of the ^2(4)^Σ state of Fe(V)=O with the ^1^(*dσ-pσ*)* is given by 0.5 + 2T because of the SP of two *dπ-pπ* orbitals, as shown in No. 7 in Table 5. On the other hand, the effective bond order is given by 1.5 + *T* for ^2(4)^ Π state, as shown in the Oh ligand field (No. 8) and trigonal bipyramidal (TBP) ligand field (No. 12), indicating the SP structure Fe(IV)-O• with a strong dπ oxyl-radical character, which is responding for radical reactivity (see later). Several other cases are summarized in Table 5. 

The effective bond order of the singlet, triplet, and quintet states of Fe(IV)=O is given by 2.0 because two (*dπ-pπ*)* orbitals are singly occupied, as shown in the O*_h_* ligand field (No. 16, 13) and trigonal bipyramidal (TBP) ligand field (No. 22). The SP structures are also conceivable for Fe(IV)=O, providing several Fe(III)-O• structures. Some examples are summarized in Table 5. Many other electronic and spin structures are also conceivable for Fe(X)=O (X = IV, V) after one electron capture from electron donors (D), as shown in Figure 6. They are also constructed by the same procedures discussed above. The singlet state with the X = Y = 2 and Z = W = 0 is conceivable even for the Fe(IV)=O bond. The designs of appropriate ligand fields for 3d M=O species are feasible for the generation of target electronic and spin states under investigation because of quasi-degeneracy among possible intermediates with the same effective bond orders.

### 4.5. Isolobal and Isospin Analogy among Fe(IV)=O, O=O, and O for Chemical Reactions

Here, the isolobal and isospin analogy among metal-oxo bonds and molecular and atomic oxygens is examined to elucidate possible reaction modes in relation to the multiple intermediate models for mono-oxygenation [27,29,81,82]. The energy gap between the HOMO-LUMO in Equation (14) is zero for Fe(IV)=O, O=O, and O because of the complete degeneracy of the HOMO and LUMO. Therefore, the ground state of these species is triplet because of the Hund rule, indicating the isospin analogy among them. These species have two degenerated singly occupied MO (SOMO) configurations. The one-electron reduction of them provides Fe(III)=O, superoxide anion (O_2_^−^•), and oxygen anion (O^−^•), which are isospin doublet states. Their two-electron reduction states are Fe(II)=O, molecular oxygen dianion (O_2_^2−^), and atomic oxygen dianion (O^2−^), which are formally singlet states. On the other hand, their one-electron oxidation states are the doublet Fe(V)=O, O_2_^+^•, and O^+^•. 

The bond orders are 2.5, 2.0, 1.5, and 1.0 for O_2_^+^• (Fe(V)=O), O_2_ (Fe(IV)=O), O_2_^−^• (Fe(III)=O), and O_2_^2−^ (Fe(II)=O), respectively, where the isolobal iron-oxo species are given in parentheses. The O-O distances are 1.123, 1.207, 1.280, and 1.49 Å for O_2_^+^•, O_2_, O_2_^−^•, and O_2_^2–^, respectively [21,37,41], indicating its elongation with the increase in the occupation numbers of the antibonding orbitals. A similar tendency is expected for the isolobal and isospin iron-oxo bonds. Indeed, the Fe-O distances are 1.62~1.68 and 1.81 Å, respectively, for the Fe(IV)=O and Fe(III)=O [78,79].

**Figure 10:**
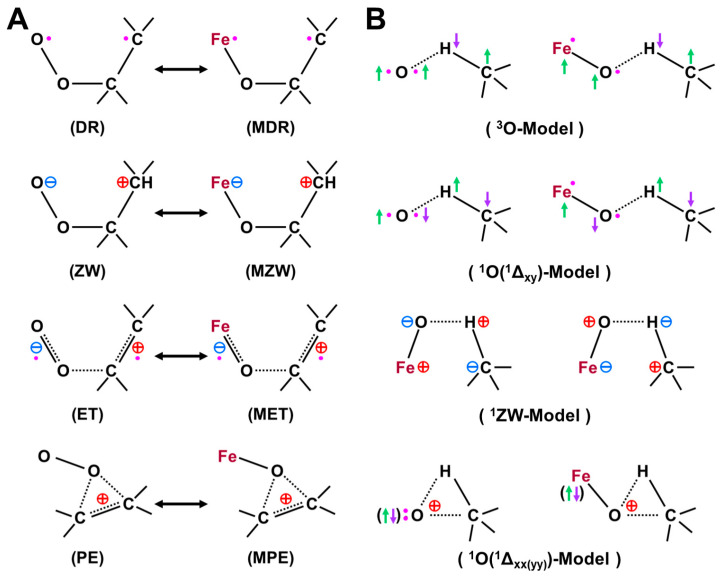
(**A**) Isolobal and isospin analogy between molecular oxygen (O=O) and Fe(X)=O (X = IV, V) for addition reactions of C=C double bonds in accordance with four mechanisms in Figure 7, (**B**) triplet (^3^P) and singlet (Δ_xy_) (with singly occupied p_x_ and p_y_ atomic orbitals) atomic oxygen (O) models for hydrogen radical abstraction reactions, proton transfer model in the zwitterionic (ZW) state and singlet oxygen (Δ_xx_) (with doubly occupied p_x_ atomic orbital and vacant p_y_ atomic orbital) O-model for the non-radical oxygen insertion reactions [27,29,81,82]. The ZW intermediate in (**A**) is responding for the NIH shift in aromatic compounds. These intermediates are responding for the multiple intermediates model for P450 and non-heme iron-oxo compounds (see text).

**Figure 11:**
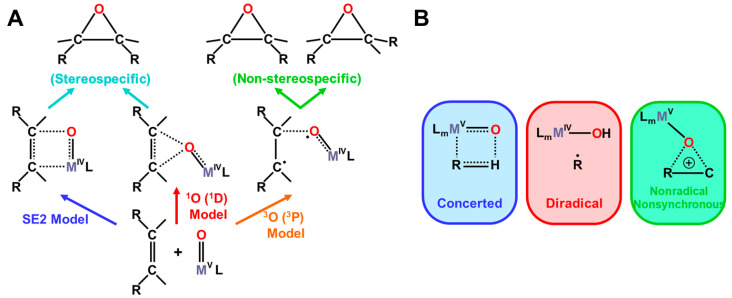
(**A**) Stereochemistry of mono-oxygenation reactions of the C=C double bonds by high-valent transition metal oxo (M(X)=O, X = IV, V) compounds, such as Cpd I, on the basis of the isolobal and isospin analogy between the M(X)=O and atomic oxygen [27,81]. The terminology of SE_2_ was used for NIH shift in the cationic intermediate state of aromatic molecules, such as tryptophan [21] and (**B**) the four-center (essentially concerted), radical and insertion type mechanisms [27,81] of the hydroxylation reactions of alkanes on the basis of the same isolobal and isospin analogy.

The isolobal and isospin analogy among Fe(IV)=O, O=O, and O [27,81,82] provides a guiding principle for an understanding of the mechanisms of oxygenation reactions. For example, the singlet molecular oxygen (^1^Δ_xx(yy)_) has the vacant LUMO, which is related to four different reactions in Figure 10; (a) 1, 4-diradical (DR) reaction (FR), (b) zwitterionic (ZW) reaction (FN), (c) electron transfer (ET) reaction (AR), and (d) perepoxide (PE) reaction (AN) [81]. Similar mechanisms are also expected for the excited singlet ^1^Fe(IV)=O (^1^Δ_πxπx_) and the ground doublet ^2^Fe(V)=O species with the vacant LUMO like the singlet O_2_ (^1^Δ_xx(yy)_) and O(^1^Δ_πxπx_), as illustrated in Figure 10A [25,27,29]. Therefore, these species may undergo the nonradical nonsynchronous reactions [65,66,67,68,69,70], such as stereospecific epoxidation and oxygen insertion, as shown in Figure 11. 

On the other hand, the triplet O (^3^P) model is applicable to elucidate non-stereospecific epoxidations via 1,4-singlet and triplet diradical intermediates and non-stereospecific oxygen insertion via hydrogen abstraction reaction by the ^3^Fe(IV)=O and ^2^Fe(V)=O with strong oxyl-radical character; ↑•Fe(IV)-O•↓, as illustrated in Figure 10B and Figure 11. Dawson and Sono [14] summarized early spectroscopic results for P450. Meunier summarized a number of experimental results for mono-oxygenations by Fe(X)=O before 1994 [16]. Judging from the available experimental results [16,18], the four reaction mechanisms in Figure 10; Figure 11 were found to be useful for understanding and explanation of chameleonic experimental results for mono-oxygenations by several M(X)=O (M = Fe, Mn, etc.; X = IV, V) complexes [42,43,44,45,46,47,48,49,50,51,52,53,54,55,56,57,58,59,60,61,62,63,64,65,66,67,68,69,70]. 

**Figure 12:**
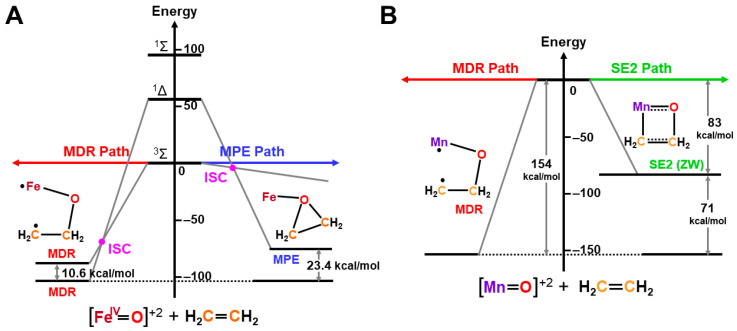
(**A**) Comparison between 1,4-diradical (DR) and perepoxide (PE) reaction pathways for singlet (^1^Δ) and triplet (^3^Σ) Fe(IV)=O in Figure 10. The 1,4-diradical (DR) pathway is more favorable than the perepoxide pathway by both spin states. The spin crossover takes place in the 1,4-diradical process, indicating a two-states model. (**B**) Comparison between 1,4-diradical and four-center (electrophilic) reaction pathways for Mn(IV)=O in Figure 11A. The 1,4 diradical pathway is more favorable than the four-center pathway, confirming the predictions based on the isolobal and isospin analogy [27,81].

The ab initio UHF calculations were performed to examine the scope and applicability of the above isolobal and isospin analogy [27,81]. Figure 12 illustrates the calculated state correlation diagrams for the chemical reaction between Fe(IV)=O and ethylene. The 1,4-DR pathway (FR) was more favorable than the PE pathway (AN) for the mono-oxygenation in this simple model. The curve crossing between the singlet and triplet states took place along the 1,4-DR pathway, as shown in Figure 12A [27], indicating the two-state reactivity. The 1,4-diradical addition mechanism was also more favorable than the four-centered mechanism in the case of Mn (X)-O•, as illustrated in Figure 12B. Thus, high-valent Fe=O and Mn=O exhibited strong oxyl-radical characters, which were responding to non-stereospecific epoxydation reactions by various synthetic model complexes of P450 enzymes [42,43,44,45,46,47,48,49,50,51,52,53,54,55,56,57,58,59,60,61,62,63,64,65,66,67,68,69,70]. 

The analogy between hydrogen radical abstractions (HRA) by triplet atomic oxygen (^3^O) and ^3^Fe=O in Figure 11 has been feasible [83,84,85,86,87,88,89]. On the other hand, a singlet oxygen atom (^1^O in the ^1^D state) undergoes an insertion reaction into the R-C-H bond to afford R-C-OH. Therefore, we proposed selection rules for mono-oxygenation reactions by transition metal oxo (M=O) bonds, as shown in Figure 11A [27,54]; (1) singlet O (^1^D) model for stereospecific mono-oxygenation and (2) triplet O (^3^P) model for non-stereospecific mono-oxygenation reactions. Multiple state mechanisms [27] were also proposed for mono-oxygenations of alkanes [83,84,85,86,87,88,89], as illustrated in Figure 11B. Thus, the high-valent transition metal oxides with strong oxyl-radical character, M(X)-O• (X = III, IV) were expected to undergo radical addition and abstraction reactions in (A) and (B) in Figure 11 [27,30,81]. 

In the 1980s, our computational facility [27] was hardly possible to perform BS computations based on more realistic models of porphyrin metal complexes. In 2004, Koizumi et al. [79] performed the ab initio UDFT computations of PorM(V)=O compounds (M = Mn, Fe), elucidating the instability of the *dπ_yz_-pπ_yz_* bond. The HOMO-LUMO mixing indeed entailed the spin-polarized (SP) bond, Por ↑↑M(IV)-O•↓, where spin densities on the Fe and O-sites are about 2.0 and −1.0 because of the strong SP effect. Therefore, the UDFT computations supported early theoretical models for radical reactions via the oxygen-radical site of our triplet O (^3^P) model for high-valent M(V)=O bonds in Figure 11. The radical coupling (RC) mechanism for the O–O bond formation [79] was also proposed on the theoretical grounds at that time in relation to the O–O bond formation for water oxidation [80].

Por↑↑ M(IV)-O•↓ + ↑•O-M(IV)↓↓Por → Por↑↑M(IV)-O-O-M(IV)↓↓Por(22)

### 4.6. Quantum Resonance between BS Solutions (RBS) and Diradical Character

Here, the concept of quantum resonance is introduced to the recovery of the broken-symmetry to obtain the symmetry-projected states for the EPR spectroscopy and several chemical indices [25]. The orbital symmetry breaking in Equation (14) and in Figure 9 is a fundamental concept for understanding and explanation of the structure and reactivity of high-valent transition metal oxo compounds at the MO theoretical level, as illustrated in Figure 10, Figure 11 and Figure 12 [27]. However, it does not mean the true broken-symmetry (BS) state in the phase transition of the solid-state physics, indicating the necessity of the quantum-mechanically (QM) correct description of diradicals for EPR spectroscopy and other spectroscopies [14,18,37,38,39,40,41]. In fact, the recovery of the broken-symmetry (BS) occurs via the quantum resonance in the case of finite systems without phase transitions [25,27]. 

**Figure 13:**
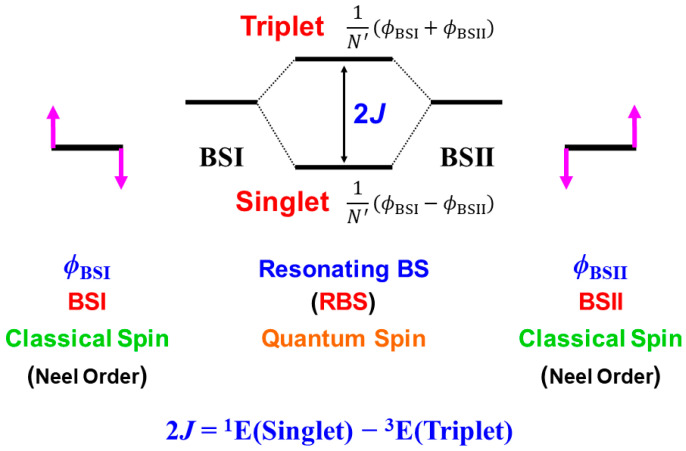
The quantum resonance between the broken-symmetry (BS) configurations (BSI and BSII) degenerated in energy provides the two resonating broken-symmetry (RBS) states, which are equivalent to the pure singlet (S) and triplet (T) states. The energy gap between the S and T states is defined as 2*J* in the chemist’s notation [25,27]. The size-consistent approximate spin projection (AP) for the LS BS solution (AP BS) is performed with the use of the total energy of the HS BS solution [25,27,28]. The configuration interaction (CI) by the use of the natural orbitals (UNO) of the BS solutions [25,32,95,98] is also performed for refinements of the AP (LS) BS energy [25,27,32]. The up and down arrows are denoted the up and down spins of electron, respectively.

The BS configuration with the antiferromagnetic (AF) (↑•…•↓) spin pair ($\Phi \left(BSII\right)=\left|\Psi_{i}^{+}\alpha \Psi_{i}^{-}\beta \right|$) is employed for the qualitative orbital explanation of the singlet-type diradical configuration, as shown in Figure 10. However, such an expression does not mean the true classical anti-parallel spin alignment (Neel order in the molecular magnetism in infinite systems) [25,27]. In fact, the other AF BS configuration with the (↓•…•↑) spin alignment ($\Phi \left(BSII\right)=\left|\Psi_{i}^{-}\alpha \Psi_{i}^{+}\beta \right|$) is also feasible for diradicals. Two BS configurations are completely degenerated in energy, as illustrated in Figure 13. Therefore, the quantum resonance between these configurations occurs under the non-zero overlap (*T_i_*) condition [25] to provide the resonating BS (RBS) states given by the in (+) and out (−) of phase combinations as follows:

(23a) $$\Psi_{RBS\left(+\right)}=\frac{1}{\sqrt{2}}\left[\Phi \left(BSI\right)+\Phi \left(BSII\right)\right]=\left|\frac{\Psi_{i}^{+}\Psi_{i}^{-}\left(\alpha \beta -\beta \alpha \right)}{\sqrt{2}}\right|$$

(23b) $$\Psi_{RBS\left(-\right)}=\frac{1}{\sqrt{2}}\left[\Phi \left(BSI\right)-\Phi \left(BSII\right)\right]=\left|\frac{\Psi_{i}^{+}\Psi_{i}^{-}\left(\alpha \beta +\beta \alpha \right)}{\sqrt{2}}\right|$$

The RBS(+) and RBS(−) states are nothing but pure singlet (S) and triplet (T) states, respectively, which are responsible for the QM requirement for finite systems without magnetic phase transitions [15]. The energy gap between the S and T states is defined as the effective exchange integral (*J*) in the spin Hamiltonian model for EPR (see later) [25,27,39].

2*J* = ^1^E(RBS(+)) − ^3^E(RBS(−)) (24)

where ^X^E(Y) denotes the total energy of the spin state (X) of the RBS state Y. The *J* values have been determined by the magnetic susceptibility experiments and EPR spectroscopy [37,38,39,40,41]. The RBS state is now accepting as an entangled state in the quantum information and computing [33,34,35,36]. 

The PorLFe(III)OO^−^ was often regarded as the exchange coupled system between PorLFe(III) cation radical (*S* = 1/2) and superoxide anion radical (*S* = 1/2) [14,37,38,39,40,41]. Therefore, the exchange coupling between them provided the ground singlet and excited triplet states, as shown in Equation (23) [14,27,39]. The *J* value becomes negative in sign in our chemist’s definition in Equation (24). The spin Hamiltonian model is revisited in relation to the EPR spectroscopy of possible spin states of the reaction intermediates [14,39] later.

The RBS(+) solution in Equation (23a) is re-expressed by symmetry-adapted MOs in Equation (14) in order to obtain the effective bond order for the pure singlet state and diradical character [93]

(25) $$\Psi_{RBS\left(+\right)}=\frac{1}{\sqrt{2\left(1+T_{i}^{2}\right)}}\left\{\left(1+\cos2\theta_{i}\right)\left(\varphi_{HOMO-i}\bar{\varphi_{HOMO-i}}\right)-\left(1+\cos2\theta_{i}\right)\left(\varphi_{LUMO+i}^{*}\bar{\varphi_{LUMO+i}^{*}}\right)\right\}$$

where the first and second terms denote the ground and doubly excited configurations, respectively, in the 2 × 2 configuration interaction (CI) model based on the natural orbitals (UNO) of the BS solutions [25,32,95,98]. The refined effective bond order (*B*) is expressed with the occupation numbers of the bonding and antibonding UNOs by the projected BS ($\Phi_{RBS\left(+\right)}$) and UNO CI [25]

(26a) $$B=\frac{n_{i}\left(RBS\left(+\right)\right)-n_{i}^{*}\left(RBS\left(+\right)\right)}{2}=\frac{{\left(1+T_{i}\right)}^{2}-{\left(1-T_{i}\right)}^{2}}{2\left(1+T_{i}^{2}\right)}=\frac{2T_{i}}{1+T_{i}^{2}}$$

(26b) $$=\frac{2b_{i}}{1+b_{i}^{2}}\geq b_{i}$$

The effective bond order (*B*) after quantum resonance, namely, the elimination of triplet contamination in the AF configuration, is larger than that (*b*) of the BS solution itself. The radical character (*Y*) is defined by twice of the weight of the doubly excited configuration (W_D_) under the delocalized MO (canonical UNO) CI approximation as [26,27,28,29,30,32]

(27a) $$Y=2W_{D}=\frac{{\left(1-T_{i}\right)}^{2}}{1+T_{i}^{2}}=1-\frac{2T_{i}}{1+T_{i}^{2}}$$

(27b) =1−B

The metal-oxyl-radical character (*Y*) can be in turn calculated by the weight of the doubly excited configuration (*W_D_*) obtained by UNO CI, CAS CI [32,98], and CASSCF [31,99]. The radical character *Y* is directly related to the decrease in the effective bond order *B*. Chemical indices *b*, *B*, and *Y*, are mutually related in the present BS MO and beyond BS approach to M=O [80,100]. 

The BS UB3LYP/BS II computations elucidated that the *dπ-pπ* bond of ^4^Σ (^4^Π) state) state of the Mn(IV)=O exhibits 71 (54)% (Y = 0.71 (0.54)) diradical character [78,79]. Therefore, the effective bond order (B) was calculated to be 0.29 (0.46) for these states, respectively. The strong oxy-radical character of Mn-oxo bonds was consistent with the 1,4-diradical addition mechanism in Figure 12B. Thus, chemical indices obtained by the natural orbital (NO) analysis of the BS solutions are useful for understanding and explanation of radical reactivity of high-valent metal-oxo bonds [42,46,47,48,49,50,51,52,53,54,55,56,57,58,59,60,61,62,63,64,65,66,67,68,69,70,83,84,85,86,87,88,89,90]. The successive UNO CI is in turn useful for refinements of these indices at the BS level of theory. 

### 4.7. Spin Density and Pair and Spin Correlation Functions for Singlet Diradicals

In this section, the concepts of correlation and spin correlation functions [25,130] have been revisited in relation to the possible roles of spin density by the BS methods. Spin density is a useful chemical index for radical reactions, as shown in Figure 6. However, the spin density disappears after the spin projection of singlet BS solutions for singlet diradicals via the RBS procedure in Equation (23) [25,27,131]. In the 1970s, the BS methods with significantly larger <***S***^2^> value than the exact *S*(*S*+1) value were negatively discussed based on wrong spin properties such as spin contamination and symmetry dilemma [132]. Therefore, against the symmetry dilemma [132], we tried to elucidate possible positive roles of the spin density of singlet diradical and open-shell species from the basic principle of “*strong electron and spin correlation effects”* [25,130]. Our fundamental idea was to examine the pair and spin correlation functions related to strong correlation and spin correlation effects in these radical species [25,130]. The second-order pair and spin correlation functions of the BS solutions were derived to elucidate the important roles of spin densities for chemical indices for strongly correlated systems. Indeed, the on-site pair function (*P*_2_) for electrons with different spins is indeed given by the following [25,130]:

(28) $$P_{2}\left(r_{1},r_{1};r_{1},r_{1}\right)=\frac{P_{1}{\left(r_{1},r_{1}\right)}^{2}-Q_{1}{\left(r_{1},r_{1}\right)}^{2}}{2}$$

where *P*_1_(***r***_1_, ***r***_1_)^2^ and *Q*_1_(***r***_1_, ***r***_1_)^2^ denote, respectively, the square of density and spin density. This means that the magnitude of the spin density is directly related to the size of the Coulomb hole (mutual repulsion) for electrons with different spins, providing an important theoretical picture that the size of the dot • in the preceding chemical expressions means the magnitude of the Coulomb hole on the theoretical ground. The same physical picture was later derived in the field of the DFT theory [133,134]. 

The Kohn–Sham (KS) DFT model provided spin densities for singlet diradical species, although the exact DFT was believed to provide no spin density like the exact quantum mechanical (QM) models, such as RBS [25]. Therefore, the spin densities of KS SDT were also rationalized on the same idea [133,134], namely, the pair function in Equation (28) [25,130]. The Equation (28) was also applicable to the hybrid DFT model, providing the DFT correlation function for the multi-reference (MR) DFT approach [135]. The spin orbit interactions for spin inversion were not discussed at that time [25,130].

Moreover, the square of the spin density (Coulomb hole) in Equation (28) is related to the unpaired electron density *U*(***r***_1_) responding to the deviation from the single determinant under the BS approximation as [25,130,136,137]

(29) $$U\left(r_{1}\right)=Q^{2}\left(r_{1},r_{1}\right)=Q^{2}\left(r_{1}\right)=\sum n_{i}\left(2-n_{i}\right)$$

The magnitude of the spin densities reported in various recent BS calculations can be understood from the viewpoint of non-dynamical correlations between electrons with different spins, namely, strong electron repulsion effects. From Equation (29), the unpaired spin density *U(r)* is obtained via the occupation numbers of the beyond BS methods, such as CASSSCF [31,99] and UNO CI (CC) [32,100]. Therefore, the spin density index [93] is also introduced to express the characteristic behavior of the spin density as follows:

(30) $$Q(r_{1},r_{1})=\sqrt{U_{i}(r)}=\sqrt{1-T_{i}^{2}},\;Q\left(r_{1},r_{1}\right)=\sum_{i}Q_{i}\left(r_{1},r_{1}\right)$$

The next problem with the symmetry dilemma was to provide an answer to the basic question [132], namely, the possible roles of the sign of spin densities under the BS approximation. In order to elucidate the answer to this question, the spin correlation function [25,130,138] was introduced since it was observed in the case of infinite systems with the neutron diffraction technique [139]. In fact, the spin correlation function *K*_2_(***r***_1_, ***r***_2_) for the BS solution was approximately given by [25,130]

(31) $$K_{2}\left(r_{1},\;r_{2}\right)=\int s\left(1\right)s\left(2\right)P_{2}\left(r_{1},r_{2};r_{1},r_{2}\right)ds\approx Q\left(r_{1}\right)Q\left(r_{2}\right)$$

where *P*_2_ denotes the second-order density matrix. This means that the spin correlation is singlet-type if the sign of spin density product is negative in sign (↑↓) or (↓↑), although such short-time spin order cannot be detected by the neutron diffraction because of the quantum resonance in polyradical species [25]. Therefore, the arrow notations ↑ (or ↓) are used to describe spin correlations between the up and down spins in this review. 

The spin correlation functions in Equation (31) were extended for general Hartree–Fock (GHF) solutions described with general spin orbitals (GSO), two-component spinor, which provide non-collinear spin structures with three-dimensional spin densities [25,130]. Although the spin densities arising from the first-order density *P*_1_(***r***_1_, ***r***_2_) disappear at the pure singlet state, RBS(+) in Equation (25), the unpaired electron density (*U*(***r***_1_)) and spin correlation function (*K*_2_) still exist as important spin and electron correlation indices even in the resonating BS (RBS) and symmetry-adapted multi-reference (MR) beyond BS wave functions such as MR CC [32] and MR DFT [136], complete active space (CAS) CI [32], and CASSCF [31]. Therefore, the sign and magnitude of spin densities in the BS approach should be understood from the above theoretical viewpoints in Equations (23)–(31). The pair and spin correlation functions can be used to elucidate the nature of chemical bonds in the case of RBS and MR approaches [25,32] as alternative indices for spin density at the BS UB3LYP level of theory. 

**Figure 14:**
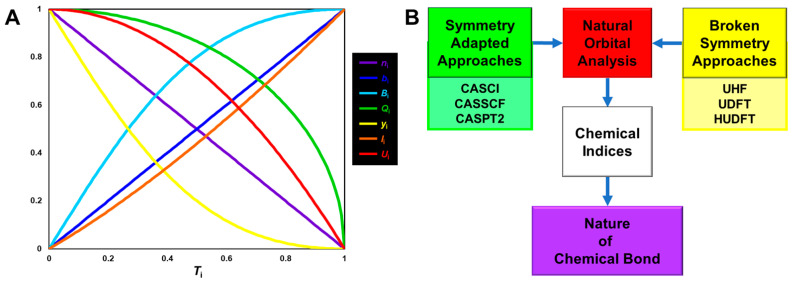
(**A**) Schematic illustration of variations of the chemical indices with the orbital overlap (*T*_i_) between broken-symmetry (BS) orbitals in the BS methods coupled with spin projections [25,32,93]. Definition and derivation of the chemical indices are given in Equations (24)–(32). (**B**) Natural orbitals (UNO) are obtained by the BS computations in the single Slater determinant approximation. The UNO and their occupation numbers [32,100] are used for construction of multi-configurations for CASSCF and CASPT2 [31,99,101]. Some of chemical indices are calculated with the occupation numbers of the natural orbitals of BS and beyond BS methods, indicating the theoretical bridges between them.

Recently the information entropy [140,141] is a useful measure of quantum effects in SCES. The information entropy (*I_n_*) for chemical bonds is defined by the occupation number as

(32) $$I_{i}=-n_{i}\ln n_{i},\;I_{c}=-2\ln2,\;I_{n}=I_{i}/I_{c}$$

where *I_i_* and *I_c_* denote the information entropies defined by the occupation numbers of the partially and fully occupied orbitals. The effective bond order *I*_n_ is defined by the ratio of them. Its behavior is similar to that of the effective bond order (*b*), as shown in Figure 14.

Figure 14A illustrates functional behaviors of the chemical indices against the orbital overlap *T_i_* (i = HOMO, HOMO–1, …). The effective bond order (*b*) is decreased linearly with the decrease in the orbital overlap (*T_i_*). The effective information entropy (*I_n_*) also indicates a similar behavior. The spin density *Q_i_* (green line) increases sharply, even in the large orbital overlap region *T_i_* > 0.6, indicating a strong radical character. This characteristic behavior is partly responsible for the spin contamination effect in the BS solution [93]. On the other hand, the diradical character (*Y_i_*) (blue line) obtained after the elimination of the spin contamination remains to be lower than 15% in this region, but it increases sharply in the strong diradical region (*T_i_* < 0.4) [26]. Thus, Figure 14A is useful for the understanding of the functional behaviors of the chemical indices. 

**Figure 15:**
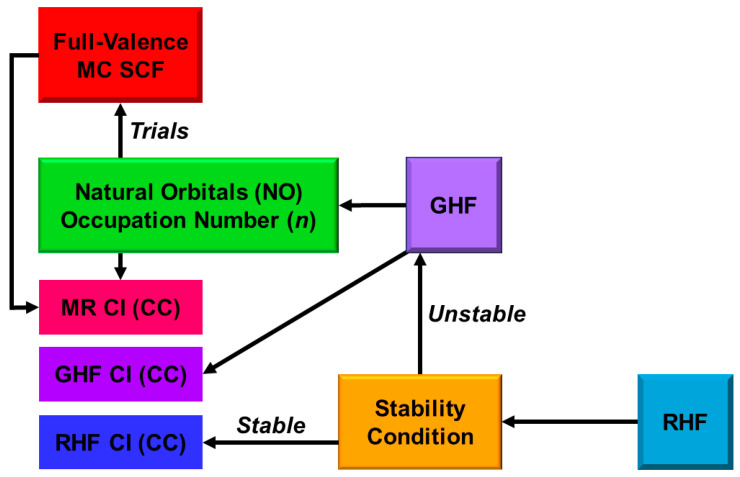
Early proposal of theoretical methods for investigation of electronic and spin states of quasi-degenerated systems [32]. The general BS methods including UHF, generalized HF (GHF) using general spin orbitals (GSO), Hartree–Fock–Bogoliubov (HFB) [25], etc. under the single determinant approximation (meal-field theory) are constructed for the first step for the systems. The natural orbital analysis of these solutions provides the natural orbitals (UNO) and their occupation numbers that are used for multi-determinant configurations (complete active space (CAS) for multi-reference (MR) configuration interaction (CI) and coupled cluster (CC) calculations. UNO CASCCS (S = single excitation) is equivalent to the CASSCF [31].

Several chemical indices defined by the occupation numbers of the natural orbitals are equally applicable beyond BS methods, such as CASSCF, CASPT2, and MR CI(CC) [25,32]. Therefore, they play important roles in conceptual bridges between BS and beyond BS methods, as illustrated in Figure 14B [140]. The natural orbitals (UNO) obtained by BS computations are useful for the construction of reference configurations for these beyond BS computations [32]. In 1980, we proposed fundamental theoretical methods (UNO UCCS (= MCSCF), UNO CI (CC), MR CI) for elucidation of quasi-degenerated electron systems such as those of M=O in Table 3 and Table 4. Figure 15 summarizes our early theoretical approaches to quasi-degenerated electron systems [32]. Thus, the BS-independent particle models [25] are useful as the first step for investigations of the nature of chemical bonds of strongly correlated electron systems (SCES) such as 3d transition metal oxides [25,26,27,28,29,30]. The beyond BS methods [31,32] are necessary as the second step for elucidation of the scope and reliability of the BS computational results. Finally, the quantum computations [33,34,35,36] are expected to be the final step for the elucidation of chemical bonds under investigation.

### 4.8. Quantum Spin Hamiltonian Models for Compound I (Cpd I)

Here, the exchange couplings between local spins are examined in relation to the spin states of the peroxidase, P450, etc. In the 1980s, the spin Hamiltonian (Heisenberg (HB)) model was used for the analysis of experimental magnetic results for transition metal oxides obtained by the magnetic susceptibility methods and EPR spectroscopy [14,37,38,39,40,41]

*H* (HB) = − 2*J*_ab_ ***S***_a_ • ***S***_b_ (33)

where *J*_ab_ and ***S***_q_ (q = a, b) denote, respectively, the effective exchange interaction between local spins and local spin on the site q (q = a, b). We derived a computational scheme of the *J*_ab_ values by using energy gap between the low-spin (LS) and high-spin (HS) BS solutions as [25,27,28]

*J*_ab_ = [^LS^*E*(Y) − ^HS^*E*(Y)]/[^HS^<***S***^2^>(Y) − ^LS^<***S***^2^>(Y)] (34)

where *^X^*E(Y) and *^X^*<***S***^2^>(Y) were, respectively, the total energy of the spin state *X* by the computational method Y and the total spin angular momentum of the LS BS solution. Equation (34) was derived for the BS method after approximate spin projection (AP) [25,27,28]. Therefore, it was also useful for symmetry-adapted multi-configuration (MC) methods [31,32], such as CASCI and CASSCF [25,99]. The size-consistent total energy of the AP LS BS solution without spin contamination [140] has been given by using the *J* value as

^LS^*E*(AP BS) = ^LS^E(BS) + *J*_ab_ [^LS^<***S***^2^>(BS) − *S*(*S* + 1)] (35)

where *S*(*S* + 1) is the total spin angular momentum for the LS (2*S* + 1) state. Therefore, the second term in Equation (35) denotes the energy correction for the LS BS solution with the total spin quantum number ^LS^<***S***^2^>(BS), which often involves the HS spin contamination contribution for the exact LS state with the spin quantum number *S*(*S* + 1).

**Figure 16:**
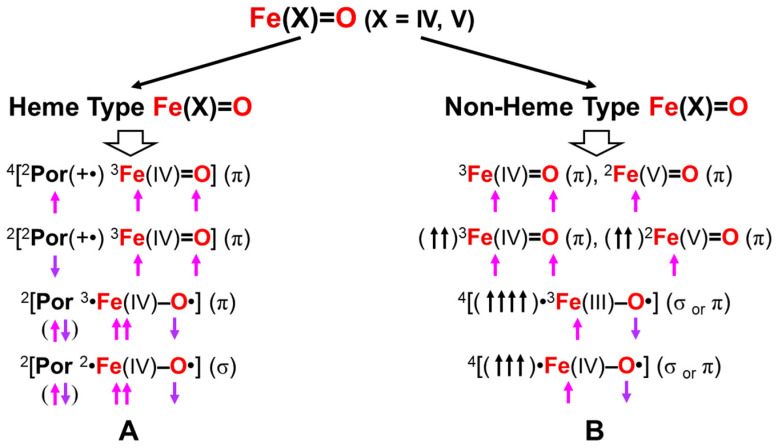
Classifications of the high-valent iron-oxo compounds into (**A**) heme-type Fe(X)=O (X = IV, V) with the (δ_xy_)^2^ configuration and (**B**) non-heme-type Fe(X)=O (X = IV, V) with the (δ_xy_)^1^(δ_x2−y2_)^1^ configuration. In the case (A), the (π) and (σ) denote π- and σ-type oxyl orbitals, respectively. The ↑ and ↓ denote the up- and down-spins, respectively. The high-valent Por↑•Fe(V)=O (see Figure 3) often exhibits the spin polarization (SP) of the *dπ-pπ* bond, providing the Por↑↑••Fe(IV)-O•↓ (π) under the (δ_xy_)^2^ configuration. On the other hand, the SP of the dσ-pσ bond provides the Por↑↑••Fe(IV)-O•↓ (σ) [147,148,149,150,151,152,153,154,155,156,157,158,159,160,161,162,163,164,165,166,167,168,169,170,171,172,173,174,175,176]. In the case (**B**), the total high-spin ^4^[L↑↑↑•••Fe(IV)-O•↑] and ^3^[L↑↑↑•••Fe(V)-O] are feasible. The SP configurations are also available for the non-heme compounds as discussed in the text [177,178,179,180,181,182,183,184,185,186,187,188,189,190,191,192,193,194,195,196,197,198,199,200,201,202,203,204,205,206,207]. The up and down arrows are denoted the up and down spins of electron, respectively.

Spin Hamiltonian models using the arrow notations based on the sign of *J*_ab_ in Equation (34) have been applied to elucidate spin states of transition metal oxides [25,26,27,28,29]. For example, the compound I (**F**) in Figure 1 was often regarded as the exchange coupled system between porphyrin π cation radical (Por (+↓•) with down spin (*S* = −1/2) and triplet (*S* = 2/2) ground state of L↑•Fe(IV)=O•↑ [37,38,39,40,41]; X = 2 and Y = 0 in Equation (12), providing total doublet (*S*_total_ = (2/2 − 1/2) = 1/2) and quartet (*S*_total_ = (2/2 + 1/2) =3/2) states, ^2^[Por(+•↓) ↑•Fe(IV)=O•↑], and ^4^[Por(+•↑) ↑•Fe(IV)=O•↑], as shown in Figure 16. These exchange-coupled states are used for the two-state model of the Cpd I structure [86]. The back charge transfer from ↑•Fe(IV)=O•↑ core to the (Por (+•↓) part provides a formal ^2^[PorL↑•Fe(V)=O] with a doublet spin state, as illustrated in Figure 3 [15,16,17,18,19,20,21,142,143,144,145,146]. The three-state model of the Cpd I is obtained by including the last structure [146].

The doublet ^2^[Fe(V)=O] core (^2^E state) is often the parent iron-oxo complex of P450 [21] instead of the ^3^Fe(IV)=O complex of non-heme iron-oxo enzymes. As mentioned above, the ^2^[Fe(V)=O] core has the lower-lying π LUMO responding for the nonradical mono-oxygenations; addition reaction in Figure 10A and insertion reaction in Figure 11B [25,27,81]. However, ^2^[PorL↑•Fe(V)=O] often exhibits spin polarization (SP), as illustrated in Figure 9, providing the ^2^[PorL↑↑••Fe(IV)-O•↓] with the oxyl-radical character for radical reactions, as illustrated in Figure 16 [27]. The orbital overlap (*T*_i_) between SP BS orbitals and the effective bond order (*b*) were calculated to be 0.35 [80], indicating the intermediate radical character (*Y* = 38%) and large unpaired electron density (*U*(*r*) = 0.87). 

The ET-PT process is feasible for ^2^[Fe(V)=O] if the substrates are strong donors, such as amines (H-NR). The activation barrier for the ET-PT, namely, the allowed radical (AR) process in Figure 7, is significantly reduced when compared with the homolytic hydrogen radical abstractions (HRA). The kinetic isotope effect for hydroxylation may be reduced for the ET-PT process if the quantum effect is not operative for the PT process. Therefore, the relative stability between the ^2^[Por(+•↓) ↑•Fe(IV)=O•↑] (^2^Cpd I) and ^2^[Por↑•Fe(V)=O] (^2^E) is very important for the understanding of the stereospecific and non-stereospecific reaction modes by P450, as illustrated in Figure 11. 

The LFe(IV)=O core in non-heme enzymes often exhibits the high-spin quintet configuration (high spin (HS), namely, X = Y = 1 in Equation (12)) in non-heme iron-oxo compounds, R_1_R_2_↑↑↑•••Fe(IV)=O•↑ (*S* = 4/2) in Figure 8B. The reduction of the energy gap between the d_xy_ and d_x2-y2_ by the C_3_ ligand is essential for the generation of the *S* = 2 intermediate. The triplet atomic oxygen O (^3^P) model is equally applicable to this high-spin species. The non-heme Fe(IV)=O core may exhibit the SP effect of the dσ-pσ bond, providing the R_1_R_2_↑↑↑↑••••Fe(III)-O•↓ (*S* = 3/2) (No. 26 in Table 5). Thus, the bifurcation of the dσ-pσ bond providing the pσ oxyl-radical character is also one of the important factors for understanding of chemical behaviors of non-heme Fe(IV)=O bonds.

**Figure 17:**
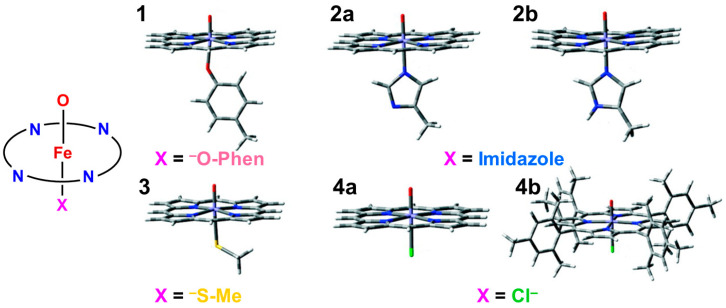
The axial (X) ligands for heme-type compounds, (**1**) X = phenoxy anion for catalase (**2a**) X = imidazole anion for oxidase, (**2b**) X = imidazole for myoglobin (Mb) and hemoglobin (Hb), (**3**) X = thiolate anion, (**4a**) X = chloride anion, and (**4b**) X = chloride anion of the synthetic model complex.

The Equation (34) was applied to elucidate the relative stability between the doublet and quartet spin states of Por(+•)lFe(IV)=O compounds, which were highly sensitive to axial ligands (R), as shown in Figure 17 [20,142,143]. The effective exchange interaction between Por(+•)L doublet ligand part and triplet Fe(IV)=O core was easily calculated by using Equation (34). Figure 17 illustrates the computational models employed. The calculated *J* values were negative in sign for **1** with phenolate anion (L) of catalase and **3** with thiolate anion (L) of P450, indicating the LS doublet ground state. On the other hand, the calculated *J* values were positive in sign for **2** with imidazole anion (**2a**) (L) and neutral imidazole (**2b**) of peroxidase and **4a** and **4b** model complexes with chloride anion (L) in accordance with the high-spin quartet state [51]. The magnitude of the *J* values was small for the model complexes examined in accordance with the experimental results, as shown in Table 6.

### 4.9. Charge and Spin Correlation Diagrams for Mono-Oxygenations by Cpd I

Here, the possible reaction mechanisms for mono-oxygenations by heme iron oxo compounds are derived on the basis of the charge and spin correlation diagrams [147,148,149,150,151,152,153,154,155,156,157,158,159,160,161,162,163,164,165,166,167,168,169,170,171,172,173,174,175,176,208,209,210,211,212,213,214]. To this end, the UMP and approximately spin projected (AP) UMP methods were applied to depict the potential curves of the M=O^+^ (M = Cr, Mn, etc.) species, indicating that only 50% of the binding energy was reproduced by APUMP4 in a sharp contrast to the UCCSD(T) method [78,79]. On the other hand, the HDFT augmented with CASPT2 provided reasonable potential curves for the FeO^+^ + H_2_ reaction [209,211,213], elucidating the two state reactivity patterns of the reactions. Thus, in contrast to organic reactions, two states of different spin multiplicities (TSR) are feasible in the case of oxygenation reactions by 3d transition metal oxides [27,211,212,213]

The spin correlation functions in Equation (34) have indeed indicated that the spin Hamiltonian (Heisenberg (HB)) model is applicable to derivations of spin correlation diagrams for mono-oxygenation reactions [20,21,83,84,85,86,87,88,89,90]. Shaik et al. [147,148,149] first proposed the two states (doublet and quartet) reactivity paradigm of the hydroxylations by P450. The doublet ^2^Cpd I_a_ with the ↓↑↑ spin alignment undergoes hydrogen abstraction reaction, providing the transition structure (TS) that is characterized by the populations of down spin (↓) at porphyrin and up spin (↑) at Fe and up spin (↑) at alkyl radical, as shown in Equation (36a). The down spin (↓) at porphyrin is replaced by the up spin (↑) in the course of hydrogen abstraction reaction by ^4^Cpd I_a_. The conversion of the valence state from Fe(IV) into Fe(III) occurs in the TS region for both ^2^Cpd I_a_ and ^4^Cpd I_a_. 

On the other hand, doublet ^2^Cpd I_b_ with the ↑↑↓ spin alignment also undergoes hydrogen abstraction, providing the Fe(IV) site with two up spins and one down spin at the oxygen site. Therefore, the spin densities on the Fe site are about 1 for ^2^Cpd I_a_ and ^4^Cpd I_a_ and 2 for ^2^Cpd I_b_ [142,143,144,145,146]. Thus, spin structures of three different compound I are directly related to those of transition structures for homolytic hydrogen radical abstractions (HRA) in Equation (19), and Figure 10 and Figure 11B. HDFT computations [146,147,148,149,150,151,152,153,154,155,156,157,158,159,160,161,162,163,164,165,166,167,168,169,170,171,172,173,174,175,176,208,209,210,211,212,213,214] have already been performed to locate the transition structures of hydrogen abstraction reactions by Cpd I. The activation barriers for hydrogen radical abstraction are parallel to the binding energies of the H-C bonds [174]. The detailed computational results are not touched in this review since several excellent review articles have been published, as shown in refs. [83,84,85,86,87,88,89,90,175,176].

^2^[Por(+•↓) ↑•Fe(IV)=O•↑](^2^Cpd I_a_) + H-R → ^2^[Por(+•↓) ↑•Fe(III)-OH…↑•R] (36a)

^4^[Por(+•↑) ↑•Fe(IV)=O•↑](^4^Cpd I_a_) + H-R → ^4^[Por(+•↑) ↑•Fe(III)-OH…↑•R] (36b)

^2^[Por ↑↑••Fe(IV)-O•↓](^2^Cpd I_b_) + H-R → ^4^[Por ↑↑••Fe(IV)-OH…↓•R] (36c)

The isolobal–isospin analogy among Fe=O, O=O, and O [81,82] (see Figure 10) indicates that the electron-transfer [94] coupled proton transfer (ET-PT) mechanism is an alternative possibility for electron-rich substrates [81,82]. The transfer of beta spin of H-R bond to SOMO of ^3^Fe(IV)=O followed by proton transfer (ET-PT) in Equation (18) and Figure 10 is also conceivable for ^2^Cpd I_a_ and ^4^Cpd I_a_, as shown in Equation (37a) if the substrate (H-R) is strongly electron-donating such as amine. On the other hand, the transfer of alpha spin of H-R paired bond to the O• site of ^2^Fe(IV)-O• providing the O^-^ site followed by proton transfer (ET-PT) in Equation (18) and Figure 10 is also feasible for ^2^Cpd I_b_, as shown in Equation (37b). The spin configuration of Fe(III) is *S* = 1/2 for ^2(4)^Cpd I_a_, and the spin configuration of Fe(IV) is *S* = 2/2 for ^2^Cpd I_b_, providing the up and down spins on the alkyl radical site, as shown in Equation (37a,b). Therefore, the sign of spin density on the alkyl radical is an important index for discrimination between ET-PT reactions by ^2(4)^Cpd I_a_ and ^2^Cpd I_b_. Thus, the spin correlation diagrams are also useful for understanding the ET-PT reaction mechanisms. Indeed, we can understand the reaction mechanisms based on the reported signs and populations of spin densities by hybrid DFT computations [83,84,85,86,87,88,89,90,118,119,120,121,122,123,124,125,126,127,128,129,130,131,132,133,134,135,136,137,138,139,140,141,142,143,144,145,146,147,148,149,150,151,152,153,154,155,156,157,158,159,160,161,162,163,164,165,166,167,168,169,170,171,172,173,174,175,176,208,209,210,211,212,213,214,215].

^2(4)^[Por(+•↓(↑)) ↑•Fe(IV)=O•↑)(^2(4)^Cpd I_a_) + H-R → ^2(4)^[Por(+•↓(↑)) ↑•Fe(III)-O^−^…(+↑•H-R) → ^2(4)^[Por(+•↓(↑)) ↑•Fe(III)-OH…↑•R] (37a)

^2^[Por ↑↑••Fe(IV)-O•↓](^2^Cpd I_b_) + H-R → ^2^[Por ↑↑↑•••Fe(III)-O^−^…(+↓•H-R)] → ^2^[Por ↑↑↑•••Fe(III)-OH…↓•R] (37b)

### 4.10. Mono-Oxygenations by Nonheme Iron-Oxo Compounds

Here, early our theoretical models for Fe=O [26,27,28,29,30] have been revisited in relation to non-heme iron-oxo species [177,178,179,180,181,182,183,184,185,186,187,188,189,190,191,192,193,194,195,196,197,198,199,200,201,202,203,204,205,206,207,214,216,217,218,219]. The mono-oxygenations by the non-heme Fe(IV)=O bonds were considered on the basis of the isolobal and isospin analogy between Fe(IV)=O and atomic oxygen (O), as illustrated in Figure 11 [27,81]. Therefore, the electronic and spin configurations in Table 5 are applicable to mono-oxygenations by the Fe(IV)=O with the closed-shell singlet and open-shell triplet δ orbitals in Figure 8A,B. The ^1^[Fe(IV)=O](^1^Δ_πxπy_)(δ_xy_)^2^ with the up spin (π_x_) and down spin (π_y_) (No. 16 in Table 5) is responding for radical abstraction as in the case of ^1^O (D_xy_) [81], providing alkyl radical with the down spin, as shown in Equation (38a). Similar hydrogen radical abstraction (HRA) is also feasible for ^3^{[Fe(IV)=O(^1^Δ_πxπy_)]^3^[^1^(δ_xy_)^1^(δ_x2−y2_)]}, as shown in Equation (38b). The ^3^[Fe(IV)=O](^3^Σ) with (δ_xy_)^2^ and ^5^[Fe(IV)=O](^3^Σ) with (δ_xy_)^1^(δ_x2−y2_)^1^ undergo the HRA reactions like ^3^O (^3^P) [81], providing alkyl radical with the up spin, as shown in Equation (38c,d). The down and up spins are induced on the alkyl radical site, depending on the ^1^Δ_πxπy_ and ^3^Σ states of Fe(IV)=O. Thus, the isolobal and isospin analogy [26,27,81] provided guiding principles for radical reactions of alkanes with non-heme iron-oxo bonds.

^1^[Fe(IV)=O](^1^Δ) + H-R → ^1^[↑•Fe(III)-O•↓…H-R] → ^1^[↑•Fe(III)-OH…↓•R] (38a)

^3^[↑↑••Fe(IV)=O] + H-R → ^3^[↑↑↑•••Fe(III)-O•↓…H-R] → ^3^[↑↑↑•••Fe(III)-OH…↓•R] (38b)

^3^[↑•Fe(IV)=O•↑](^3^Σ) + H-R → ^3^[↑•Fe(III)-OH…↑•R] (38c)

^5^[↑↑↑•••Fe(IV)=O•↑] + H-R → ^5^[↑↑↑•••Fe(III)-OH…↑•R] (38d)

The ET mechanism [94], followed by proton transfer (ET-PT) in Equation (18) and Figure 10, is feasible for ^5^[Fe(IV)=O](^3^Σ) with (δ_xy_)^1^(δ_x2−y2_)^1^ if the substrate (H-R) is strongly electron-donating. The down-spin transfer of the H-R bond to the Fe(IV)=O core with π_2_ SOMO occurs in the first place, providing the high-spin Fe(III) with *S* = 3/2 and the cation radical (up spin) state of H-R (*S* = 1/2) that undergoes the proton shift to afford alkyl radical with the up spin, as shown in Equation (37c). On the other hand, the up spin transfer of the H-R bond to the Fe(IV)=O core with σ* LUMO occurs in the high-spin state, providing the high-spin Fe(III)-O^–^• with the σ-type lone pair (O^−^) and the π-type delocalized spin (↑•) and the cation (down spin) radical of H-R (*S* = −1/2), followed by proton transfer (PT) for the O-H bond formation and radical coupling for the O-R bond formation, as shown in Equation (37d). The linear-like F-O---H-C conformation is favorable for the ET-PT process. The ferromagnetic effective exchange interactions among local spins on the Fe(III) ion plays an important role for stabilization of the high-spin state [25]. The kinetic isotope effect is supposed to be small for the ET-PT processes under the assumption of no quantum effect.

^5^[↑↑↑•••Fe(IV)=O•↑] + H-R → ^5^[↑↑↑•••Fe(III)-O^−^…(+↑•H-R)] → ^5^[↑↑↑•••Fe(III)-O-H…↑•R] (37c)

^5^[↑↑↑•••Fe(IV)=O•↑] + H-R → ^5^[↑↑↑↑••••Fe(III)-O^−^(σ)↑•(π)…(+↓•H-R)] → ^5^[↑↑↑↑••••Fe(III)-HO↑•(π)…↓•R] → ^5^[↑↑↑↑••••Fe(III)-HO-R] (37d)

**Figure 18:**
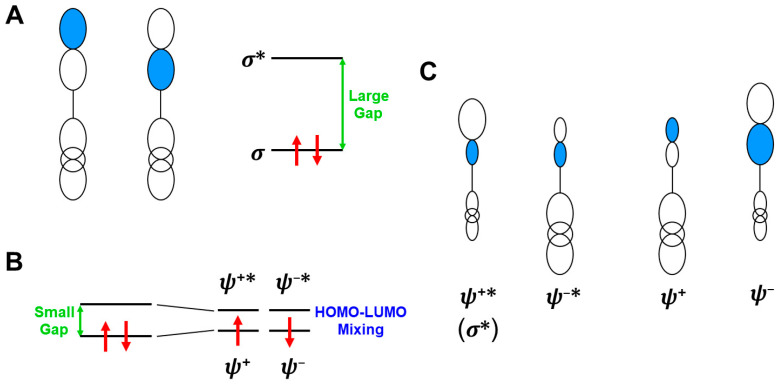
(**A**) The large energy gap between the bonding HOMO (*dσ-pσ*) and antibonding LUMO (*dσ-pσ*)* orbitals responsible for no HOMO-LUMO mixing. (**B**) the small energy gap between the (*dσ-pσ*) and (*dσ-pσ*)* orbitals, indicating the HOMO-LUMO mixing, and (**C**) the broken-symmetry (BS) orbitals localized mainly on the Fe- and O-site, respectively, responsible for •Fe–O• (σ) in Table 1. The up and down arrows are denoted the up and down spins of electron, respectively.

The spin polarization (SP) of the *dσ-pσ* bond may be possible for specific cases, as illustrated in Figure 18. The SP effect of ^5^[Fe(IV)=O](^3^Σ) with ^3^[(δ_xy_)^1^(δ_x2-y2_)^1^] provides the highest spin *S* = 5/2 configuration of Fe(III) and oxygen radical site with the down spin (*S* = −1/2), as shown in Equation (39a). The effective exchange interactions of the Fe (*S* = 5/2) are more favorable than those of the Fe (*S* = 3/2) [25,82]. The homolytic radical abstraction (RA) reaction by pσ radical orbital of the site O is feasible, as shown in Equation (39a), in sharp contrast to those of pπ radical orbital of the site O in Equation (36). Therefore, linear-like transition structures Fe-O-H…R are favorable for hydrogen radical abstractions (HRA) in Equation (39a). The discrimination between the ET-PT in Equation (37d) and RA in Equation (39a) may be feasible from the activation barriers for hydroxylations. The spin-flip excitation from the (*dσ-pσ*) bond to the (*dσ-pσ*)* LUMO is conceivable to afford the highest spin state, as shown in Equation (39b).

^5^[↑↑↑•••Fe(IV)=O•↑] + H-R → ^5^[↑↑↑↑↑•••••Fe(III)-O•↓ + H-R)] → ^5^[↑↑↑↑↑•••••Fe(III)-O-H…↓•R] (39a)

^7^[↑↑↑↑↑•••••Fe(III)=O•↑] + H-R → ^7^[↑↑↑↑↑•••••Fe(III)-OH…↑•R] (39b)

The excited radical states of iron-oxo species stabilized by the ferromagnetic exchange interactions (the Hund rule and/or spin catalysis effect)) of the HS (*S* = 5/2) Fe(III) ion may be generated by photoexcitation, as summarized in Table 5. Thus, the BS level of theory [142,143,144,145,146] is found to be useful for elucidation of spin correlation diagrams and selection rules for oxygenation reactions by iron-oxo compounds.

Over the past two decades, both experimental and theoretical investigations have been extensively performed for the non-heme iron-oxo compounds [177,178,179,180,181,182,183,184,185,186,187,188,189,190,191,192,193,194,195,196,197,198,199,200,201,202,203,204,205,206,207,216,217,218,219]. In 2003, the crystal structure of the synthetic non-heme Fe(IV)=O species [181] was first discovered, providing spectroscopic results for the characterizations of these species. In the same year, the first characterization of the so-called TauD was also performed, opening the biochemistry of the non-heme iron-oxo species [182]. After these discoveries, a chemical synthesis of the non-heme Fe(IV)=O model complexes have been performed to elucidate the mechanism of the hydroxylation reactions by Fe(IV)=O [183,187,190,192,194,200,202]. The mono-oxygenation reactions by TauD and related biological systems have also been investigated to elucidate key roles of the non-heme Fe(IV)=O intermediates for biological functions [184,185,189,191].

Judging from the computational results [186,193,197,198,199,201], hydrogen radical abstraction (HRA) mechanisms are concluded in many cases, indicating that both the π-SOMO of Fe=O-HOMO (H-C bonding orbital) and π-SOMO of Fe=O-LUMO (H-C anti-bonding orbital*) interactions are operative in accordance with the essentially neutral charge at the hydrogen site in the transition states for HRA reactions. However, the latter orbital interaction is often neglected for simplified illustrations of the HRA reactions. 

On the other hand, the ET-PT mechanism is really feasible for strong electron-donating substrates, such as H-N bond of amines [21]. The UB3LYP computations [186,193,197,198,199,201] have elucidated that the spin crossovers between different spin states in Equations (36)–(39) often occur along the reaction pathways for the HRA reactions, indicating that ferromagnetic effective exchange interactions among local spins play important roles for the stabilization of transition structures and reaction intermediates. This entails difficult theoretical problems to depict the accurate potential curves for HRA reactions [204,205,206,207,218,219]. These interesting results are revisited later. 

### 4.11. Quantum Spin Hamiltonian Models for Binuclear Transition Metal Oxides

In this section, early ab initio computations of the binuclear transition metal complexes [27,81,82,220] have been revisited in relation to the EPR spectroscopy and hole doping on the oxygen site. The following exchange-coupled binuclear complexes have been involved in metalloenzymes [17,221,222,223,224,225,226]: (1) binuclear copper oxide (CuO_2_Cu) in hemocyanin and binuclear iron oxide (FeXFe) (X = OH, O) of hemerythrin for molecular oxygen carriers, (2) binuclear iron and copper oxides (MOM) (M = Fe and Cu) of methane monooxygenase, (3) binuclear Mn oxide (MnOMn) of Mn-catalase for molecular oxygen carriers. In the early 1980s, the exchange-coupled transition metal complexes were synthesized as model complexes of these redox-active metalloenzymes [17,217,220,221]. The paramagnetic susceptibility and EPR experiments revealed the effective exchange integrals (*J*) for binuclear transition metal complexes at that time. The isolobal and isospin analogy between oxygenated dipoles and transition metal μ-oxo dimer was our guiding principle to elucidate the electronic and spin state of the M-O-M systems [27,81].

•X–O–X• (X = O, NH, CH_2_) ↔ •Y–O–Z• (Y, Z = Mn, Fe, Cu…) (40)

**Figure 19:**
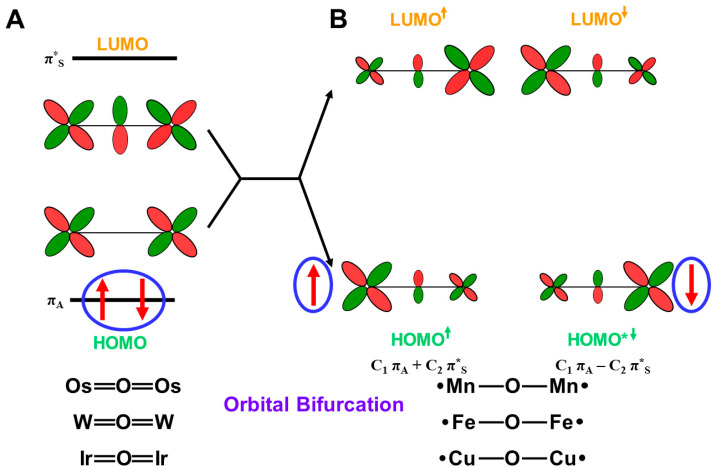
(**A**) The closed-shell HOMO (non-bonding) and LUMO (anti-bonding) for M=O=M systems and (**B**) The spin polarized molecular orbitals for the •M-O-M• systems obtained by the HOMO-LUMO mixing. The BS orbitals are mainly localized on the left (L) and right (R) M-sites, indicating the 1,3-diradical characters. The effective exchange interactions (*J*) between the BS orbitals localized on the L and R sites are given in Table 7 [27]. Several M=O=M and •M-O-M• systems are also depicted for explanations. The up and down arrows are denoted the up and down spins of electron, respectively.

Both systems are essentially regarded as three-center four-electron [4e, 3o] systems. Therefore, the HOMO becomes the antisymmetric non-bonding orbital, as shown in Figure 19. On the other hand, the LUMO is the symmetric anti-bonding MO. The [4e, 3o] π-bonds are stable for 4d and 5d metals, as shown in Figure 19, indicating the large HOMO-LUMO gap. On the other hand, the orbital energy gap between the HOMO-LUMO is small for 1, 3-diradical and M–O–M systems (M = 3d transition metals), indicating the spontaneous HOMO-LUMO mixing that entails the broken-symmetry orbitals mainly localized on the left and right metal sites, respectively, as shown in Figure 19. Therefore, the Heisenberg model is applicable for the 3d-metal systems with strong 1,3-metal diradical character •M–O–M•. Thus, the isolobal and isospin analogy in Equation (40) is applicable to the [4e, 3o] systems [27]. The BS UHF calculations were also performed for binuclear complexes for confirmation of the analogy. To this end, the *J* values of these transition metal complexes were obtained by using the computational scheme after spin projection in Equation (34) [25,26,27,28,29]. The computational results are summarized in Table 7. 

The *J*-values for the Cr(III)-O-Cr(III) complex were calculated to be largely negative in sign, indicating the strong antiferromagnetic (AF) interaction. However, the calculated *J*-value was −187 cm^−1^ for the NH_3_Cr(III)-O-Cr(III)NH_3_, indicating the suppression of the super-exchange interaction with the coordination of NH_3_ ligand [27]. It was compatible with the observed *J*-value for the (NH_3_)_5_Cr(III)-O-Cr(III)(NH_3_)_5_ [25], indicating the applicability of the computational procedure for elucidation of the nature of the exchange-coupled binuclear complexes. The *J*-values calculated for the Mn(II)-O-Mn(II) indicated the spin crossover from the antiferromagnetic (AF) and ferromagnetic (F) state with the increase in the Mn-O distance [27]. The calculated *J*-value for NH_3_Mn(III)-O-Mn(III)NH_3_ was −60 cm^−1^, indicating the AF interaction is consistent with the magnetic behaviors of many Mn oxides [80]. However, it exhibited the spin crossover from AF to the ferromagnetic (F) state in the elongated Mn-Mn distance. The *J*-values calculated for the Fe(III)-O-Fe(III) also indicated the spin crossover from the AF and F state with the increase in the Fe-O distance [17]. The linear Ni(II)-O-Ni(II) unit exhibited the AF interaction.

The linear (180 degree) Cu(X)-(μ-O)-Cu(X) (X = II, III) unit indicated the large negative value, indicating the extremely strong AF interaction [27]. This large |*J*| was used to estimate the transition temperature of the high-*T*_c_ superconductivity of hole doped calcium copper oxide by partial substitution of Ca(II) with La(III), indicating the hole doping via the conversion of Cu(II)-O^2−^-Cu(III) into Cu(II)-O•^1−^-Cu(II) [220]. The oxyl-radical site for Cu(II)-O•^1−^-Cu(II) was responsible for the oxygenation reaction [27,221]. The hole doping at the oxygen site of the high-valent M-O•^1−^-M systems (M = Fe, Cu, etc.) [25,27,29] was not only one of the guiding principles for understanding of oxygenation reactions [17,221,224], but also the working hypothesis for theoretical understanding of unusual properties, such as hole-doped superconductivity [220]. Thus, the concept of the hole-doping in the transition metal oxides [27,190] was one of the guiding principles for our theoretical investigations of strongly correlated electron systems (SCES), such as hole-doped copper oxides [27,190]. 

The *J*-values calculated for the Cu(II)(μ-OH)_2_Cu(II) complex indicated the strong Cu-O-Cu angle (*θ*) dependence, as shown in Table 7 [28]. The *J*-values were considered to be positive for the Cu(II)(X)_2_Cu(II) (X = O^2−^, OH^−^) complex with smaller angles (<100 degree) because of the contribution of the charge-transfer configuration Cu(I)-O(^−^•)-Cu(II), where the orthogonal 2p-orbital and 3d-orbital interaction was feasible for the ferromagnetic (F) super-exchange interaction. The calculated *J*-values were indeed positive for the Cu(II)(OH)_2_Cu(II) complex with smaller angles (<100 degree). On the other hand, the calculated *J*-values are negative for the Cu(II)(OH)_2_Cu(II) complex with larger angles (>110 degree) because of contribution of the charge-transfer configuration Cu(I)-O(^−^•)-Cu(II) where the non-zero 2p-orbital and 3d-orbital interaction is feasible for the antiferromagnetic (AF) super-exchange interaction. 

The same mechanism was found to be operative for the Cu(II)(OH)_2_Ni(II) complexes. The hole doping on the oxygen site of the high-valent M(X)-(O^2−^)-M(X) unit is often feasible, providing the active oxyl-radical site; M(X)-(O^−^•)-M(X). The oxygen-radical site of the Fe(IV)-(O^−^•)-Fe(III) cluster is indeed responding for methane monooxygenase [17,223,224]. Thus, hole doping on the oxygen site is very important for both material sciences [220] and radical reactions [25,26,27,28,29].

The *J*-values for the Fe(III)S_2_Fe(III) unit [25,97] were calculated to be antiferromagnetic (AF) in consistent with the experiments [34,35,36]. Indeed, ferredoxin plays an important role in the electron transfer in P450 enzyme as mentioned in the introduction [36]. The early BS calculations combined with the Heisenberg model were found to be useful and practical for the theoretical elucidation of the binuclear transition metal complexes [27], which were regarded as model complexes for the active sites of several metalloenzymes [221,222,223,224,225,226]. Recently, BS hybrid DFT methods, such as UB3LYP, have been conveniently used for the elucidation and computation of the sign and magnitude of *J*-values for multi-nuclear transition metal complexes involved in metalloenzymes [80].

## 5. Theoretical Investigations of Mono-Oxygenation Reactions by P450 and Related Species

### 5.1. Natural Orbital Analysis of the BS Solutions and Chemical Indices of the Intermediates 

**Figure 20:**
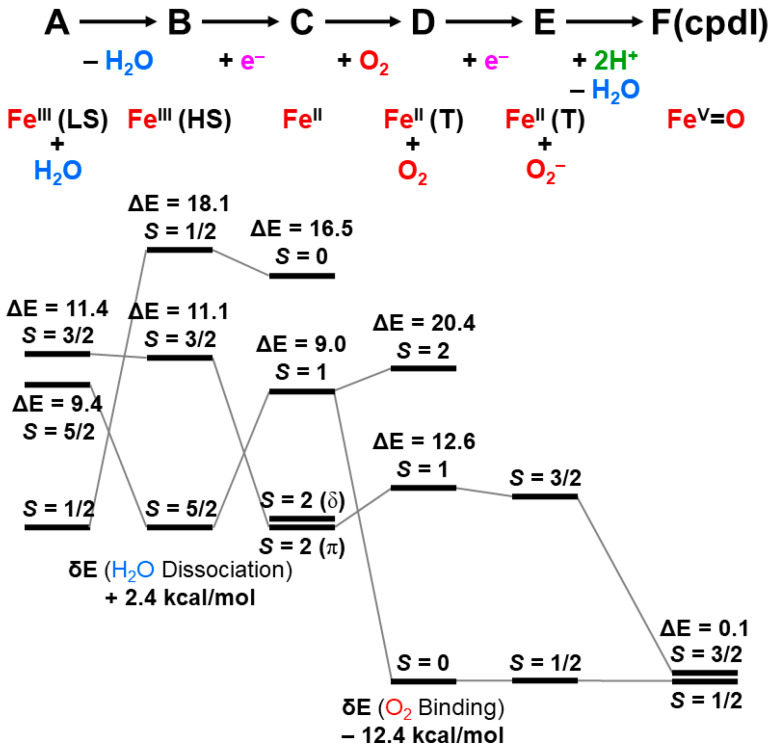
The energy levels of the ground and lower-lying excited spin states of the key intermediates (**A**–**F**) in the P450 formation cycle by UB3LYP methods [143]. The detailed discussions are given in the text.

**Figure 21:**
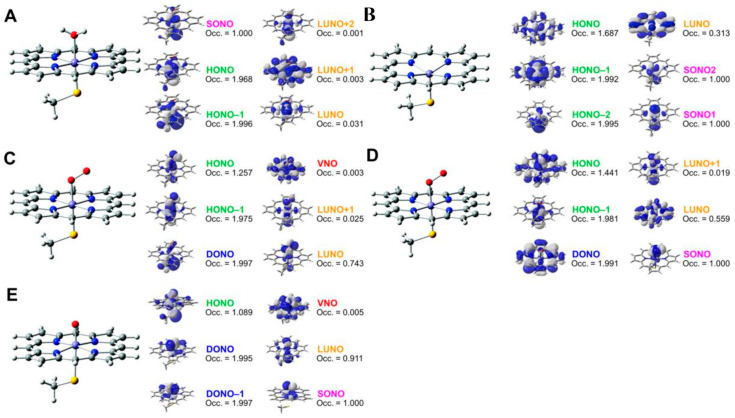
The natural orbitals and their occupation numbers of the (**A**–**E**) intermediates in the P450 formation cycle by the UB3LYP method [143,144]). Detailed discussions are given in text.

In this section, the natures of the chemical bonds of all the intermediates in the P450 cycle have been revisited. The BS hybrid DFT methods are nowadays handy and practical tools for theoretical investigations of large transition metal complexes. Extended BS computations, including full geometry optimizations [142,143], were performed to confirm early BS ab initio computational results for elucidation and understanding of the nature of chemical bonds of iron-peroxide and iron–oxo intermediates in the P450 reaction cycle in Figure 1. Both DZ and TZ basis sets [227,228] were used for UB3LYP computations by available program packages [229,230]. The extended BS UB3LYP computations elucidated the spin state levels of the key intermediates (**A**~**F** in Figure 1), as shown in Figure 20. The full geometry optimizations were also performed to elucidate the geometric structures of these intermediates, as shown in Figure 21. The charge and spin densities of the optimized geometric structures are summarized in Table 8. The natural orbital analysis of the BS solutions was also performed to elucidate natural orbitals (UNO) and their occupation numbers (*n*_i_). The chemical indices were also obtained by using the relations; *n*_i_(HOMO) = 1 + T_i_ and *n*_i_*(LUMO) = 1 − T_i_, as shown in Table 9. 

The optimized Fe-S and Fe-O(H_2_) distances were 2.04 and 2.10 Å, respectively, for the intermediate **A** in Figure 21 [143,144]. The spin density on the Fe(III) was 1.02, in accordance with the low-spin (*S* = 1/2) state, as shown in Table 8. The δ_1_ (δ_xy_) and π_1_ (*dπ_yz_*) were doubly occupied because of porphyrin and thiolate ligands. On the other hand, π_2_ (*dπ_xz_*) was singly occupied (SOMO) for **A** in Figure 21. The energy levels of **B** are shown in Figure 20, indicating the high-spin sextet (S_extet_) ground state (*S* = 5/2). The energy gap between the *S*_extet_ and quartet (Q_uartet_) states was calculated to be 11.1 kcal/mol, indicating *J*_ab_(S_extet_-Q_uartet_) = 11.1/4~2.8 kcal/mol. The energy gap between Q_uintet_ and doublet (D) states was 7.0 kcal/mol, providing *J*_ab_(Q_uartet_-D) =7.0/2 = 3.5 kcal/mol. Therefore, the average *J*_ab_(S_extet_-D) is about 3 kcal/mol for **B**, indicating the ferromagnetic exchange interaction within the Fe(III) ion. The optimized Fe-S (Fe-Por) distances for **C** were 2.44 (−0.50), 2.40 (−0.28), and 2.33 (−0.25) Å, respectively, for the ground quintet (Q_uintet_), excited triplet (T), and singlet (S) states, respectively. The Fe(III) ion bellows the plane of porphyrin. The spin densities on the Fe ion were 3.9, 2.9, and 0.2 for these states, respectively, as shown in Table 8. From the energy levels in Figure 20, the *J*_ab_(Q_uintet_-T) and *J*_ab_(T-S) values were 2.2 and 3.8 kcal/mol, respectively, for **C**. The δ_1_ and π_1_ orbitals were doubly occupied in the triplet state (*S* = 1) of **C**. On the other hand, π_2_ and dσ orbitals are SOMOs for **C,** as shown in C2 of Figure 21. 

The optimized Fe-S, Fe-O_1_, and O_1_-O_2_ distances were 2.38 (2.45), 1.94 (1.98), and 1.37 (1.38) Å, respectively, for the ground singlet (doublet) state of (**D**,**E**) in Figure 21. The spin densities on the S, Fe, O_1_, and O_2_ in Table 8 were 0.06 (−0.02), 1.08 (0.93), −0.66 (−0.38), and −0.39 (−0.59), respectively, mainly indicating the exchange coupled configuration between the doublet Fe(III) ion and superoxide anion. The energy gap between the triplet and quintet states of **D** was calculated to be −7.8 kcal/mol, indicating *J*_ab_(T-Q_uintet_) = −7.8/4 ~ −2.0 kcal/mol. The energy gap between the singlet and triplet states of **D** was −12.6 kcal/mol, providing *J*_ab_(S-T) = −12.6/2 = −6.3 kcal/mol. Therefore, the average *J*_ab_(S-Q_uintet_) is about −3 kcal/mol for **D**, indicating the AF exchange interaction, as shown in Figure 20. The large negative *J*_ab_(S-T) value implies the EPR silent singlet ground state of **D** [39]. A similar situation was also expected for Cu(II)-O-O^−^ systems with a large negative *J*_ab_(S-T) value [221].

The energy gap between the doublet and quartet states of **E** was −12.1 kcal/mol, providing *J*_ab_(D-Q_uartet_) = −12.1/2 = −6.1 kcal/mol. The occupation numbers of the MOs (UNO) for SH anion group and Fe-O-O σ-bond of (**D**,**E**) were about 2.0, indicating the closed-shell character. On the other hand, the Fe-O-O π_2_-bond was largely spin polarized (*T*_i_ = 0.26), indicating the large diradical character (*Y* = 52%), as shown in Table 9, confirming the early results [26,29]. Therefore, a PorLFe(III)OO intermediate may undergo the radical addition to the C=C double bond in some cases, as pointed out in Figure 2 [29,116]. On the other hand, π_1_ is singly occupied (SOMO) for **E** in Figure 20. The π_2_ was largely delocalized over porphyrin group for **E**, indicating the non-negligible SP effect, as shown in Table 9. Therefore, the electronic structure of the O_2_ part of **E** is rather similar to superoxide anion instead of oxygen dianion.

The optimized Fe-S and Fe-O_1_ distances were 2.38 and 1.94 Å, respectively, for the ground doublet state of **F** in Figure 21. The spin densities on the S, Fe, and O_1_ in Table 8 were −0.81, 1.19, and 0.91, respectively, mainly indicating the electron transfer from the thiolate anion (^−^SH) instead of the porphyrin (^2^Cpd I_a_) type configuration in Figure 17. The alkyl thiolate anion (R-S^−^) instead of HS^–^ is necessary for the generation of ^2^Cpd I_a_ in Figure 17. The energy gap between the doublet and quartet states of **F** was calculated to be −0.1 kcal/mol, indicating *J*_ab_(S-T) = −0.1/2 ~ −0.05 kcal/mol (−175 cm^−1^). Therefore, the magnitude of the calculated *J*_ab_(S-T) of **F** (with the SH anion) is ten times larger than the observed value for ^2^Cpd I_a_ in Table 6. The π_1_ is singly occupied (SOMO) for **F,** as shown in F2 of Figure 21. On the other hand, the π_2_-bond is largely spin-polarized (*T*_i_ = 0.09), indicating the large 1,3-diradical character (*Y* = 82%; ↓•S-Fe-O↑), as shown in Table 9. The extended BS computational results in Figure 20; Figure 21 [143,144] have supported our early computational results [25,26,27,28,81,82] for the reaction cycle of P450 enzymes in Figure 1. Several chemical indices, such as diradical character, are also found to be useful for elucidation of the nature of chemical bonds of **D**, **E,** and **F** in Table 9.

The UHDFT computations [144] were performed for PorLFe=O with different axial ligands in Figure 17 to elucidate the vibrational frequencies of the Fe=O bonds [63] and Mossbauer parameters for comparison with the observed results by Cpd I and Cpd II. The calculated frequencies for Fe(IV)=O of Cpd I (Cpd II) were 912 (834), 851 (816), 853 (873), 896 (800), and 857 (863) cm^−1^, respectively, for **1**, **2a**, **2b**, **3**, and **4** in Figure 17. The calculated ΔEq (δ) values were 0.71 (0.20), 0.13 (0.16), 0.23 (0.20), 0.99 (0.14), 1.31 (0.31), and 1.29 (0.16) for **1**, **2a**, **2b**, **3**, **4**, and chloroperoxidase, respectively. The observed value for P450(**3**) was 1.02(0.16) [21]. Thus, UB3LYP was reliable enough for computations of spectroscopic parameters of the Fe(IV)=O compounds [21,27]. Thus, UHDFT methods are applicable to elucidate the structure and bonding of the intermediates in the P450 reaction cycle in Figure 1 and Figure 3.

### 5.2. Confirmations of the Models of Mono-Oxygenation Reactions by Ab Initio Calculations 

Here, the atomic oxygen model for mono-oxygenations by Fe=O have been revisited on the basis of UHDFT computations of transition structures. The atomic oxygen (O) models for oxygen insertion and radical rebound mechanisms [27,81] were proposed for mono-oxygenations by P450 enzymes and related enzymes, as illustrated in Figure 10. It is well-known that atomic oxygen undergoes insertion reaction into hydrocarbons in the excited singlet state, whereas triplet atomic oxygen undergoes hydrogen radical abstraction (HRA) [231,232]. On the other hand, introductions of heteroatoms and polar substituents are necessary for ET and ET-PT reactions [27,28,94,233,234,235].

**Figure 22:**
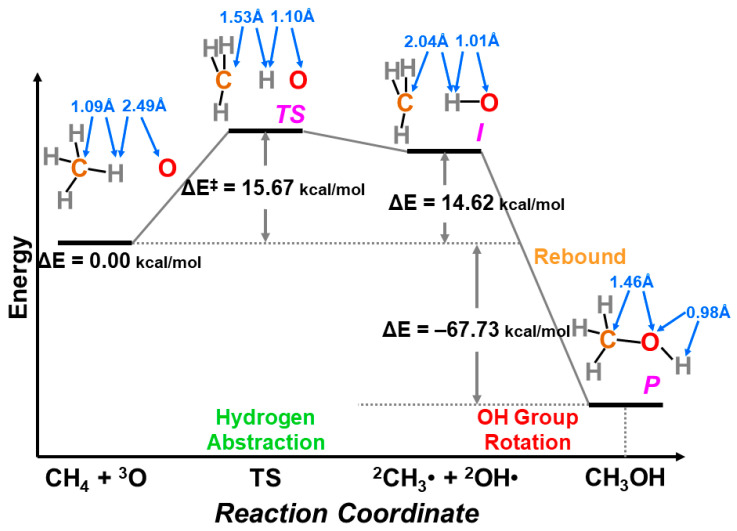
The optimized geometric parameters for the reactant, transition structure and intermediate and product, and the potential energy diagram for hydrogen abstraction reaction of methane by triplet atomic oxygen (^3^O), supporting the triplet O-model for mono-oxygenation by Fe(IV)=O in Figure 10B [143,144].

Ab initio BS computations [143] were performed to locate the transition structure (TS) for hydrogen radical abstraction reaction from methane (**I**), as shown in Figure 22.

H_3_-C-H_B_ + ↑•O•↑ → H_3_-C•↑…H_B_O•↑ → SI → H_3_-C•↑…↓•OH_B_ → H_3_-C-OH_B_ (41)

where SI denotes the spin inversion. The optimized C-H_B_ and H_B_-O distances at TS are 1.51 and 1.10 Å, respectively. The activation barrier for the hydrogen abstraction reaction of **I** by triplet atomic oxygen (^3^P) was about 16 kcal/mole, providing the triplet diradical intermediate with the corresponding distances of 2.04 and 1.01 Å followed by SI to afford singlet diradical for facile radical coupling, as shown in Equation (41) and in Figure 22 [143]. On the other hand, the activation barrier for the singlet oxygen insertion was about 2 kcal/mol [143], supporting the two-state model [27,209,210,211,212,213].

**Figure 23:**
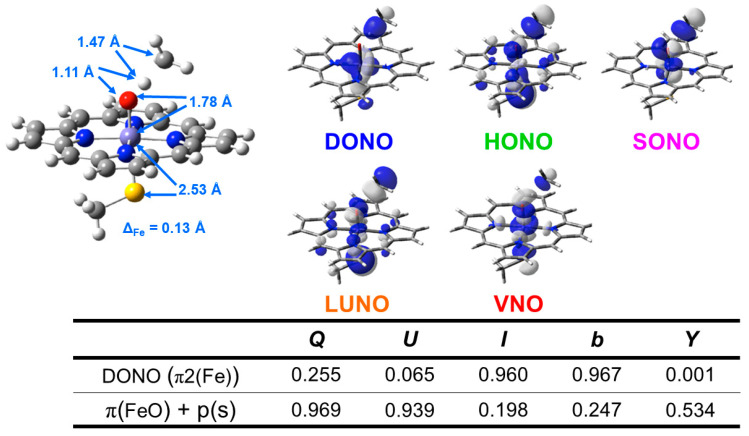
The geometric parameters for the transition structure of the hydrogen abstraction from methane by Por(+•)(S-CH_3_)^3^Fe(IV)=O. The doubly occupied (DONO), vacant (VNO), singly occupied (SONO), highest-occupied (HONO), and lowest unoccupied (LUNO) natural orbitals are depicted to elucidate the DONO-VNO and HONO-LUNO mixings. The radical character (*Y*) is small for DONO, indicating the almost no DONO-VNO mixing. On the other hand, the large *Y* value (53%) and small effective bond order (*b*) for the π-orbital indicate the hydrogen abstraction reaction (HRA) [143,144].

For the elucidation of the isolobal and isospin analogy in Figure 10, we performed locations of the three transition structures (^4^TS_a_, ^2^TS_a_, and ^2^TS_b_) for hydrogen abstraction reaction from **I** by ^4^Cpd I_a_, ^2^Cpd I_a_, and ^2^Cpd I_b_ in Equations (36a)–(36c), as shown in Figure 23 [143,144,145,146]. The UB3LYP with the LACVP++** (Fe) and 6-31++G** (others) were used for this purpose [227,228]. The calculated activation barriers after the zero-point correction energy for these three configurations were found to be 21.3, 23.1, and 25.4 kcal/mol, respectively. On the other hand, the activation barriers for ^4^CpdI and ^2^CpdI_a_ are 22.81 (22.91) and 22.24 (22.34) kcal/mol, by UB3LYP/B1(DZ) calculations by Shaik group [147], where the corresponding values by B2(TZ) are given in parentheses. The calculated ΔE^‡^ value was calculated to be over 21 kcal/mol for methane (**I**) by the compound I (Cpd I). 

The hybrid DFT (UB3LYP) computational results indicated that the methane mono-oxygenation by Cpd I was difficult in accordance with available experiments [17,224], indicating the necessity of guiding principles for the reduction of the activation barriers (see Section 5.5 later). Nevertheless, the triplet atomic oxygen model [27,81] works well for qualitative understanding of hydrogen radical abstraction (HRA) reactions by Cpd I. Indeed, the chemical indices added in Figure 23 are consistent with the radical abstraction mechanism. The binuclear Fe-oxide (Fe-O-Fe), the so-called methane monooxygenase, was essential for mono-oxygenation of **I** in biological systems [17,224,225]. Alternately, a different binuclear Fe-oxide (Fe(III)-O-Fe(V)=O (Fe(III)-O-Fe(IV)-O•) intermediate might be formed for the methane mono-oxygenation [226]. Thus, the extended UHDFT computations have supported our early triplet atomic oxygen (O) model for the high-valent Fe(X)=O (X = IV, V) of Cpd I and molecular oxygen (O=O) model for Cpd II.

### 5.3. The Oxyl-Radical Character of the High-Valent Fe(V)=O Bonds 

Here, the calculated activation barriers for mono-oxygenation by the heme-type Fe(V)=O bond with the significant oxyl-radical character [27,81] have been re-examined in relation to recent computational results. Past decades, mono-oxygenation reactions by the high-valent iron-oxo compounds Cpd I have been investigated by various broken-symmetry (BS) UDFT computations [147,148,149,150,151,152,153,154,155,156,157,158], indicating that the activation energies for hydoxylations by Cpd I are reduced to be 15~20 kcal/mol for alkanes. The computational results for mono-oxygenations by ^2^Cpd I_a_ and ^4^Cpd I_a_ have already been discussed in several review articles [83,84,85,86,87,88,89]. In this section, we only examine the spin polarization (SP) effect of the high-valent Fe(V)=O core in relation to our early proposal of the metal-oxyl-radical character of the high-valent Fe=O bonds [25,26,27,28,29]. The detailed computational results [147,148,149,150,151,152,153,154,155,156,157,158] were already published for key substrates molecules for the following mono-oxygenations: ethane (**IV**), *i*-propane (**V**), *n*-propane (**VI**), propene (**VII**), methylphenyl cyclopropane (**VIII**), isopropylphenyl cyclopropane (**IX**), dimethyl aniline (**X**), and toluene (**XI**). Table 10 summarizes the populations of spin densities, x-values (see Equation (42)), and ΔE^‡^ values for the transition structures for hydrogen radical abstraction (HRA) reactions revealed by the DFT calculations [147,148,149,150,151,152,153,154,155,156,157,158]. The transition structure is responsible for the bond breakings of the Fe=O double bond (•Fe-O•) and C-H single bond (•H-C•), followed by the coupling between the O• and •H sites to generate metal 1,4-diradical (•Fe-O-H C•) [143,144,145,146]. The TS geometry is determined with a subtle balance between these bond-breaking energies [174]. Therefore, we can define the simple geometrical parameter to express early or late TS [143,144] as follows:

x = R(H-C)/(R(H-C) + R(Fe-O)) = R_2_/(R_1_+R_2_) = R_2_/R_t_ (42)

where R(X-Y) denotes the optimized X-Y length at TS. Small x-values mean early TS in this definition. Certainly, the x-value was 0.38 (38%) for the insertion reaction of the singlet atomic oxygen (^1^O(^1^D)). On the other hand, the x-value was 0.58 (58%) for abstraction reactions by ^3^O.

The activation parameters for mono-oxygenations by the UB3LYP computations [147,148,149,150,151,152,153,154,155,156,157,158] were about 15~19 kcal/mol in accordance with the hydrogen radical abstraction (HRA) mechanism with the large kinetic isotope effect [83,84,85,86,87,88,89,90]. From Table 10, the spin densities on the Fe ion and carbon site were about 2.0 and –0.5, respectively, in accordance with predictions based on the oxyl-radical character ^2^↑↑••Fe(IV)-O•↓ of the high-valent Fe(V)=O species, as illustrated in Figure 10 and Figure 16. In fact, the negative spin density was populated on the generated alkyl radical site (↓•) of Alk. Thus, early prediction of the oxyl-radical character by the HOMO-LUMO mixing of the high-valent 3d metal-oxo bonds [27] is consistent with the available computational results [147,148,149,150,151,152,153,154,155,156,157,158].

From Table 10, the activation barrier for dimethyl aniline was calculated to be small (about 7 kcal/mol) when compared with the standard values (15~19 kcal/mol) for HRA reactions, indicating the electron transfer-coupled proton transfer (ET-PT) process for the mono-oxygenation of strong electron-donating substrates in accordance with the four different mechanisms in Figure 10. Therefore, the rate-determining step for *N*-hydroxylation was found to be the late rebound step for the ET-PT process [21]. The ET process was also important for singlet oxygen reactions of electron rich olefins, as shown in Figure 10 [25,81]. Thus, the mechanisms of the mono-oxygenations by P450 are variable, depending on the types of substrates under examination [83,84,85,86,87,88,89,90]. 

### 5.4. Mono-Oxygenation by the Non-Heme High-Valent Fe(IV)=O Bonds 

Here, the scope and applicability of early atomic oxygen models [27,29] have been examined in relation to UHDFT computations of non-heme Fe(IV)=O bonds [177,178,179,180,181,182,183,184,185,186,187,188,189,190,191,192,193,194,195,196,197,198,199,200,201,202,203,204,205,206,207,216,217,218,219]. In the 1980s, the radical reactivity of the high-valent transition metal oxo compounds was discovered [42,45]. Therefore, we performed the ab initio UHF computations of M=O species in Table 2 to elucidate their radical reactivity. In fact, the 1,4-diradical mechanism was found to be more favorable than the concerted mechanisms for M=O (M = Fe, Mn), as shown in Figure 12 [27]. In past decades, mono-oxygenations by the non-heme compounds [177,178,179,180,181,182,183,184,185,186,187,188,189,190,191,192,193,194,195,196,197,198,199,200,201,202,203,204,205,206,207,216,217,218,219] were investigated by both experimental and theoretical methods. The X-ray diffraction of the I-heme KNp4(ACN)Fe(IV)=O crystal with the O*_h_* ligand field in Figure 8A was performed, elucidating that Fe-O distance is 1.646 Å [181], which is a little longer than the optimized value (1.619 Å) for the naked ^5^Fe(IV)=O in Table 2 [78]. On the other hand, the optimized Fe-O distances by UB3LYP [144] were calculated to be 1.641, 1.657, and 1.646 Å, respectively, for the catalase (CT), peroxidase (PO), and P450 models in Figure 17. KNp4(ACN)Fe(IV)=O was found to be the ground triplet species in accordance with the energy levels of the Oh ligand field in Figure 8A [27,181]. The Fe(IV)=O stretch for KNp4(ACN)Fe(IV)=O was observed at 834 cm^−1^ by the FTIR experiments [181]. 

Hybrid DFT computations have been performed for the elucidation of the mono-oxygenation reactions via Fe(IV)=O of non-heme enzymes by several groups [186,188]. The energy gaps between the singlet (^1^Δ_πxπy_) (No. 16 in Table 5) and triplet (^3^Π_πxπy_) (No. 13 in Table 5) configurations at the reactant (R) stage are about 8~12 kcal/mole [186,188]. Therefore, the energy gaps after spin projection by using Equations (34) and (35) become 16~24 kcal/mol, like in the case of molecular oxygen, which exhibits the variation of the gap from 11 to 22 kcal/mol after spin projection [81]. Thus, the spin contamination effect is not negligible for singlet Fe(IV)=O [145]. On the other hand, the spin projection effect for the low-spin singlet state is relatively small at the transition structure (TS) and radical (I) intermediate because of the small ST gaps. 

Hirao and Shaik et al., performed extended UB3LYP computations for six non-heme Fe(IV)=O compounds to locate the transition structures and the potential curves for mono-oxygenations of organic molecules [186]. Table 11 summarizes the spin density populations of reactant (R), transition structure (TS) and reaction intermediate, and activation barriers (ΔE^#^) for [(Bn-TPEN) Fe(IV)=O]^2+^ [186]. From Table 11, the spin densities on the Fe, O, and C sites of a singlet state (2*S* + 1 = 1) of the reactant complex are 0.54, −0.51, and 0.0, respectively. On the other hand, they are 0.83 (0.90), −0.30 (0.09), and −0.48 (−0.94) for TS, where the corresponding values for the intermediate are given in parentheses. These results are wholly compatible with the singlet-type reaction scheme by Fe(IV)=O (^1^Δ_πxπy_) in Equation (38a) like the singlet O (^1^Δ_xy_) model [81]. The calculated activation barrier for this excited radical reaction is about 14 (9.4) kcal/mol in the excited singlet surface without (with) solvation effect.

The spin densities on the Fe, O, and C sites of the triplet state of reactant are 1.11, 0.96, and 0.0, respectively. They are 0.96 (0.91), 0.64 (0.22), and 0.49 (0.93) for TS (Intermediate), respectively. The calculated activation barriers without (with) solvation effect were 8.9 (12.5) kcal/mol in the triplet surface. These results are wholly compatible with the ground triplet radical reaction scheme by Fe(IV)=O (^3^Σ_πxπy_) (*S* = 1) in Equation (38b) like the triplet O (^3^P) model [27,81]. The energy gap between the ground triplet (*S* = 1) and singlet (*S* = 0) states was about 9 kcal/mol at the reactant (R) state. Therefore, the singlet state is destabilized after spin projection [27,81], providing the large singlet (S)-triplet (T) energy gap (about 12~14 kcal/mol). The triplet-singlet energy gap is about 5 kcal/mol at the TS, indicating that it becomes about 8 kcal/mol after spin projection. Therefore, the activation barrier for hydroxylation for the projected singlet state is estimated to be 3~5 kcal/mole, indicating a significant reduction. 

The spin densities on the Fe, O, and C sites of the excited quintet state (*S* = 2) of the reactant are 2.98, 0.73, and 0.0, respectively, indicating the spin delocalization (0.29) at the ligand (L) site. They are 3.71 (3.99), 0.24 (0.23), and −0.34 (−0.85) for the first TS, respectively, where the corresponding values for the intermediate are given in parentheses. The corresponding spin density at the L site is 0.39 (0.48), indicating that a positive spin is delocalized over the O and L sites, which undergoes radical coupling with the alkyl radical. The activation barriers without and with solvation effects were calculated to be 0.22 and 7.1 kcal/mol, respectively, in the quintet surface. The polar solvent effect was remarkable in the quintet state [186]. Nevertheless, the spin crossover from the triplet to the quintet state took place along the reaction coordinate [186]. Available computational results indeed indicated the greater reactivity of the quintet state (*S* = 2) than that of the triplet state for non-heme Fe(IV)=O species with the Oh ligand field in the transition state (TS) region [193,195,197,199,201].

The very small activation barrier for hydrogen radical abstraction in the quintet state is in accordance with the ET-PT process in Equation (37d). The negative spin density on the carbon site is −0.85 for the intermediate (I), indicating the intermediate overlap *T*_i_ = 0.53 and *Y* = 18% for radical pair: •(Fe(III)-O-H)…(•R), where the spin densities were calculated to be about 0.85 on Fe and –0.85 on alkyl radical, respectively. Judging from these chemical indices, the radical abstraction process is operative for singlet and triplet states. On the other hand, the ET-PT character in Equation (37d) is contributable to the stabilization of TS in the quintet state even by the solvation model [181]. The spin polarization (SP) effect in Figure 18 is also partly contributable to the reaction. However, beyond HDFT computations such as CASPT2/CC (see later) are necessary for elucidation of the relative contributions of the ET and SP effects. Interestingly, the activation barrier from the initial triplet state to the quintet TS for hydrogen abstraction is the smallest (ΔE^#^ = 7.1 kcal/mol) after the spin crossover. Hirao et al. [193] considered three different scenarios for the spin transitions. The kinetic isotope effect was calculated to be small (4–6) as expected for ET-PT theoretically against the large experimental results [190,193]. 

### 5.5. Reduction of the Activation Barriers for Mono-Oxygenations 

Here, the electron transfer (ET)-coupled with the proton transfer (PT) is examined in relation to the reduction of the activation barriers for mono-oxygenations. The calculated low activation energy (about 7 kcal/mol) for the mono-oxygenation of the amine (**X**) by heme-type iron-oxo catalysts in Table 10 indicates that the ET-PT is one of the practical procedures for the reduction of the activation barrier, as shown in Equation (37a,b). The extremely low activation barrier (0.2 kcal/mol) for the ET-PT process in the quintet (*S* = 2) state of [(Bn-TPEN)Fe(IV)=O]^2+^ in the gas phase [181] indicates that the hydrophobic environment is also important for its reduction in the non-heme iron-oxo catalysts, as shown in Equation (37c,d). As discussed previously [80], Val185 plays an important role in the formation of the hydrophobic environment for the O-O bond formation in PSII. 

Soluble methane monooxygenase (sMMO) converts methane to methanol under ambient conditions [17,224,236,237]. The diiron active site is buried within a hydroxylase (MMOH) hydrophobic protein environment, undergoing the mono-oxygenation of CH_4_ with the bond dissociation energy (BDE) of 105 kcal/mol within the caged reaction field. Three hydrophobic cavities also link the diiron center to the protein exterior, indicating the characteristic reaction field of sMMO. Very recently, Fujisaki et al. [238] have reported the mono-oxygenation of CH_4_ by the artificial iron-oxo catalysts, [Fe(IV)=O(PY_4_Cl_2_Blm)]^2+^ with the triplet ground state (*S* = 1). This interesting catalyst consisted of the hexagonal ligand field with four bulky ligands (three-linked benzonitrile (3-BN)), exhibiting the hydrophobic reaction field like sMMO [237].

According to the DFT computations by Fujisaki et al. [238], the activation barrier for the first hydrogen transfer step was about 19 kcal/mole under the assumption of the *S* = 1 state. The calculated activation barrier is consistent with the hydrogen radical abstraction (HRA) from CH_4_ by present and other computations [143]. This, in turn, indicates the possibility of the ET-PT mechanism for the reduction of the activation barrier; for example, 8.7 (=8.9 − 0.2) and 5.4 (=12.5 − 7.1) kcal/mole for the hydrophobic and hydrophilic environments, respectively [181]. Therefore, the activation barrier by the ET-PT mechanism is estimated to be about 12~16 kcal/mol even for CH_4_. Thus, we have the following guiding principle for the ET-PT process for mono-oxygenation by iron-oxo catalysts: (1) construction of hydrophobic reaction field by bulky ligands, (2) introduction of polar substituents for reduction of the ET excitation energy, and (3) quantum effect of proton transfer (PT).

Such molecular environments may be important for the native mono-oxygenation by P450 enzyme [21]. The detailed DFT computations by Isobe et al. [146] have elucidated that the high-valent Fe(V)=O bond is generated in the native reaction field (see Figure 3), undergoing a facile mono-oxygenation of H-C bonds. The theoretical reaction profile is consistent with the ET-PT process. This indicates that methane mono-oxygenations may be feasible by the direct evolution [239] and chemical modification (decoy molecules) [214,240] of P450 enzymes. 

## 6. The Computational Results for Beyond HDFT Results

### 6.1. Beyond BS Computations

In this section, the scope and reactivity of the computational results by the BS methods have been revisited in relation to the beyond BS results [103,104]. In the early 1970s, the unrestricted Hubbard (UHB) model involving the on-site repulsion integral (*U*) [112] was used as a practical theoretical model for quasi-degenerated systems as an extended MO model of the EHMO [126]. The instability condition for the closed shell pair by UHB was simply given by Equation (14). The ab initio BS UHF model was also used as a first step toward quasi-degenerated systems such as organic peroxides [26,82]. The UHF coupled cluster (UCC) method was proposed for the inclusion of dynamical correlation effects, as shown in Figure 15 [32]. The BS Hartree–Fock–Slater (UHFS) model was also used for the metal-metal bonds with the magnetic property [25]. The CC model starting from the natural orbitals (UHFS NO = UNO) of UHFS solutions was proposed for transition metal clusters [25], although Brillouin’s theorem was not satisfied for UHFS. However, the theorem was assumed to be recovered by the UCCS cycle [32]. The UHFS model was later extended to the hybrid UDFT (UHDFT) model, such as the UB3LYP model [117,118,119], providing the UNO (UB3LYP) UCCSD(T) approach. However, these methods are based on the single determinant model [25], indicating the difficulty of theoretical investigations of the excited states.

**Figure 24:**
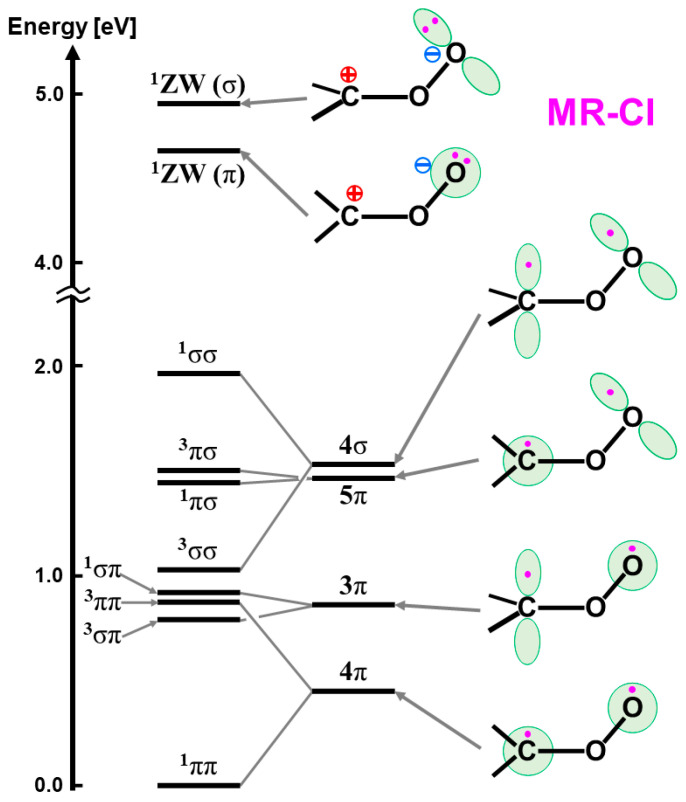
The energy levels of the eight diradical states consisted of the localized π and σ-type orbitals; ^1,3^(π^1^π^1^), ^1,3^(σ^1^π^1^), ^1,3^(π^1^σ^1^), and ^1,3^(σ^1^σ^1^), and two zwitterionic (ZW); ^1^(π^0^π^2^) and ^1^(σ^0^σ^2^), states of CH_2_OO by UHF NO (UNO) CI [98]. The isolobal and isospin analogy between R_1_R_2_C-O-O and L_1_L_2_Fe-O-O predicted that singlet ^1^(π^1^π^1^) ground state of the iron-peroxides [26,29].

In the 1980s, beyond BS models based on the multi-Slater-determinants [31,32] were proposed as a next step toward the quasi-degenerate systems. To this end, the natural orbitals (UNO) obtained by the natural orbital (NO) analysis of the BS UHF (UHFS) solutions were used for the construction of a complete active space (CAS) for configuration interaction (CI) and coupled cluster (CC) calculations, as illustrated in Figure 15 [25,32]. For example, the energy levels of the ground and lower-lying singlet and triplet diradical states and zwitterionic (ZW) states of CH_2_OO obtained by the UHF NO (UNO) CI method are illustrated in Figure 24 [98]. The multi-reference (MR) UNO CI, CC, and many-body perturbation (MBPT) by the use of UNO of UHFS were proposed for the transition metal complexes with open-shell character [25,32,100]. 

Bofill and Pulay [241] later revisited the UNO CI [32] for transition metal systems, demonstrating its practical utility. The UNO density matrix renormalization group (DMRG) CAS CI was successfully applied to elucidate the energy levels of the MnO_2_Mn complex [242]. The UNO was also used for trial orbitals for domain-based localized pair-natural orbital (DLPNO) CCSD(T0) computations of the CaMn_4_O_x_ clusters in PSII [80,104]. Thus, UNO (ULO) obtained by UHDFT is now practically useful for CAS CI, CASCF, and MR CI (CC) computations of large quasi-degenerated systems [25,100], for which CASSCF is hardly possible [32,99] (see Section 6.4). Chemical indices are also obtained by these computations, as shown in Figure 14B (and see later).

### 6.2. CASPT2 and RASPT2 Results for Iron-Oxo Compound

In this section, the computational results for heme-type iron-oxo compounds by hybrid DFT (HDFT) are compared with those of CAS second-order perturbation (PT2) and restricted active space (RAS) PT2 [243,244,245]. The HDFT computations were very useful for full geometry optimizations of the possible reaction intermediates for mono-oxygenations by P450, as shown in Figure 21. Indeed, the optimized Fe-O and Fe-S bond lengths were calculated to be 1.61~1.65 (1.74~1.78) and 2.51~2.63 (2.26~2.35) Å for ^2^[Por(+•)(SCH_3_)Fe(IV)=O], where the corresponding values for ^2^[Por(SCH_3_)Fe(V)=O] (^2^E) were given in parentheses [143,144,145,146]. The optimized values for the ^2^Cpd I_a_ were compatible with the experiments for Cpd I [21]. The Fe-O distance for ^2^E was longer than that of ^2^Cpd I_a_ because of the oxyl-radical character [145]. Thus, full geometry optimizations by HDFT are practical procedures before the beyond DFT computations.

However, the relative energies among the intermediates were variable with the weight of the UHF component involved in the HDFT solutions such as UB3LYP [103,243,244,245]. For example, the energy gaps between ^2^[Por(+•)(SCH_3_)Fe(IV)=O] and ^2^[Por(SCH_3_)Fe(V)=O] were 10.3, 11.3, 7.3, 6.4, and 11.5 kcal/mol, respectively, by unrestricted B3LYP, CAM-B3LYP, HSE06, M06, and ωB97X [103]. Therefore, beyond BS HDFT computations were inevitable for elucidation of scope and reliability of the relative stabilities among them at the DFT level. 

Judging from the computational results reviewed, we noticed the following three important conditions for quantitative calculations of the relative energies among key intermediates for oxygenation reactions by transition metal oxo and peroxo compounds [25,31,32,99,100,101,102]:

1. Use of large active space for construction of the reference states for MR methods;
2. Use of flexible basis sets for inclusion of dynamical correlation effects;
3. Dynamical correlation corrections by higher-order methods than the PT2 level.

However, it is very difficult to meet these demands. Therefore, as a first step, we employed the BS computations for transition metal oxides with strongly correlated electron systems (SCES). As a second step, the natural orbital analysis of the BS solutions has been performed to elucidate the active natural orbitals for SCES, as shown in Figure 15. At the moment, as a third step, accurate computational methods may be classified into three different classes, (1) MR method, (2) single reference (SR) method, and (3) exact diagonalization method. 

Radoń et al. [103] indeed performed the beyond HDFT calculations of two heme compounds; [PorFe=O]^+^ and PorClFe=O]^0^ (P = porphyrin) using extensive basis sets. The beyond HDFT methods employed by them are the MR ab initio methods, namely, CAS and RAS self-consistent field (SCF) methods followed by the second-order perturbation calculations (PT2) for electron correlation corrections. CASSCF/CASPT2 and RASSCF/RASPT2 calculations [103] were performed with Molcas program [246] using a scalar-relativistic second-order Douglas–Kroll (DK2) Hamiltonian, and the IPEA-shifted zero-order Hamiltonian for the PT2 calculations. Single point calculations were performed, assuming the full optimized structures of the iron-oxo compounds by using the UBP86/def2 TZVP method. Two types of atomic natural orbitals (ANO) basis sets (basis I and II) were used for their single point state specific CASPT2 and RASPT2 calculations. The small one (basis set I) was given by the contracted [7s,6p,5d,2f,1g] for iron, [4s,3p,1d] for C, N, and O, [5s,4p,2d] for Cl, and [2s] for H. The larger one (basis set II) was composed of the contracted [7s,6p,5d,3f,2g,1h] for iron, [4s,3p,2d,1f] for C, N, and O, [5s,4p,3d,2f] for Cl, and [3s,1p] for H.

The CAS space crucial for the CASSCF calculations of the PorClFe=O in **4a** in Figure 17 was found to be the 15 electrons and 16 orbitals [15e, 16o]. On the other hand, the large RAS space for the RASSCF calculations was taken to be the [29e, 28o], although the RAS [37e, 36o] was necessary for the inclusion of all π bonding and anti-bonding orbitals, namely, full valence MCSCF in Figure 15. Table 12 summarized the energy differences between Por(+•)ClFe(IV)=O (^4(2)^A_2_) and PorClFe(V)=O (^2^E) states by UB3LYP/basis set II, CASPT2 [15e, 16o]/basis set II and RASPT2 [29e, 28o]/basis set II, where the corresponding values for Por(+•)Fe(IV)=O (^4(2)^A_2_) and PorFe(V)=O (^2^E) states without the axial Cl anion are given in parentheses. 

From Table 12, the calculated energy gaps between the quartet (^4^A_2_) and doublet (^2^A_2_) states of Por(+•)ClFe(IV)=O were 0.1 (0.4), −1.4 (−0.6), and 0.0 (0.4) (kcal/mol), respectively, by UB3LYP, CASPT2, and RASPT2, indicating no serious energy differences. On the other hand, the energy gap between ^4^A_2_ and ^2^E was calculated to be 12.4 kcal/mol for PorClFe=O (PorFe=O) by UB3LYP, which is consistent with the calculated values by many other groups [147,148,149,150,151,152,153,154,155,156,157,158,159,160,161,162,163,164,165,166,167,168,169,170,171,172,173]. The calculated energy gap was 12.7 kcal/mol for PorFe=O by UB3LYP, indicating no serious difference with and without the fifth ligand (Cl^−^) at the UB3LYP level of theory. 

Interestingly, the energy gap between ^4^A_2_ and ^2^E was calculated to be 1.6 kcal/mol for PorClFe=O by CASPT2 [15e, 16o], indicating the small energy difference in a sharp contrast to the UB3LYP results. Moreover, the ^2^E state was more stable by 3.4 kcal/mol than ^4^A_2_ in the case of PorFe(V)=O without the axial fifth ligand. The energy gap between ^4^A_2_ and ^2^E of these species was calculated to be −1.7 (−6.5) kcal/mol for PorClFe(V)=O by RASPT2 [29e, 28o], indicating the greater stability of ^2^E than ^4^A_2_ in gas phase. The effect of the fifth ligand (Δ) for the energy gap was about 5 kcal/mol by these beyond HDFT methods by the CAS(RAS) PT2 level of theory, including dynamical electron correlations [103]. 

**Figure 25:**
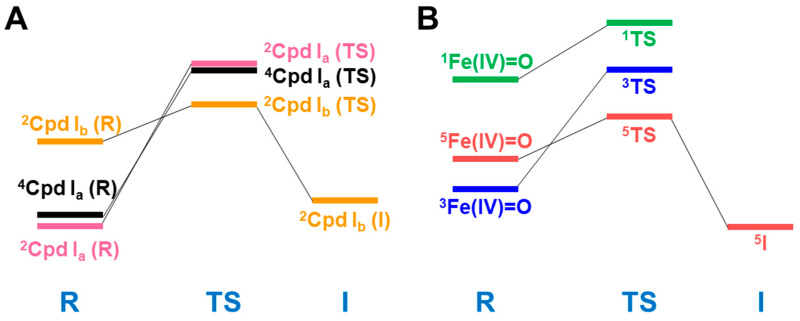
(**A**) Three state models of the P450 and related heme-type iron-oxo compounds [103,146] and (**B**) Three state models for non-heme iron-oxo compounds [186,243,244,245] concluded by the HDFT and related beyond BS computations.

**Figure 26:**
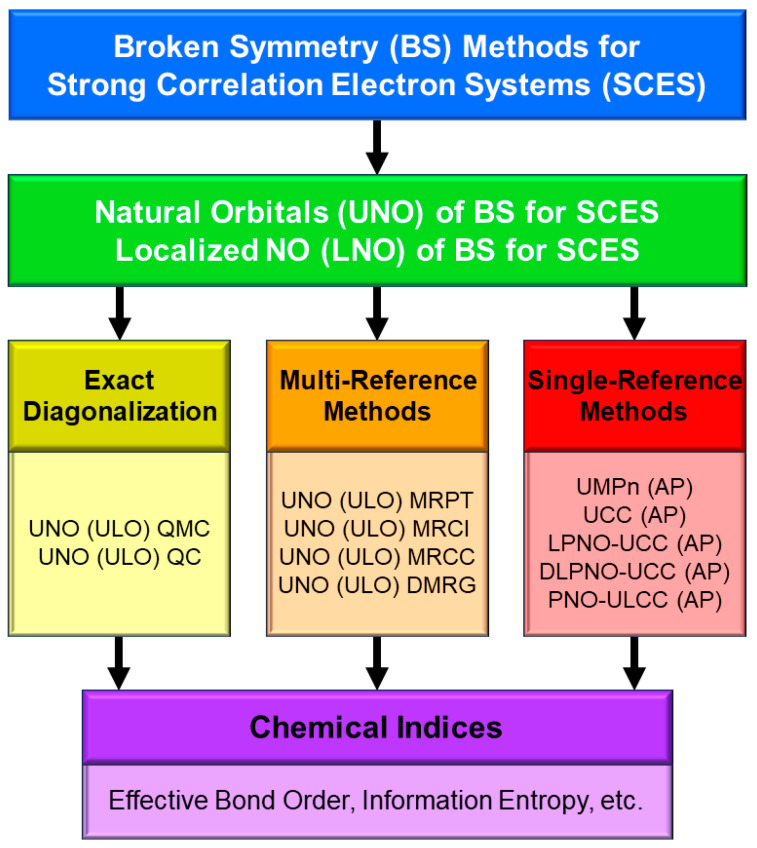
Computational schemes for strongly correlated electron systems (SCES) such as M=O and MOO systems starting from full geometry optimizations of them by BS HDFT solutions followed by the natural orbital analysis to obtain UNO (ULO) CAS space for multi-reference (MR) PT, CI, and CC computations. UNO(ULO) are useful for QMS and QC computations for SCES. The SR UCC methods are also applicable for systems without significant MR character since CCSD(T) and CCSDT computations are reliable.

Table 13 summarizes the energy gap between ^4^A_2_ and ^2^E states of PorClFe(V)=O, including the solvation energy correction obtained by UB3LYP COSMO calculations [103]. The average solvation energy correction ΔΔ is found to be 5.6 kcal/mol. The energy gap (−1.7 kcal/mol) by RASPT2 in the gas phase in Table 13 may be changed to 3.9 kcal/mol by adding the ΔΔ value, as shown in Table 13. Judging from the computational results beyond HDFT methods, the energy gap between ^4^A_2_ and ^2^E states are a few (2~4) kcal/mol for PorClFe=O in the solution phase, indicating that the ^2^E state is thermally accessible for the mono-oxygenation reactions by P450, as shown in Table 13 [83,84,85,86,87,88,89,90]. This means the curve crossings occur along the HRA pathway, as illustrated in Figure 25A. However, the accurate MR CC and future quantum computation (QC) approach are inevitable to elucidate the scope and reliability of the RASPT2 result, as shown in Figure 15; Figure 26 [32]. Nevertheless, beyond HDFT computations [103] have elucidated a theoretical possibility of the ET-PT process for alkane hydroxylation by the native P450 [146]; ^2^E (Fe(V)=O) + H-CR → ^2^[Fe(IV)-O^−^ + H^+^…CR] (PT-ET) → Fe(III) + HOR, instead of the HRA, followed by the radical rebound process [83,84,85,86,87,88,89,90].

### 6.3. Beyond HDFT Computations for Non-Heme Iron-Oxo Compounds

In this section, the computational results for the non-heme iron-oxo species by hybrid DFT (HDFT) are compared with beyond DFT results [243,244,245]. The single reference (SR) method is applicable for transition metal oxides under the BS approximation. The UCCX (X = SD, SD(T), SDT, etc.) is regarded as an SR method in our classification [25,32]. However, UCCX methods are hardly applicable for transition metal oxo and peroxo complexes with large ligands such as porphyrin [78,79]. Local methods such as DLPNO UCCSD(T) are used as practical methods for them [104]. Recently, Harvey and his collaborators [243,244,245] have performed extensive beyond HDFT computations for non-heme iron-oxo Fe(IV)=O enzymes to elucidate activation barriers for hydrogen radical abstraction (HRA) reactions. They have considered a model complex, (NH_3_)_5_Fe(IV)=O (**XII**) with the Oh ligand field in Figure 8A, and a synthetic model complex, (N4PyFe(IV)=O) (N4Py = *N*,*N*-bis(2-pyridylmethyl)-*N*-bis(2-pyridyl)methylamine) (**XIII**). Full geometry optimizations have been performed by the UB3LYP/DZ basis set. The one-shot computations at the optimized geometries were performed with several beyond HDFT methods using very large basis sets. Table 14 summarizes the computational results [243,244,245]. The PCM methods are used for the evaluation of the solvation effects for reaction complex (RC), TS, and radical intermediate (I) for HRA.

The energy gaps (ΔE(T-Q)) between the triplet (*S* = 1) and quintet (*S* = 2) states are highly dependent on the systems and the computational methods. The ΔE(T-Q) values for **XII** are in the range of 0.5~3.1 kcal/mol by UB3LYP, UB3LYP-D3, UNO(UB3LYP)-UCCSD(T), indicating the greater stability of the triplet state than the quintet state. On the other hands, the corresponding values are in the range of −6.0~−0.5 kcal/mol by UNO(UHF) UCCSD(T), DLPNO-UCCSD(T), CASSCF, and CASPT2, indicating the reverse tendency as shown in Table 14. This tendency by CASPT2 is reversed by adding the correlation correction (CC) of the (3s3p) part of Fe, providing 0.5 kcal/mol for the gap by CASPT2/CC involving the 3sp correlation correction (CC). Interestingly, the ΔE(T-Q) values for **XII** are 7.6 and 10.0 kcal/mol by UB3LYP and UB3LYP-D3, respectively. They are 11.4 and 14.0 kcal/mol by CASPT2 and CASPT2/CC, indicating the large energy gap for **XII**, indicating large ligand field effects.

The activation barriers from the reacting complex (RC) to the transition structure (TS) for HRA on the triplet surface for **XIII** are 21.7 (10.4), 21.3 (10.9), and 27.4 (16.7) kcal/mol by UB3LYP, UB3LYP-D3, and CASSCF (12e, 12o), respectively, the corresponding values for the quintet state are given in parentheses as shown in Table 14. On the other hand, they are 29.6 (17.2), 27.6 (17.0), 30.4 (16.7), 31.7 (15.7), and 29.2 (13.7) kcal/mol by UNO(UB3LYP) UCCSD(T), UNO(UHF) UCCSD(T), DLPNO UCCSD(T), CASPT2, and CASPT2/CC. The activation barriers for HRA by all the computational methods employed are smaller for the quintet state than those of the triplet state. UB3LYP and UB3LYP-D3 underestimate the activation barriers for **XIII** by 7.5 (3.3) and 7.9 (2.8) kcal/mol when compared to that of CASPT2/CC, respectively. Judging from the activation barriers, HRA is not possible for the triplet surface of **XIII**, whereas those of the quintet state are 14~17 kcal/mol, which are compatible with the computational results for P450. Thus, the quintet state of the non-heme Fe(IV)=O have exhibited strong radical reactivity like the triplet atomic oxygen, as shown in Figure 25B.

The activation barriers from RC to TS for HRA on the triplet (quintet) surface for **XIII** are 14.3 (3.5) and 13.3 (3.5) kcal/mol by UB3LYP and UB3LYP-D3, respectively. On the other hand, they are 25.4 (8.8), 24.3 (8.4), and 23.8 (9.0) kcal/mol by DLPNO UCCSD(T), CASPT2 and CASPT2/CC, respectively. The activation barriers for HRA by all the computational methods employed are smaller for the quintet state than those of the triplet state. UB3LYP and UB3LYP-D3 underestimate the activation barriers for **XIII** by 9.5 (5.5) and 10.5 (5.5) kcal/mol when compared with that of CASPT2/CC, respectively. Judging from the activation barriers, HRA is hardly possible for the triplet surface of **XIII**, whereas those of the quintet state are 8.4~9.0 kcal/mol, which are compatible with those involving solvation energies by other groups [104].

The curve crossing between the triplet and quintet states has been proposed for HRA by the non-heme Fe(IV)=O systems with the O*_h_* ligand field since the triplet state is often more stable than the quintet state at the reactant complex (RC) stage, and this tendency is reversed at the transition structure (TS). However, CASPT2 has predicted the ground triplet surface along HRA, indicating no surface crossing because of the large positive T-Q gap (14 kcal/mol). On the other hand, the ground quintet surface with no crossing has been predicted for **XIII** by DLPNO UCCSD(T) computations starting from UNO of UB3LYP because DLPNO-CCSD(T) over-stabilizes the quintet state, namely, the large negative T-Q gap (−6 kcal/mol). Interestingly, UNO(UB3LYP) UCCSD(T) has provided the S-Q crossing like CASPT2/CC, as illustrated in Figure 25B. Thus, UCCSD(T) without the local approximation works well even for the complex non-heme iron systems. The RASPT2 based on large RAS space is desirable for comparisons with CASPT2/CC [243,244,245]. 

### 6.4. Future Perspective and Outlook for Quantum Computations

In this final section, exact quantum computations (QC) of M=O and MOO systems have been considered for future perspective and outlook. Full valence MCSCF without ambiguity in Figure 15 is impossible even now for M=O and MOO systems with large ligands. Therefore, DMRG methods [247] have been used for the construction of important CAS spaces for these systems [242,243,244,245]. The second-order perturbation (PT2) methods, such as CASPT2 [101], NEVPT2 [248], etc., are employed for the inclusion of the dynamical correlation corrections, as shown in Table 10 and Table 11. Selection of the basis sets is also crucial for reliable MR PT2 computations [101,102]. MR CCSD(T) computations [249] in Figure 15 are desirable as an extension of the MR PT2 methods. However, Mk-MRCCSD [249] computation is limited to transition metal complexes with small active space. MRCC approach [32] remains as a future problem for P450 and related systems with large MR reference [103].

The exact diagonalization of ab initio Hamiltonians is one of the useful methods, as shown in Figure 26. Quantum Monte Carlo (QMC) computations [250] based on the ab initio Hamiltonians have been proposed for SCES. Recently, quantum computations (QC) have been proposed for the exact diagonalization of large Hamiltonian matrix whose sizes increase exponentially with the number of electrons of molecules, as shown in Figure 26 [33,34,35,36]. The FeMoco and 8Fe-7S clusters for nitrogen fixation are considered as target molecules for the future QC because of the large active space [33,34,35,36]. However, quantum algorisms are still developing for the well-balanced inclusion of both non-dynamical and dynamical correlations [251]. Thus, the computational methods are developing along the line; HF → HDFT → UCCSD(T) → MR PT (CASPT2) → MR CI(CC) → QMC → QC, as illustrated in Figure 26.

Very recently, Goings et al. [252] have evaluated the runtimes and logical qubit requirements for QC of P450 systems on the quantum phase estimation algorism [33] in comparison with the computational methods, such as DMRG [247] with 40 active orbitals on the classical computer, in Figure 26. Their DMRG computations on the classical computer [252] indeed provided the negative *J*_ab_ value (−21.3 cm^−1^) for the doublet–quartet gap in accordance with the HDFT (−20.4 cm^−1^) and experimental (−18 cm^−1^) results in Table 6, confirming three important orbitals, as shown in Figure 16A. Interestingly, they estimated that 9000 logical qubits are necessary for QC using 500 active orbitals of P450. We hope for rapid developments of the QC systems for the accurate computations of metalloenzymes [252]. At the moment, interplay between classical computation and experiments is considered as a practical and feasible approach to transition metal oxo and peroxo enzymes embedded in a protein matrix [25,26,27,28,29,99,100,101,102,243,244,245]. 

In this review, we could not touch the QM/MM/MD computations [105,106,107,108] of mono-oxygenations by the native P450 enzymes to elucidate crucial roles of the native protein matrix [253,254,255]. Recently, machine learning (ML) methods have been introduced for directed evolutions of proteins, providing PorLFe=CHR core in the modified P450 enzymes [256,257]. The isolobal and isospin analogy among O, NH, and CH_2_ is useful for understanding the structure and bonding of Fe=X (X = O, CH_2_, NH) with the optimized mutational protein matrix [258]. The quantum machine learning (QML) method will be also necessary for the inclusion of the QM computational results discussed in this review [259,260]. Developments of the QC methods are expected for several theoretical approaches to 3d transition metalloenzymes and the design of artificial model systems.

## Figures and Tables

**Table 1:** Spin states and orbital configurations of transition metal oxides [M(X)OO]^X−2^ and charge populations by the broken-symmetry (UHF) methods [29,82].

[M(X)OO] ^(a)^	2S + 1	δ_1_	δ_2_	dσ	dπ_x_	dπ_y_	dπ_x_*	dπ_y_*	Fe ^(c)^	O_1_^(b)^	O_2_^(b)^
Cr(II)OO ^(c)^	3	↑	0	↑	↑	↑	↓	↓	1.86	−0.37	0.51
	7	↑	0	↑	↑	↑	↑	↑	1.86	−0.43	0.57
L_1_Cr(II)OO ^(c,d)^	3	↑	↑	↑↓	↑	↑	↓	↓	1.40	−0.51	−0.26
	7	↑	↑	↑↓	↑	↑	↑	↑	1.43	−0.69	−0.38
Fe(II)OO ^(c)^	3	↑	↑↓	↑	↑	↑	↓	↓	1.82	−0.41	0.59
	7	↑	↑↓	↑	↑	↑	↑	↑	1.84	−0.44	0.60
L_1_Fe(II)OO ^(d)^	3	↑	↑	↑↓	↑	↑	↓	↓	1.24	−0.35	0.08
L_12_Fe(II)OO ^(d)^	3	↑	↑	↑↓	↑	↑	↓	↓	1.42	−0.31	−0.01
L_123_Fe(II)OO ^(d)^	3	↑	↑	↑↓	↑	↑	↓	↑	1.44	−0.31	−0.07
L_13_Fe(II)OO ^(d)^	3	↑	↑	↑↓	↑	↑	↓	↑↓	1.40	−0.62	−0.44
L_133_Fe(II)OO ^(d)^	3	↑	↑	↑↓	↑	↑	↓	↓↓	1.41	−0.56	−0.51

^(a)^ [M(X)O]; Formal oxidation numbers are X and −2 for M and OO, respectively. ^(b)^ Net charges on the oxygen site and the corresponding value (X − 2 − m) on the metal site. ^(c)^ The energy gaps between *S* = 1 and *S* = 3 are 6.5, 8.5, and 5.1 (kcal/mol) for Cr(II)OO, L_1_Cr(II)OO and Fe(II)OO, respectively. ^(d)^ L_1_ = (NH_2_)_4_, L_12_ = (NH_2_)_4_(NH_3_), L_13_ = (NH_2_)_4_(↓•SH), L_123_ = (NH_2_)_4_(NH_3_)(H_2_O), L_133_ = (NH_2_)_4_(↓•SH)(H_2_O). The up and down arrows are denoted the up and down spins of electron, respectively.

**Table 2:** Spin states and orbital configurations of transition metal oxides [M(X)O]^X−2^ and charge populations by the broken-symmetry (UHF) methods [27,81].

[M(X)O] ^(a)^	2*S* + 1	δ_1_	δ_2_	σ	π_x_	π_y_	π_x_*	π_y_*	ΔE ^(b)^	N.X. ^(c)^
Cr(III)O	4	↑	↑	↑↓	↑↓	↑↓	0	↑	0.0	−0.19
	6	↑	↑	↑↓	↑↓	↑	↑	↑	30.6	−0.42
Cr(IV)O	3	↑	↑	↑↓	↑↓	↑↓	0	0	0.0	0.35
	7	↑	↑	↑↓	↑	↑	↑	↑	39.0	0.30
Cr(V)O	2	↑	0	↑↓	↑↓	↑↓	0	0	0.0	0.96
	6	↑	0	↑↓	↑	↑	↑	↑	70.0	0.65
Mn(III)O	3	↑	0	↑↓	↑↓	↑↓	0	↑	0.0	−0.04
	5	↑	0	↑↓	↑	↑	↑	↑	44.0	−0.04
Mn(IV)O	2	↑	0	↑↓	↑↓	↑↓	0	0	0.0	0.33
	4	↑	0	↑↓	↑↓	↑	0	↑	36.0	0.29
Mn(V)O	3	↑	↑	↑↓	↑↓	↑↓	0	0	0.0	1.08
	7	↑	↑	↑↓	↑	↑	↑	↑	64.4	1.06
Fe(II)O	5	↑	↑	↑↓	↑↓	↑	0	↑	0.0	−0.72
	7	↑	↑	↑↓	↑	↑	↑	↑	12.9	−0.62
Fe(III)O	4	↑	↑	↑↓	↑↓	↑↓	0	↑	0.0	−0.55
	6	↑	↑	↑↓	↑↓	↑	↑	↑	18.4	−0.59
Fe(IV)O	3	↑↓	0	↑↓	↑↓	↑↓	↑	↑	0.0	0.55
	1	↑↓	0	↑↓	↑↓	↑↓	↑	↓	26.7	0.35

^(a)^ [M(X)O]; Formal oxidation numbers are X and –2 for M and O, respectively. ^(b)^ kcal/mol, ^(c)^ N. X; net charge (m) of the oxygen site and the corresponding value (X − 2 − m) on the metal site. The up and down arrows are denoted the up and down spins of electron, respectively.

**Table 3:** The optimized bond length (Å) of transition metal oxides by the BS methods [78,79].

System	Spin State	UHF		UB3LYP		UBLYP		UCCSD(T)	Exp.
		(1) ^(a)^	(2) ^(b)^	(1) ^(a)^	(2) ^(b)^	(1) ^(a)^	(2) ^(b)^	(2) ^(b)^	
CrO	^5^Π	1.90	1.86	1.66	1.62	1.65	1.62	1.66	1.615
MnO	^6^Σ^+^	1.88	1.87	1.66	1.63	1.67	1.64	1.69	1.648
FeO	^5^Δ	1.84	1.83	1.63	1.61	1.64	1.62	1.65	1.619
CoO	^5^Δ	1.85	1.84	1.64	1.63	1.66	1.65	1.64	1.631
	^4^Σ^−^	1.79	1.79	1.61	1.59	1.62	1.61	1.62	
NiO	^3^Σ^−^	1.80	1.81	1.63	1.63	1.65	1.65	1.64	1.631
CuO	^2^Π	1.92	1.87	1.82	1.77	1.80	1.75	1.80	1.724

^(a)^ BS I (1); Huzinaga MIDI [533(21)/5(21)/(41)] for transition metals and 6-31G for O. ^(b)^ BS II (2); MIDI plus pdf-polarization MIDI [533(21)/5(21)1*/(41)1*/1*] for transition metals and 6-31G* for O.

**Table 4:** The binding energies (eV) of transition metal oxides by the BS methods [78,79].

System	Spin State	UHF		UB3LYP		UBLYP		UCCSD(T)	Exp.
		(1) ^(a)^	(2) ^(b)^	(1) ^(a)^	(2) ^(b)^	(1) ^(a)^	(2) ^(b)^	(2) ^(b)^	
CrO	^5^Π	0.44	1.24	3.42	4.34	4.48	5.35	3.71	4.57
	^7^Π	0.12	0.98	2.26	2.92	2.72	3.29		
MnO	^6^Σ^+^	−0.28	1.13	2.50	4.09	3.64	5.26	2.94	3.83
	^4^Π	−0.39	0.80	2.08	2.94	3.29	4.27		
	^4^Σ	−1.34	0.00	1.57	2.98	2.80	4.23		
	^8^Π	−0.03	1.43	1.32	2.58	1.70	2.94		
FeO	^5^Δ	−0.39	1.05	2.89	4.38	4.07	5.34	3.21	4.17
	^3^Φ	−0.19	1.03	2.47	3.36	3.72	4.38		
	^5^Σ^+^	−0.90	0.48	2.96	3.91	4.21	4.82	2.96	
	^7^Σ^+^	−1.31	0.31	1.99	3.40	2.96	4.08		
CoO	^4^Δ	−1.05	0.35	3.00	4.07	4.36	5.43	2.92	3.94
	^4^Σ^−^	−2.10	0.67	2.06	3.74	3.39	5.20	2.61	
	^2^Δ^−^	−0.43	0.78	2.35	3.44	3.70	4.85		
	^6^Δ	1.46	0.18	1.83	3.21	2.94	4.36		
NiO	^3^Σ^−^	−1.73	−0.19	3.26	4.13	4.19	5.59	3.13	3.91
	^1^Σ^−^	−2.76	−1.37	2.82	3.72	3.81	5.24		
	^5^Σ	−0.54	0.37	1.72	2.69	2.46	4.01		
	^5^Δ	0.00	0.01	1.62	2.28	2.17	3.30		
	^5^Φ	−0.47	0.91	1.47	2.30	1.41	2.94		
CuO	^2^Π	0.08	0.61	2.27	2.69	2.80	3.18	2.37	2.75
	^4^Σ^−^	−0.26	0.02	0.74	0.93	1.50	1.57		

^(a)^ BS I(1);Huzinaga MIDI [533(21)/5(21)/(41)] for transition metals and 6-31G for O. ^(b)^ BS II (2) MIDI plus pdf-polarization MIDI [533(21)/5(21)1*/(41)1*/1*] for transition metals and 6-31G* for O.

**Table 5:** The bond orders for high-valent transition-iron oxo bonds with the octahedral ligand (O*_h_*) and trigonal bipyramidal (TBP) ligand fields [27,82].

No.	Systems	δ_xy_	σ	π_zx_	π_zy_	π_zx_*	π_zy_*	δ_x2−y2_	σ*	BO
1	^2^Fe(V)=O	2	2	2	2	1	0	0	0	2.5
2	^2^Fe(V)=O	2	2	2	2	0	0	0	1	2.5
3	^4^Fe(V)=O	1	2	2	2	1	0	1	0	2.5
4	^4^Fe(V)=O	1	2	2	2	0	0	1	1	2.5
5	^4^Fe(V)=O	1	2	2	2	1	1	0	0	2.0
6	^4^Fe(V)=O	1	2	2	2	1	0	0	1	2.0
7	^2^••Fe(V)–O••	2	2	1 + *T*	1 + *T*	1 − *T*	1 − *T*	0	1	0.5 + 2*T*
8	^2^•Fe(V)–O•	2	2	2	1 + *T*	1	1 − *T*	0	0	1.5 + *T*
9	^2^•Fe(V)–O•	2	2	2	1 + *T*	1	0	0	1 − *T*	1.5 + *T*
10	^2^•Fe(V)–O•	2	2	1 + *T*	2	1	0	0	1 − *T*	1.5 + *T*
11	^2^•Fe(V)–O•	2	1 + *T*	2	2	1	0	0	1 − *T*	1.5 + *T*
12	^4^•Fe(V)–O•	1	2	2	1 + *T*	1	1 − *T*	1	0	1.5 + *T*
13	^3^Fe(IV)=O	2	2	2	2	1	1	0	0	2.0
14	^3^Fe(IV)=O	2	2	2	2	1	0	0	1	2.0
15	^3^Fe(IV)=O	2	2	2	2	0	1	0	1	2.0
16	^1^Fe(IV)=O	2	2	2	2	1	1	0	0	2.0
17	^3^•Fe(IV)=O•	1	2	1 + *T*	2	1 − *T*	2	1	0	2.0 − *T*
18	^3^•Fe(IV)=O•	1	2	2	1 + *T*	2	1 − *T*	1	0	2.0 − *T*
19	^3^•Fe(III)–O•	2	2	2	1 + *T*	1	1 − T	0	1	1.0 + *T*
20	^3^•Fe(III)–O•	2	2	2	1 + *T*	1	1	0	1 − *T*	1.0 + *T*
21	^3^•Fe(III)–O•	2	1 + *T*	2	2	1	1	0	1 − *T*	1.0 + *T*
22	^5^Fe(IV)=O	1	2	2	2	1	1	1	0	2.0
23	^5^Fe(IV)=O	1	2	2	2	1	1	0	1	1.5
24	^5^•Fe(III)–O•	1	2	2	1 + *T*	1	1 − *T*	1	1	1.0 + *T*
25	^5^•Fe(III)–O•	1	2	2	1 + *T*	1	1	1	1 − *T*	1.0 + *T*
26	^5^•Fe(III)–O•	1	1 + *T*	2	2	1	1	1	1 − *T*	1.0 + *T*

*T* is the orbital overlap between bifurcated orbitals in Equation (20). The *S* = 1/2 (*S* = 3/2) and *S* = 1 (*S* = 2) are the ground states for Fe(V)=O and Fe(IV)=O for the O*_h_* (TBP) ligand fields, respectively, and many other excited states are also feasible. The effective bond order (BO) is reduced by the orbital bifurcation (*T* < 1.0) via the spin polarization (SP) effect.

**Table 6:** Calculated effective exchange integral (*J* cm^−1^) values for the compound I complexes [143].

Model	Catalase	Peroxidase ^(a)^		P450	Model Complex	
	1	2a	2b	3	4a	4b
*J*_calc._	−29.3	9.1	59.9	−20.4	40.66	29.57
*J*_exp._	6 ^(b)^	−1.3 < *J*_exp_^(c)^ < 1.3		−18 ^(d)^	-	21.5

^(a)^ **2a**: deprotonated, **2b**: protonated. ^(b)^ Estimated by *J* = −0.2*D* and *D*~30 cm^−1^. ^(c)^ HRP. ^(d)^ CPO, estimated by *J* = −0.51*D* and *D*~35 cm^−1^.

**Table 7:** The *J* values for binuclear transition metal complexes by the broken-symmetry methods [27,28].

System	Conf.	*J*_ab_ (R(M–O), Angle)			
Cr^III^OCr^III^	d^3^-d^3^	−6204 (1.0)	−1987 (1.25)	−671 (1.5)	−377 (1.7)
XCr^III^OCr^III^X	d^3^-d^3^	−187 (1.7)	−79 (1.8)	−10 (1.9)	
Mn^0^…Mn^0^	d^5^-d^5^	−32 (3.0)	−7 (3.5)		
Mn^II^OMn^II^	d^5^-d^5^	−3534 (1.0)	−156 (1.5)	7 (2.0)	
XMn^II^OMn^II^X	d^5^-d^5^	−24 (1.71)			
XMn^III^OMn^III^X	d^4^-d^4^	−60 (1.71)			
Fe^III^OFe^III^	d^5^-d^5^	−4913 (1.0)	−264 (1.5)	−71 (1.6)	279 (1.8)
		326 (1.9)			
Ni^II^ONi^II^	d^8^-d^8^	−14754 (1.0)	−831 (1.5)	−525 (1.7)	
XNi^II^ONi^II^X	d^8^-d^8^	−174 (1.7)			
Cu^II^OCu^II^	d^9^-d^9^	−36453 (1.0)	−4621 (1.6)	−5433 (1.8)	
Cu^III^OCu^III^	d^8^-d^8^	−19616 (1.0)	−5671 (1.6)	−5688 (1.8)	
Cu^II^(OH)_2_Cu^II^	d^9^-d^9^	554 (2.85, 97)	207 (2.98, 103)		
		−175 (3.15, 110)	−170 (3.12, 110)		
H_2_Cu^II^(OH)_2_Ni^II^H_2_	d^9^-d^8^	−24 (2.85, 95.6)	−170 (3.15, 110)		
Fe^III^…Fe^III^	d^5^-d^5^	−16 (2.70, 75)			
Fe^III^S_2_Fe^III^	d^5^-d^5^	−926 (2.70, 75)			
H_2_Fe^III^S_2_Fe^III^H_2_	d^5^-d^5^	−175 (2.70, 75)			

**Table 8:** Charge and spin densities of the iron complexes generated in the P450 reaction cycle by the UB3LYP method [143].

	Spin State		Fe	S	N	O	O(2)
**A**	*S* = 1/2	Charge	0.57	0.22	−0.07	0.10 (H_2_O)	
		Spin	1.02	0.06	0.00	0.00 (H_2_O)	
**C**	*S* = 2	Charge	1.06	−0.15	0.12	-	
		Spin	3.86	0.23	0.05	-	
	*S* = 1	Charge	0.87	−0.15	−0.11	-	
		Spin	2.01	0.26	−0.01	-	
	*S* = 0	Charge	0.70	0.09	−0.11	-	
		Spin	1.09	−0.14	−0.05	-	
**D**	*S* = 0	Charge	0.17	−0.02	0.23	−0.18 (O1)	−0.19 (O2)
		Spin	1.08	0.06	0.02	−0.66 (O1)	−0.39 (O2)
**E**	*S* = 1/2	Charge	0.24	−0.13	0.21	−0.20 (O1)	−0.25 (O2)
		Spin	0.93	−0.02	−0.05	0.38 (O1)	0.59 (O2)
**F**	*S* = 1/2	Charge	0.20	0.10	0.19	−0.13	
		Spin	1.19	−0.81	0.04	0.91	

**Table 9:** Chemical indices for iron-peroxides (**D** and **E**) and iron-oxo (**F**) intermediates by the UB3LYP calculation [143].

Entry	Orbital	*Q*	*U*	*I*	*b*	*Y*	*B*
**D**	σ	0.220	0.048	0.970	0.975	0.000	0.999
	π	0.966	0.934	0.207	0.257	0.517	0.482
**E**	σ (SP)	0.192	0.037	0.977	0.981	0.000	0.999
	π	0.898	0.806	0.380	0.441	0.261	0.738
**F**	π	0.996	0.992	0.067	0.088	0.824	0.175

**Table 10:** Spin density populations for the transition structures of hydrogen radical abstraction reactions from alkanes (**II**–**XI**) by the ^2^CpdI_b_ structure of the cytochrome P450 compound I [147,148,149,150,151,152,153,154,155,156,157,158].

No.	Type	Fe	O	Por	SR	H	C ^(a)^	x	ΔE^‡ (b)^
**II**	^2^CpdI_b_	1.93	−0.20	−0.18	−0.09	0.05	−0.51	51.3	15.8
**IV**	^2^CpdI_b_	1.86	−0.07	−0.19	−0.09	0.03	−0.54	52.2	20.3
**V**	^2^CpdI_b_	1.87	−0.05	−0.22	−0.17	0.02	−0.45	52.4	17.7
**VI**	^2^CpdI_b_	1.85	−0.07	−0.19	−0.08	0.03	−0.54	52.1	20.4
**VII**	^2^CpdI_b_	1.82	0.12	−0.31	−0.24	0.01	−0.40	48.6	15.2
**VIII**	^2^CpdI_b_	1.82	−0.03	−0.22	−0.12	0.02	−0.47	51.7	17.3
**IX**	^2^CpdI_b_	1.83	0.11	−0.29	−0.28	0.00	−0.37	45.9	15.1
**X**	^2^CpdI_b_	1.71	0.21	−0.23	−0.15	−0.01	−0.52	47.0	6.65
**XI**	^2^CpdI_b_	1.81	0.11	−0.30	−0.22	0.01	−0.41	53.3	14.8

^(a)^ carbon radical site of alkyl radical (•CR), ^(b)^ ΔE^‡^: Calculated activation barriers (kcal/mol) for hydrogen radical abstraction (HRA) and hydrogen atom transfer (HAT) (**X**) reactions by the Cpd Ib (^2^E) catalyst.

**Table 11:** Spin densities on the reaction sites of [(Bn-TPEN)Fe(IV)=O]^2+^ obtained by the UB3LYP calculations cyclohexane [186].

State		Fe	O	L ^(a)^	CR_3_	ΔE^#^ * ^(b)^	ΔE^#^ ** ^(c)^
*S* = 0	R	0.54	−0.51	−0.03	−0.00	0.0 (9.1)	0.0 (8.5)
	TS	0.83	−0.30	−0.05	−0.48	13 (14.0)	9.4 (17.9)
	I	0.90	0.09	−0.06	−0.94	−1.3 (−1.8)	−8.3 (0.28)
*S* = 1	R	1.11	0.96	−0.07	0.00	0.0 (0.0)	0.0 (0.0)
	TS	0.96	0.64	−0.07	0.49	8.9 (8.9)	12.5 (12.5)
	I	0.91	0.22	−0.06	0.93	−1.5 (−1.5)	0.87 (0.87)
*S* = 2	R	2.98	0.73	0.29	0.00	0.0 (1.14)	0.0 (0.43)
	TS	3.71	0.24	0.39	−0.34	0.22 (1.36)	7.1(7.5)
	I	3.99	0.38	0.48	−0.85	1.4 (−13.0)	1.4 (−5.6)

^(a)^ L denotes ligand. ^(b)^ Relative energies (kcal/mol) among reactant state (R), transition state (TS) and intermediate (I) in each state (*S* = 0, 1, and 2) under no solvation (*), where relative energies from R state of the triplet state (*S* = 1) are in in parentheses. ^(c)^ The corresponding values for the solvation model (**).

**Table 12:** Relative energies (kcal/mol) between Por(+•)ClFe(IV)=O (^4(2)^A_2_) and PorClFe(V)=O (^2^E) states [103].

Methods	UB3LYP/Basis Set II	CASPT2 [15e, 16o]	RASPT2 [29e, 28o]
^4^A_2_^(a)^	0.0(0.0)	0.0(0.0)	0.0(0.0)
^2^A_2_^(a)^	0.1(0.4)	−1.4 (−0.6)	0.0(0.4)
^2^E ^(a)^	12.4 (12.7)	1.6 (−3.4)	−1.7 (−6.5)
Δ ^(b)^	–0.3	5.0	4.8

^(a)^ The corresponding values for Por(+•)Fe(IV)=O (^4(2)^A_2_) and PorFe(V)=O (^2^E) are given in parentheses, ^(b)^ the Δ value denotes the difference between the PorFe=O with and without the fifth ligand (Cl^−^).

**Table 13:** Relative energies (kcal/mol) between Por(+•)ClFe(IV)=O (^4^A_2_) and PorClFe(V)=O (^2^E) states with solvation energy corrections [103].

Methods	UB3LYP	UB3LYP ^(a)^	UB3LYP ^(a)^	UB3LYP ^(b)^	RAS [29e, 28o]
	(ε = 0.0)	(ε = 5.7)	(ε = 78)	(average)	RAS2 [6 + SO]
^4^A_2_	0.0	0.0	0.0	0.0	0.0
^2^E	12.4	5.7	7.9	6.8 ^(d)^	3.9 ^(d)^ (−1.7)
ΔΔ ^(c)^	0.0	6.7	4.5	5.6 ^(c)^	5.6

^(a)^ The Δ value denotes the difference between the values of UB3LYP (ε = 0.0) and UB3LYP (ε = 5.7, 78), ^(b)^ average ΔΔ value by UB3LYP, ^(c)^ the estimated value by using the average ΔΔ, ^(d)^ the estimated value by using the average ΔΔ for the RAS vale (−1.7 kcal/mol).

**Table 14:** The energy gaps between the triplet (T) and quintet (Q) states, and the activation barriers for hydrogen abstraction reactions (HAR) by the T- and Q-methods [244,245,246].

		ΔE(T-Q) ^(a)^	ΔE^#^(Triplet) ^(b)^	ΔE^#^(Quintet) ^(c)^	C/non C ^(d)^
Methods	Type ^(e)^				
UB3LYP	**XII**	3.1	21.7	10.4	Cross
	**XIII**	7.6	14.3	3.5	Cross
UB3LYP-D3	**XII**	2.5	21.3	10.9	Cross
	**XIII**	10.0	13.3	3.5	Cross
UB3LYP-UCCSD(T)	**XII**	0.6	29.6	17.2	Cross
UHF-UCCSD(T)	**XII**	−2.0	27.6	17.0	Cross
DLPNO CCSD(T)	**XII**	−6.0	30.4	16.7	Quintet
	**XIII**	1.8	25.4	8.8	Quintet
CASPT2	**XII**	−3.3	31.7	15.7	Triplet
	**XIII**	11.4	24.3	8.4	Triplet
CASPT2/CC	**XII**	0.5	29.2	13.7	Cross
	**XIII**	14.0	23.8	9.0	Triplet
CASSCF (12e,12o)	**XII**	−0.5	27.4	16.7	Cross

^(a)^ ΔE(T-Q): triplet (T) and quintet (Q) energy gap; ^(b)^ ΔE^#^(Triplet: activation barriers for the triplet state; ^(c)^ ΔE^#^(Quintet): activation barriers for the quintet state; ^(d)^ Crossing and or non-crossing between triplet and quintet potential curves; ^(e)^ **XII**: (NH_3_)_5_Fe(IV)=O, **XIII**: [(N4Py)-Fe(IV)=O].

## Data Availability

Not applicable.

## Appendix A

### A.1. Localized Natural Orbitals (LNO)

In this review, the BS MO (DFT) model was employed for the main theoretical investigation. Therefore, the theoretical relation between the resonating BS MO (RBS) and the resonating valence bond (RVB) models is briefly revisited [25]. The broken-symmetry (BS) molecular orbitals (MO) in Equation (16) are more or less localized on radical sites, providing largely localized BS MOs at the strong correlation limit (for example, the dissociation limit) as [25]

(A1a) $$\psi_{i}^{+}\left(\theta =\frac{\pi }{4}\right)=\frac{1}{\sqrt{2}}\left(\phi_{i,bonding}+\phi_{i,antibonding}\right)=\phi_{\mathrm{LMOa}}$$

(A1b) $$\psi_{i}^{-}\left(\theta =\frac{\pi }{4}\right)=\frac{1}{\sqrt{2}}\left(\phi_{i,bonding}-\phi_{i,antibonding}\right)=\phi_{\mathrm{LMOb}}$$

where *ϕ*_LMOa_ and *ϕ*_LMOb_ are mainly localized on the radical sites a and b of the molecular systems, respectively. Therefore, Equation (A1a,b) is utilized for the derivation of the localized MO (LMO) and localized natural orbital (LNO) model of unstable molecules such as exchange-coupled transition metal complexes [27,28]. The LMO(LNO)s are important as reference orbitals for the MkMRCC approach [249] without size inconsistent error. It is noteworthy that LMOs (LNOs) are different from the atomic orbitals (AO) in the simple valence-bond (SVB) theory since LMOs are orthogonal and have the characteristics of molecular orbitals.

### A.2. Resonating BS (RBS) Method and Exchange Coupling

As mentioned above, independent particle models (IPM) often indicate broken-symmetry (BS) even for finite systems without phase transition. The concept of quantum resonance in quantum mechanics, however, recovers the symmetry breaking in IPM because of the non-zero overlap between the BS solutions in finite systems. In fact, the resonance concept is familiar in quantum chemistry in relation to the valence bond (VB) theory [25]. In order to elucidate the relationship between the BS MO and VB models, the delocalized MO concept can be transformed into the LMO (LNO) concept for the VB explanation of strongly correlated electron systems (SCES) in Figure 25. The BS MOs in Equation (16) are indeed re-expressed with the LMOs (LNOs) as follows [25]

(A2a) $$\psi_{i}^{+}=\cos\omega \phi_{\mathrm{LMOa}}+\sin\omega \phi_{\mathrm{LMOb}}$$

(A2b) $$\psi_{i}^{-}=\cos\omega \phi_{\mathrm{LMOb}}+\sin\omega \phi_{\mathrm{LMOa}}$$

where the mixing parameter ω is given by *θ* + *π*/4. Therefore, the BSI MO configuration with the (↑↓) spin alignment can be expanded using LMOs as:

$$\Psi_{\mathrm{BSI}}=\left|\psi_{i}^{+}\bar{\psi_{i}^{-}}\right|$$

(A3a) $$=\left|\left(\cos\omega \phi_{\mathrm{LMOa}}+\sin\omega \phi_{\mathrm{LMOb}}\right)\left(\cos\omega \phi_{\mathrm{LMOb}}+\sin\omega \phi_{\mathrm{LMOa}}\right)\right|$$

(A3b) $$=\frac{1}{2}\left\{\sqrt{2}\cos2\omega \Phi_{\mathrm{SD}}+\sqrt{2}\Phi_{\mathrm{TD}}+\sin2\omega \left(\Phi_{\mathrm{ZWa}}+\Phi_{\mathrm{ZWb}}\right)\right\}$$

where the pure singlet (S) and triplet (T) covalent terms (SD and TD) are given by:

(A4a) $$\Phi_{\mathrm{SD}}=\frac{1}{\sqrt{2}}\left\{\left|\phi_{\mathrm{LMOa}}\bar{\phi_{\mathrm{LMOb}}}\right|+\left|\phi_{\mathrm{LMOb}}\bar{\phi_{\mathrm{LMOa}}}\right|\right\}$$

(A4b)
$$\Phi_{\mathrm{TD}}=\frac{1}{\sqrt{2}}\left\{\left|\phi_{\mathrm{LMOa}}\bar{\phi_{\mathrm{LMOb}}}\right|-\left|\phi_{\mathrm{LMOb}}\bar{\phi_{\mathrm{LMOa}}}\right|\right\}$$

On the other hand, zwitterionic (ZW) configurations result from the charge transfer (CT) from *ϕ*_LMOa_ and *ϕ*_LMOb_ (and vice versa) as follows:

(A5) $$\Phi_{\mathrm{ZWa}}=\left|\phi_{\mathrm{LMOa}}\bar{\phi_{\mathrm{LMOa}}}\right|,\;\Phi_{\mathrm{ZWb}}=\left|\phi_{\mathrm{LMOb}}\bar{\phi_{\mathrm{LMOb}}}\right|$$

The low-spin (LS) BSI MO configuration involves the pure triplet covalent term, showing the spin-symmetry breaking property. Similarly, the low-spin (LS) BSII MO configuration with the (↑↓) spin alignment is expressed by:

(A6) $$\Psi_{\mathrm{BSII}}=\left|\psi_{i}^{-}\bar{\psi_{i}^{+}}\right|=\frac{1}{2}\left\{\sqrt{2}\cos2\omega \Phi_{\mathrm{SD}}-\sqrt{2}\Phi_{\mathrm{TD}}+\sin2\omega \left(\Phi_{\mathrm{ZWa}}+\Phi_{\mathrm{ZWb}}\right)\right\}$$

The LS BSII MO solution also involves the pure triplet term. Thus, spin symmetry breaking is inevitable in the case of the single-determinant (reference) BS solution for diradical species. However, both the orbital and spin symmetries are conserved in finite quantum systems, indicating the necessity of recovery of symmetry breaking, as illustrated in Figure 13.

As shown in Equations (A3a,b) and (A6), the BSI and BSII solutions are degenerate in energy. Then, the quantum resonance between them becomes feasible as follows:

(A7a) $$\Phi_{RBS\left(+\right)}=\frac{1}{\sqrt{2}}\left(\Phi_{\mathrm{BSI}}+\Phi_{\mathrm{BSII}}\right)$$

(A7b)
$$=\frac{1}{2}\left\{\sqrt{2}\cos2\omega \Phi_{\mathrm{SD}}+\sin2\omega \left(\Phi_{\mathrm{ZWa}}+\Phi_{\mathrm{ZWb}}\right)\right\}$$

(A8a)
$$\Phi_{RBS\left(-\right)}=\frac{1}{\sqrt{2}}\left(\Phi_{\mathrm{BSI}}-\Phi_{\mathrm{BSII}}\right)$$

(A8b) $$=\Phi_{\mathrm{TD}}$$

where the normalizing factor (N) arising from the non-orthogonality between BSI and BSII is neglected for simplicity. Thus, the in (+)- and out-of-phase (−) resonating BS (RBS) solutions are the pure singlet (S) and triplet (T) states wave functions, respectively. The magnitude of the quantum resonance between BSI and BSII is expressed by the energy gap between the S and T states in Equation (24).
